# Culturally competent healthcare – A scoping review of strategies implemented in healthcare organizations and a model of culturally competent healthcare provision

**DOI:** 10.1371/journal.pone.0219971

**Published:** 2019-07-30

**Authors:** Oriana Handtke, Benjamin Schilgen, Mike Mösko

**Affiliations:** Department of Medical Psychology, University Medical Center Hamburg-Eppendorf, Hamburg, Germany; FHI360, UNITED STATES

## Abstract

**Background:**

Culturally and linguistically diverse patients access healthcare services less than the host populations and are confronted with different barriers such as language barriers, legal restrictions or differences in health beliefs. In order to reduce these disparities, the promotion of cultural competence in healthcare organizations has been a political goal. This scoping review aims to collect components and strategies from evaluated interventions that provide culturally competent healthcare for culturally and linguistically diverse patients within healthcare organizations and to examine their effects on selected outcome measures. Thereafter, we aim to organize identified components into a model of culturally competent healthcare provisions.

**Methods and findings:**

A systematic literature search was carried out using three databases (Pubmed, PsycINFO and Web of Science) to identify studies which have implemented and evaluated cultural competence interventions in healthcare facilities. PICO criteria were adapted to formulate the research question and to systematically choose relevant search terms. Sixty-seven studies implementing culturally competent healthcare interventions were included in the final synthesis. Identified strategies and components of culturally competent healthcare extracted from these studies were clustered into twenty categories, which were organized in four groups: Components of culturally competent healthcare–Individual level; Components of culturally competent healthcare–Organizational level; Strategies to implement culturally competent healthcare and Strategies to provide access to culturally competent healthcare. A model integrating the results is proposed. The overall effects on patient outcomes and utilization rates of identified components or strategies were positive but often small or not significant. Qualitative data suggest that components and strategies of culturally competent healthcare were appreciated by patients and providers.

**Conclusion:**

This scoping review used a bottom-up approach to identify components and strategies of culturally competent healthcare interventions and synthesized the results in a model of culturally competent healthcare provision. Reported effects of single components or strategies are limited because most studies implemented a combination of different components and strategies simultaneously.

## Introduction

The United Nations state “the world is on the move, and the number of international migrants today is higher than ever before.” [1]. The associated growing diversification of societies offers many opportunities for societal and economic growth but often presents a challenge for receiving countries. Consequences can include inequalities and discrimination in different areas [2]. The European Union (EU) and the Constitution of the World Health Organization (WHO) ratified the universal right to health as a fundamental human right. Nevertheless, inequalities in access to healthcare exist worldwide and are related to the legal and socioeconomic status of each individual and the laws and policies of each country [3, 4]. In fact, culturally and linguistically diverse patients (CLDP) access healthcare services less than the host populations and are confronted with different barriers [3–7]. These barriers include the organization and complexity of healthcare systems, legal restrictions on access to certain health services, linguistic and cultural barriers, discrimination and limited competencies or unawareness of providers. These are often intertwined with individual factors such as low health literacy, employment status, fear of stigma, language barriers or differences in health beliefs and behaviors [2–7]. Betancourt identified three levels of sociocultural barriers to healthcare: organizational barriers, structural barriers and clinical barriers. Organizational barriers, which affect availability and acceptability of healthcare for CLDP, refer for instance to the degree to which the population’s cultural and linguistic diversity is represented in the leadership and workforce of healthcare organizations. Structural barriers emerge from the complexity and bureaucracy of healthcare systems. Specifically, the absence of interpreter services and of culturally and linguistically adapted materials, increased wait times among CLDP populations and problems in referrals to specialist care cause dissatisfaction and inequalities. Clinical barriers occur in patient/provider interactions and can be seen as sociocultural differences which are not identified, accepted or understood. These can lead to mistrust, dissatisfaction, decreased adherence and poorer health outcomes [8].

The implementation of cultural competence in healthcare facilities seemed to be the answer to these disparities, and traditional receiving countries have been working towards it [8–12]. Indeed, the demand for culturally competent healthcare systems has reached the political levels of diverse countries. The National Culturally and Linguistically Appropriate Service Standards (CLAS Standards) were introduced in 2000 in the United States [13], and in 2005 the Australian government published “Cultural competency in health: A guide for policy, partnerships and participation” [14]. In 2007 the “cultural opening” of healthcare facilities was demanded by a representative of the German federal government [15] and the NHS has offered the migrant health guide since 2014 [16].

There are different definitions, names and implementation guidelines for the concept of cultural competence or cultural competency [12, 17]. The most commonly used definition is the one by Cross et al. (1989): „Cultural competence is a set of congruent behaviors, attitudes, and policies that come together in a system, agency or among professionals and enable that system, agency or those professions to work effectively in cross-cultural situations” [18]. This definition emphasizes that cultural competence is implemented on different levels of care. Corresponding to their identified barriers, Betancourt et al. (2003) differentiate between three levels of interventions: organizational, structural and clinical cultural competence interventions [8]. Fung et al. (2012) take a systemic approach and define cultural competence on macro, meso (institutional and programmatic) and micro levels, by which macro reflects the societal level, meso the organizational and micro the individual clinical level [10]. The German concept of “cultural opening”describes the process of adapting or “opening”facilities and is hence a process of organizational development which includes interventions on different levels within facilities [19].

Existing systematic reviews have focused on defining theoretical concepts [8, 9, 20] or on the effectiveness of cultural competence interventions [11, 12, 17, 21, 22]. Individual cultural competence among healthcare providers was examined most frequently [12, 20]. A systematic review of reviews on cultural competence in healthcare found moderate positive effects of individual cultural competence trainings on provider outcomes (knowledge, skills, attitudes) and on access and utilization outcomes but only weak effects on patient outcomes (satisfaction, health status) [12]. Other interventions that were often identified by authors of existing reviews were the recruitment of bilingual staff, the use of interpreters and the translation of treatment materials [9, 11, 12, 20]. All together these reviews were not able to determine the effectiveness of interventions because of the lack of comparative studies and objective outcome measures [11, 12, 17, 21–23]. A number of systematic reviews were conducted which often focused on conceptual models and definitions or broad categories of cultural competence and derived interventions or strategies from those. We chose a bottom-up approach in order to extract culturally competent components or strategies from healthcare interventions designed to be culturally competent. The methodology of a scoping review appears appropriate for capturing the presumed diversity of components and strategies to provide culturally competent healthcare to CLDPs.

This scoping review aims to collect components and strategies from evaluated interventions that provide culturally competent healthcare for CLDPs within healthcare organizations and to examine their effects on selected outcome measures. Thereafter, we aim to organize identified components into a model of culturally competent healthcare provisions.

## Methods

The review was guided by the question “What are components or strategies extracted from evaluated culturally competent healthcare interventions that were designed to provide healthcare for culturally and linguistically diverse patients (CLDP) in healthcare organizations?”

### Search strategy

A systematic literature search was carried out in following databases: Pubmed, PsycINFO and Web of Science. The search was conducted in August 2016 and updated in January 2017 to include studies published during/after August 2016. No restrictions were set. Furthermore, lists of references of relevant articles were manually examined for the purpose of identifying further eligible studies.

The PICO criteria were adapted [24] in order to formulate the research question and to systematically choose relevant search terms. We concentrated on the criteria Population (e.g., migrants, culturally and linguistically diverse patients), Intervention (e.g., program, standard, strategy) and Outcome (e.g., increasing cultural competences or cross-cultural opening). The search string is available in the S1 File. More precisely, we searched for studies which evaluated cultural competence interventions quantitatively or qualitatively in order to increase cultural competence in healthcare facilities. Additionally, we included the criterion Setting (e.g., hospitals, clinics, health centers) because we were exclusively interested in interventions implemented in healthcare facilities. Analyses of Medical subject Headings (MeSH) and of key terms of related articles were used to identify search terms. These were discussed by the authors and combined to a search string, which was adapted for each database. As recommended by Arksey and O’Malley (2005) we started the search with a wide approach in order to create a comprehensive map of the field.

### Eligible criteria and assessment

The selection process was divided into two screening phases. First, a screening of titles and abstracts was conducted followed by full text screening.

In the first screening phase, studies evaluating interventions located at healthcare organizations and aiming to improve cultural competence of healthcare facilities and/or healthcare for CLDP were included. Studies reporting the existing level of cultural competence of healthcare facilities or studies evaluating interventions in other facilities (e.g., schools, community centers) were excluded. In the event that the setting of the intervention was not identifiable in title or abstract, studies were nonetheless included in order to be examined in full text screening. Studies evaluating cultural competence trainings on an individual provider level were excluded because systematic reviews have already shown their positive effect on provider outcomes (e.g., knowledge, skills and attitudes) and their satisfying effect on patient outcomes (e.g., satisfaction, health status) [12]. At this stage all study types as well as all publication types except for reviews and meta-analysis studies were deemed eligible.

The title and abstract screening was carried out by three independent raters. Prior to this first screening phase all raters screened 100 randomly chosen articles each and reached an interrater reliability of ĸ = 0.7 (main author—first rater) and ĸ = 0.8 (main author—second rater). Disagreement was discussed in regular meetings and screening criteria were specified along the screening process.

Eligible criteria for full text screening were specified and iteratively adapted during the second screening phase [25]. The criteria were divided into the following categories: Design, Recipient Population, Content, Method and Context. The category Design (criterion 1) included only studies with a sample size of more than two and only studies using primary data. Hence, reviews, meta-analysis studies, study protocols and letters to the editors were excluded. The Recipient Population consisted of migrants, CLDP, ethnic minorities (e.g., Latino population, Native Americans, South Asian Americans) or refugees (criterion 2). In order to be considered eligible regarding their content, studies had to examine interventions that aim to improve healthcare utilization, provision or treatment for CLDP and/or cultural competence in healthcare facilities (criterion 3). Additionally, they needed to be evaluated with quantitative or qualitative research methods (Method; criterion 4). Furthermore, studies that only focus on (psychometric) evaluation of instruments were not eligible. Interventions had to be implemented explicitly in inpatient or outpatient settings such as hospitals; health or medical centers; health facilities; health organizations; (medical) trusts or sites or clinics in order to meet the Context criterion (criterion 5). If study participants were recruited in healthcare facilities, but the intervention was located elsewhere, these studies were excluded. Studies located in general practice or community centers were excluded, as well. Only studies published in English or German and meeting all criteria were included. Detailed screening criteria are available in the S1 Table.

In the full-text screening the remaining articles were screened by two independent researchers. They reached an interrater reliability of k = 0.8, which was considered to be satisfying. Both raters met on a regular basis throughout the screening process to ensure a high level of consensus and to discuss any uncertainties.

### Data extraction and summary

Data extracted from the studies were summarized into two spreadsheets. One spreadsheet describing the characteristics of healthcare interventions included following information: Authors, name and location of the intervention, target group and components of the interventions. (S2 Table) [26]. The second spreadsheet incorporated study characteristics and results: outcome measures, study type, study participants (N, ethnicity) and main results (S3 Table). To assure the accuracy of extracted data, they were verified by two independent researchers. Single components and strategies for providing culturally competent healthcare extracted from studies were clustered and organized into a model. In order to determine their effects, studies were checked for results relating to single components. Descriptive statistics were used to summarize the data.

## Results

### Study selection

**Fig 1:**
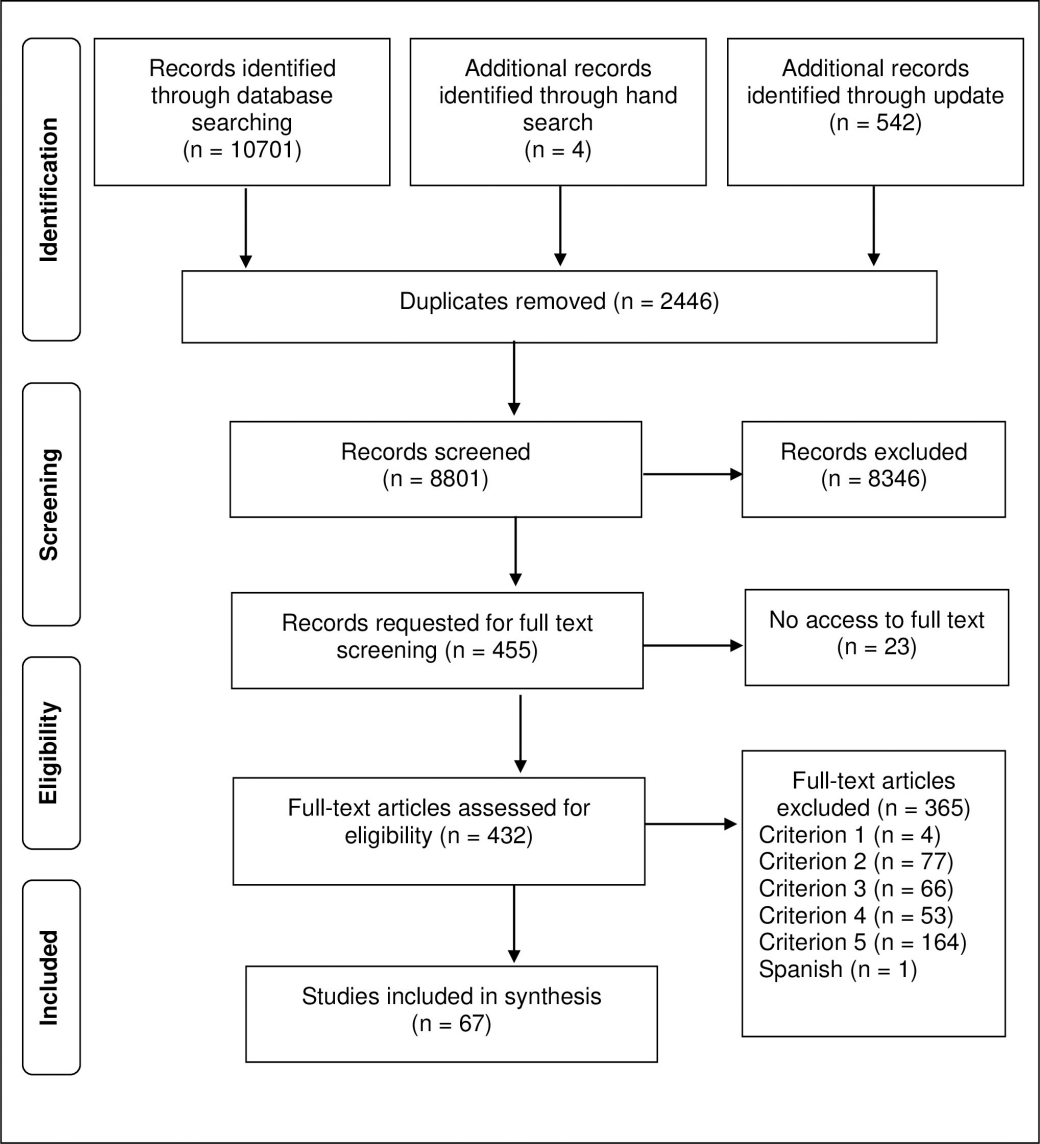
PRISMA flowchart.

The initial search in the databases provided a total of 10,701 citations. Through the update in January 2017, an additional 542 publications were found. Four articles were added from a manual search. After adjusting for duplicates 8,801 records remained for the title and abstract screening, and 455 studies met criteria for inclusion in the first selection phase. 23 articles then had to be discarded because the full-text publications of the studies were not available. 432 articles were finally included for the full-text screening. Of these, 67 met the inclusion criteria in the second selection phase and were eligible to be included in the final synthesis (Fig 1).

### General study characteristics

The general characteristics of included studies are summarized in Table 1. Studies included in the final synthesis were published between 1990 and 2017 and were all written in English.

**Table 1:** General characteristics of included studies (n = 67).

Characteristics	Number of studies (%)
**Country of origin**	
United States	51 (76)
Europe	6 (9)
Canada	5 (7.5)
Australia/New Zealand	3 (4.5)
Others	2 (3)
**Medical fields**	
Mental health	19 (28)
Diabetes care/prevention	11 (16.5)
Pediatric care	8 (12)
Cancer care/prevention	7 (10.5)
Pregnancy care and postnatal care	4 (6)
Hypertension care	2 (3)
No specific medical field	13 (19.5)
Others	3 (4.5)
**Study design**	
Quantitative design	45 (69)
Mixed-Method design	15 (21)
Qualitative design	7 (10)
**Participants’ ethnicities**^a^	
Latinos/Hispanics	32 (48)
African Americans	22 (33)
Whites	19 (28)
Asians (not specified)	9 (13)
People from South Asia	8 (12)
People from East Asia	8 (12)
People from African countries	7 (10.5)
People from Southeast Asia	6 (9)
Native populations	6 (9)
Other	17 (25)
Ethnicity not specified	5 (7.5)
**Outcome measures**	
Patient outcomes	
Psychological health	18 (27)
Physical health	14 (21)
Patient satisfaction / experience with intervention	14 (21)
Health literacy / patient empowerment	12 (18)
Treatment adherence	9 (13)
Psychosocial outcomes	8 (12)
Learned health behaviors	3 (4.5)
Perceived cultural competence / sensitivity	2 (3)
Others	3 (4.5)
Provider outcomes	
Cultural competence	3 (4.5)
Satisfaction with intervention	2 (3)
Knowledge in targeted condition	2 (3)
Practice change	1 (1.5)
Utilization, coverage and access outcomes	
Utilizations rates of healthcare	9 (13)
Rates of cancer screening	4 (6)
Improvements in care	3 (4.5)
Improvements in access	2 (3)
Others	3 (4.5)
Organizational outcomes	
Workforce diversity among staff	2 (3)
Costs of the intervention	2 (3)
Organizational cultural competence	1 (1.5)
Diversity climate	1 (1.5)
Feasibility, acceptability and utility of the interventions	12 (6)

^a^ 33 studies included more than one ethnic group

The majority of studies were conducted in the United States (n = 51; 76%). 28% of the interventions were implemented in the mental health field including substance abuse and neuropsychology (n = 19), followed by 16.5% implemented in diabetes care/prevention (n = 11). Included studies applied a quantitative study design in 69%, a mixed method design in 21% and 10% were qualitative studies. Fourteen studies were randomized controlled trials (RCTs) [27–40] with two cluster randomizations [27, 28]. RCTs compared one to three interventions with the control intervention, which was typically treatment as usual. Twelve studies were interrupted time series studies [41–52]. Other study types included controlled before and after studies [53–60], historically controlled studies [61–63], cross-sectional studies [64–67], cohort studies [68, 69] and incidence studies [70, 71]. All mixed methods studies used congruent triangulation [72–85], except for one that chose a sequential transformative design [86]. Qualitative studies included four case studies [87–90], and the methods of data collection used were interviews [90–92], focus groups [89, 93] or open-ended questions [88]. The number of participants ranged from 6 [41] to 5963 [43] participants. Twenty-nine studies focused on one ethnic group [27, 30–36, 39, 41, 42, 47–49, 51, 57, 69, 75, 77–80, 82–85, 87, 89, 92] while thirty-three included more than one ethnic group [28, 29, 37, 38, 40, 43–46, 50, 52–56, 58–68, 70–73, 86, 91, 93] and five studies did not report the ethnicity of their participants [74, 76, 81, 88, 90].

Measured outcomes were patient outcomes, provider outcomes, organizational outcomes and utilization, coverage and access outcomes [12]. Psychological health outcomes such as reduction in symptoms of mental illness [28, 33, 35, 36, 47, 48, 52, 59, 61, 85] and in regard to health, concepts such as self-efficacy [34, 73, 86] or distress [49, 51] were assessed the most frequently.

### Categorizations of identified culturally competent components and strategies

Extracted components and strategies of the 67 culturally competent healthcare interventions were clustered into 20 sub-categories of components, which were then grouped into four categories: (1) Components of culturally competent healthcare within facilities–Individual level; (2) Components of culturally competent healthcare within facilities–Organizational level; (3) Specific strategies to provide access to culturally competent healthcare; (4) Strategies to implement culturally competent healthcare within facilities. Descriptions of components and strategies including the references can be found in Table 2. The components of identified culturally competent healthcare interventions and their assigned categories are available in the S4 Table. The component “Cultural and linguistic matching” was identified the most, more precisely in 29 studies, followed by “Use of culturally adapted/appropriate written or visual material”, which was found in 27 studies. The component “Involvement of the facilities’ leadership” as a strategy to implement culturally competent healthcare within facilities was identified the least, specifically in 3 studies. Almost 80% of the interventions were located in outpatient facilities. Only eleven studies collected data in inpatient settings.

**Table 2:** Identified components to provide culturally competent healthcare.

*Identified components*	*Number of studies*
**Components of culturally competent healthcare within facilities–Individual level**	
***1*. *Linguistic and/or cultural matching***: Medical professionals are bilingual/bicultural and/or representatives of the target community and share the same cultural background as their patients [28, 30, 33, 35–38, 41–43, 47–49, 51, 52, 56, 57, 59, 61, 64, 65, 68, 69, 71, 73, 77, 82, 84, 87]	*29*
***2*. *Incorporation of culturally specific concepts into individual contacts***: culturally specific aspects are integrated into the one-on-one contact between patients and providers:	*22*
- Patients’ problems, explanatory models [27, 36, 40, 41, 61, 82, 91]	7
- Perceptions of access barriers into healthcare [28, 52, 62]	3
- General cultural values or norms	10
- Experiences caused by migration such as acculturation stress or racism [36, 41, 53, 85, 91]	5
- Use of culturally specific language patterns	
○ the use of dichos–“Spanish language proverbs and sayings” [41, 87]	2
○ following common verbal and nonverbal communication norms [36, 41, 87]	3
- Use of Specific culturally competent communication methods	
○ ethnographic methods [75]	1
○ construction of illness narratives [82]	1
○ the Culturally Enhanced Video Feedback Engagement (CEVE) [40]	1
○ intervention or the Engagement Interview Protocol (EIP) [82]	1
○ Cultural Formulation Interview (CFI) [91]	1
- Race-specific data [38]	1
***3. Use of culturally and linguistically adapted/appropriate written or visual material***: materials are linguistically and culturally adapted:	*27*
- Educational and therapy written materials or handouts [27–31, 36, 38, 39, 42, 47, 49, 51, 54, 55, 60, 66, 71, 72, 75, 76, 78, 82, 83, 86]	24
- Videos [34, 37, 39, 51, 72]	5
- Therapy manuals [47]	1
- Consent forms [54]	1
- Policy brochures [55]	1
- Patient satisfaction surveys [76]	1
- Screening instruments [52]	1
Different strategies of adaptation were identified:	
- Translating the materials into different languages [27, 30, 34, 37, 42, 52, 72, 75, 78, 83]	10
- Adapting them to low literacy and education levels [29, 30, 34, 42, 49, 60, 72, 75, 83]	9
- Including culturally sensitive treatment recommendations [27, 47, 49, 51]	4
- Integrating illustrations of characters from target communities [34, 38, 42, 51]	4
- Addressing barriers to care [28]	1
- Integrating culturally specific art into intervention material [31]	1
***4. Involvement of families***: Families are informed or involved in the treatment process [61, 62, 65, 73, 85]	*5*
***5. Continuity of care***: Patients are offered further support after their initial treatment through:	*13*
- Referral to specialized facilities which offer further culturally appropriate support [27, 28, 60]	3
- Telephone calls	
○ as reminders or follow-ups after or before an intervention [28, 32, 56, 57, 77]	5
○ to offer further support [33, 83]	2
- Home visits [43]	1
- Communication with or referral to primary care provider [32, 33, 57, 82]	4
- Sending out postcards or mail [77]	1
- Follow-up in clinic visits [52, 82]	2
- Giving out records/documents to patients to continue care themselves or at another place [71]	1
**Components of culturally competent healthcare within facilities–Organizational level**	
***1. Cultural competence training for healthcare providers***: Providers receive training to improve their ability to work efficaciously with CLDP [27, 45, 54, 55, 60, 76, 79, 80, 82]	*9*
***2. Human resources development***: Changes in staff are implemented to meet healthcare needs:	*12*
- Recruitment of bilingual and bicultural staff/oversea staff [43, 50, 54, 60, 61, 67, 93]	7
- Capacity building (individuals from target communities are recruited and medically trained) [71, 77, 81]	3
- Creation of a new position as reference-nurse in charge of migrant care issues [46]	1
- Expansion of the role of pharmacist to treating five minor pediatric conditions [66]	1
***3. Integration of interpreter services***: Language interpretation is made available [46, 50, 54, 57, 58, 60, 75, 76, 82, 88, 89]	*11*
***4. Adaption of the facility’s social and physical environment***: The health facility’s environment and organization are appropriate for CLDP	*11*
- Provision of cultural foods used	
○ to educate participants about healthy eating [42, 49]	2
○ as an opportunity for participants to socialize [75, 80]	2
○ to increase well-being in clinic settings [63, 88]	2
- Making the complaints procedure available in all languages [88]	1
- Integrating a 15-minute prayer break into support group [75] - Changing the facility’s physical environment	1
○ display of culturally sensitive calendars, magazines, comment cards, bilingual restroom signs, posters, art featuring people from different cultures, displaying toys for patients’ children [55, 58]	2
○ decoration with art from refugee’s native countries [81]	1
○ placement of twelve kiosks in a hospital offering multilingual help to patients and visitors [76]	1
○ instalment of a sweat lodge [70]	1
***5. Patient data collection and management***: Patient data are collected to	*12*
- Better tailor care to individual patients [32, 46, 57, 58, 60, 62, 65, 73]	8
- Monitor frequency of contact with patients from migrant groups [88]	1
- Identify potential patients or individuals at risk [71, 73, 77, 92]	4
**Strategies for providing access to culturally competent healthcare**	
***1. Integration of community health workers (CHW)*:** Peer or community workers are integrated into care to	*20*
- Educate patients during home or clinic visits [29, 30, 32, 39, 42, 44, 56, 73, 83]	9
- Help patients navigate the system [43, 57, 65, 68, 81, 89, 90, 92]	8
- Mediate between patients and providers [62, 74, 79, 83, 89]	5
***2. User engagement and networking***: Strategies to assure cultural appropriateness of healthcare interventions and/or reducing barriers by cooperating with	*10*
- Target communities [37, 42, 45, 51, 54, 70, 86]	7
- Institutions [77, 88]	2
- Facilities engaging in the same process [61]	1
- International medical graduates in training [50]	1
In order to	
- Reduce access barriers [37, 42, 54, 77]	4
- Assure cultural appropriateness [37, 42, 50, 51, 70, 86]	6
- Obtain guidance/consultation [45, 61, 77, 88]	4
***3. Telemedicine***: Healthcare is provided through videos or webcam to overcome limited access to culturally and linguistically appropriate treatment:	*8*
- Offering treatment with a psychiatrist through webcam communication [35, 84]	2
- Offering education or prevention interventions through videos [34, 37, 38, 58]	4
- Offering education or prevention interventions through computer-based written information [78, 86]	2
***4. Outreach methods***: Any type of health service that mobilizes healthcare workers to provide services to the population, outside of the location where they usually work and live	*11*
- Mailed packages [31–33, 58, 92]	5
- Home visits [30, 32, 38, 71, 83]	5
- Telephone calls [30, 92]	2
- Remote clinics [58, 77, 79]	3
***5. Creating community health networks***: Health facilities engage in activities concentrating on cooperation and exchange with other institutions within communities [43, 50, 54, 71]	*4*
**Strategies for implementing culturally competent healthcare within facilities**	
***1. Needs assessment and monitoring of organizational changes***: Strategies for planning and monitoring organizational changes	*8*
- Organizational level assessment [45, 60, 76]	3
- Assessment of provider needs or barriers [72, 76, 88]	3
- Assessment of patient needs [81]	1
- Assessment with patients, providers and representatives of target communities [70]	1
- Assessment of the main language groups [88]	1
***2. Creation of positions or groups to monitor and supervise the process***	*6*
- Diversity coach [60]	1
- Multicultural consultation group [88]	1
- “Cultural competence committee” [45]	1
- “Interdepartmental and interprofessional working group”(“Health for All Network“) [46]	1
- “Steering group committee to the ethos of WHO/UNICEF Baby friendly hospital initiative”[72]	1
- “New Immigrant Support Network“(NINS) [76]	1
***3. Development of action plans***: Specific goals and strategies of implementation of cultural competence are recorded [45, 60, 72, 88]	*4*
***4. Leadership involvement and support***: Leadership is involved in the process of implementing cultural competence to support and promote the process [45, 60, 76]	*3*
***5. Promoting structural changes within the organization***: Strategies to promote structural changes among their staff members to assure their implementation	*7*
- Adaptations of procedures and policies [72]	1
- Brief presentations of changes/process to staff members [46]	1
- Distributions of brochures presenting changes [46]	1
- Public events promoting changes [46]	1
- Signalling to staff and stakeholders that cultural competence is a high priority [76]	1
- Protected time was administered to staff members to attend cultural competence training [78]	1
- Establishment of a competition program [58, 76]	1

### Effects of identified components and strategies

Quantitative and qualitative results from studies implementing the identified components or strategies are reported below. Importantly, only the results which indicate a relation between an isolated component or strategy and an outcome measure are considered. Results related to the implementation of multiple components or strategies simultaneously are not reported because in this case the effects of single components or strategies on outcome measures cannot be confirmed.

### Components of culturally competent healthcare–Individual level

**Linguistically and cultural matching.** After the recruitment of a bilingual Russian internist at the Denver Medical Center (USA) with the goal of improving diabetes care for Russian patients, there was a significant reduction in diastolic blood pressure and cholesterol (p < .0002) among Russian diabetes patients. HbA1c and systolic blood pressure also decreased, albeit not significantly [69]. The Portuguese-speaking patients of a clinic within an urban safety-net hospital system in the US where 95% of staff members spoke Portuguese, were more likely to receive adequate care with a difference of 28% compared with patients receiving care in other clinics. No differences were found for emergency room use and inpatient care [64]. Hispanic clients with severe mental illness treated by a Hispanic clinician in the context of assertive community treatment in the US, showed less improvement in symptoms of psychosis than those treated by a White clinician (p = .001). Interactions for other outcomes were not significant [59]. Patients in a culturally focused psychiatric consultation intervention program for Latino Americans with depression agreed that it is more important that their providers speak their language than that they have the same cultural background [82].

**Incorporation of culturally specific concepts.** At the Martha Eliot Health Center (USA) Latino/a patients with anxiety participating in an allocentric (“the tendency to define oneself in relationship to others”) relaxation intervention, which was considered more appropriate for the Latin culture, practiced allocentric imagery exercises significantly (p < .01; M = 3.1, SD = 1.8) more often than idiocentric imagery exercises (M = 2.1, SD = 2.2) [48]. No group changes in postpartum depressive symptoms were identified compared to treatment as usual after a preventive postpartum depression intervention that put value on integrating different aspects of Latino culture at a public sector women’s clinic (USA). Nonetheless, long-term rates of major depression were lower (14% vs. 25%) representing a small effect size (h = 0.28) [36]. Aviera (1996) noted that the use of dichos, Spanish language proverbs, in a therapy group for Spanish speaking psychiatric in-patients in the US are useful for “building rapport, decreasing defensiveness, enhancing motivation and participation in therapy, improving self-esteem, focusing attention, facilitating emotional exploration, articulating feelings, developing insight, and exploring cultural values and identity” [87] for Spanish-speaking patients. The CEVE is a “one-session clinical intervention that integrates the use of shared observations of videotaped interactions with the cultural framing of the family’s problem in a culturally congruent manner”. Families receiving the CEVE at an outpatient clinic in the US reported significantly higher ratings for therapeutic alliance and perceived therapist cultural competence (F(1,15) = 10.03; p < .01) [40]. The Cultural Formulation Interview, “a cross-cultural assessment tool”, was considered useful in eliciting data to determine the nature of the problem from patients’ perspectives, developing and maintaining the therapeutic relationship and communication, educating the patient and in implementing treatment plans at New York Presbyterian Hospital (USA) [91].

**Use of culturally and linguistically adapted/appropriate written or visual material.** Watching characters with the same cultural background in an educational telenovela intervention for diabetes patients in the context of the SHL-program (Sugar, Heart, and Life) at four community health centers in the US led to a mix of low and high levels of viewer identification among participants and to improved feelings of self-efficacy: 17% indicated general optimism or motivation for engaging in diabetes self-care, and 10.5% indicated a specific plan for behavior change [34].

**Involvement if families.** Parental satisfaction with a family-centered intervention for children within the Pediatric Resident Continuity clinic at the Mattel Children’s Hospital (USA) were 8.5 points higher for Spanish-speaking families then for English-speaking families, albeit not significantly (*p* = .003) [65]. In the context of a community based approach to diabetes control at multiple heath centers in the US, focus group discussions suggest that through family involvement patients from African American, Latino or Asian background “felt they were better able to treat their disease, that they were more comfortable talking about their diabetes with their families and friends, and that they felt more confident and in control of their lives”. [73].

### Components of culturally competent healthcare–Organizational level

**Cultural competence trainings for providers.** In a patient-centered culturally sensitive healthcare intervention program based in two community-based primary care clinics in the US cultural sensitivity ratings of providers’ behaviors and attitudes by African American patients increased significantly (*F* (1, 14) = 4.549, p = .05) after provider training and more at the intervention clinic than at the control clinic, however, the differences were not significant [55]. Also, significant increases in providers’ self-rated knowledge (*t*(34) = -7.96, p < .001), awareness (*t*(34) = -6.79, p < .0019) and skills (*t*(34) = - 4.49, p < .001.) in cultural competence was observed after a bilevel cultural competence intervention at a community mental health center in the US [45]. Furthermore, providers receiving training within the National Center for Healthcare Leadership Diversity demonstration project in two US hospitals presented greater changes on all three individual level competencies–increase in diversity attitudes, decrease in implicit bias, and increase in racial/ethnic identity–than providers in the control hospital [60].

**Human resources development.** Two interventions focused on the integration of qualified oversea nurses and midwives in Australia and the UK. The authors described the integration process of oversea nurses and offered support from the recruiting organizations. More than 90% of the questioned oversea nurses found the support strategies useful, especially personal support and a welcoming atmosphere upon arrival and orientation. All nurses who were supported by the program remain employed [67]. Stakeholders found that the program was resource-intensive and questioned the cost-effectiveness of this method for meeting employment needs. Senior nurses and many ward managers thought it beneficial to promote the ethnic diversity of the nursing workforce [93]. Another study concentrated on the expansion of the role of pharmacists in treating five minor pediatric conditions in a pediatric clinic (USA). Service provided by pharmacist was comparable to the service provided at the standard acute care clinic; patients were more likely to have shorter wait time (<15-minute wait) and were more likely to receive written information than patients evaluated by physicians. In addition, patient satisfaction was high [66].

**Integration of interpreter services.** Bekaert reports that even though a language and advocacy services was installed at Horton General Hospital (UK), relatives were still translating for patients due to costly systems [88]. Furthermore, some patients in a culturally focused psychiatric consultation intervention program for Latino Americans with depression reported that even though interpreters were available, waiting for interpreters or having interpreters involved in private medical conversations was challenging [82].

**Adaption of the organization’s social and physical environment.** After the implementation of a Cambodian menu for postpartum women at Saints Medical Center (USA) in combination with a staff training program on breastfeeding, there were no significant difference between breastfeeding initiation rates among Cambodian women and non-Cambodians (66.7% Cambodian vs.68.9% non-Cambodian p = .874), although before its implementation Cambodian mothers were significantly less likely to initiate breastfeeding than non-Cambodian mothers (16.7% Cambodian vs. 60.6% non-Cambodian p = .003) [63]. The option of having an ethnic meal was not chosen because patients did not trust the mechanism of provision at Horton General Hospital (UK) [88], and it was considered enjoyable but not essential by women refugees at an ambulatory healthcare facility [75]. The installment of a sweat lodge on hospital property, where traditional ceremonies were held, improved care for Native Americans, which resulted in increased admissions of this population (4.77% to 7.50%) [70].

**Data collection and management.** Bekaert et al. (2000) reports that at Horton General Hospital (UK) “data collection was still not carried out regularly because staff felt it would be an imposition.”

### Strategies for providing access to culturally competent healthcare

**Integration of community health workers (CHW) to educate patients during home or clinic visits.** CHW were generally bicultural/bilingual and were also able to conduct minor medical procedures. Culica et al. (2008) found significant reductions (p < .05) in mean HbA1c levels of culturally diverse diabetes patients from baseline to six months (8.14% to 7.36%) and 12 months (8.14% to 7%) after attending educational clinic visits carried out by a CHW at an urban community clinic (USA). In the context of a clinic-based colorectal cancer screening promotion program (USA), the integration of a CHW in combination with mailed educational material on colorectal cancer screening increased the number of screenings to 31% compared to 26% in the control group among Hispanic patients but the differences were not significant (p = .28) [30]. In the study by Tu et al. (2006), a culturally competent clinic-based educational program promoting fecal occult blood testing (FOBT) screenings among Chinese patients including motivational videos on colorectal cancer screening and carried out by a trilingual and bicultural health educator increased the screening rate to 69.5% compared to 27.6% in the treatment as usual condition [39]. A culturally and linguistically tailored health coach intervention for Chinese-American diabetes patients at two outpatient medical care units, in which patients were closely accompanied by their health coach during and after treatment, resulted in decreased mean HbA1c levels at follow-up (-0.40%) compared to the treatment as usual group (+0.04%), however this difference was not statistically significant [57]. Black or Latina navigators were integrated at Capital Breast cancer Center (USA) for women with abnormal mammogram results to ensure follow-up screenings among a population with low screening rates. Due to the intervention, 80% of women in need of further screening returned within a median time interval of 39 (range: 6–400) days which is below the recommended time of 60 days [68]. The advocates or liaisons who are integrated into the clinic team work lower stress for patients and providers through improved communication, increased safety of treatment, improved understanding, trust and connectedness, which in turn leads to higher efficacy of treatment and greater improvements in applying health recommendations in an outpatient oncology clinic (USA) as well as in primary care community clinics (Israel) [74, 79].

**Telemedicine.** Psychiatric treatment offered by a bilingual psychiatrist via webcam led to a significant reduction in symptom severity and disability ratings as well as improvements in quality of life over time (p>.001) among Hispanic patients at a community health center (USA), but differences to treatment as usual delivered by a primary health provider were not significant [35]. The ratings of acceptability on a five-point-Likert scale ranged between 3.19 to 4.69, showing a high acceptability among Korean-speaking patients who were treated by a Korean-speaking psychiatrist via webcam at two mental health centers in the US [84]. Multilingual educational videos were significantly more beneficial than usual care (p>.001) for Punjabi and Chinese patients at a university-based pulmonary medicine clinic in Canada [37].

**Outreach methods.** An RCT by Coronado (2011) shows that a culturally competent mailed colorectal screening packet led to a 26% screening rate compared to 2% in clinic-based usual care (p < .001) and to a 31% screening rate if combined with telephone reminders and an educational home visit by a health promoter and a medical assistant (p < .001). Additionally, patients assigned to a home-based educational program on Iiving donor kidney transplantation in addition to a clinic based program at Shands Hospital at the University of Florida (USA) were more likely to have had living donor inquiries (OR:1.7; CI = 1.2–3) and a living donor evaluation (OR:2.7; CI = 1.4–5.4) and live donor kidney transplantation (OR: 3.0; CI = 1.5–5.9) than patients in the clinic-based program only [38]. Watkins et al. (1990) developed a strategy of early case finding by visiting women enrolled in their project and providing them with guidelines to identify culturally diverse pregnant farmworker women and referring them to a migrant health center in North Carolina (USA), which increased prenatal visits from a mean of 7.4 to a mean of 9.7 over one year and decreased the number of children with low birth weight from 13 to 6 over 2 years.

**Creating community health networks.** In order to improve healthcare for CLDP, health facilities engaged in activities concentrating on cooperation and exchange with other institutions within communities. In the context of a health clinic for refugees in Canada, initial intake assessments and basic services were performed at the reception house by case workers and trained professionals, while comprehensive care was provided at the refugee health clinic and more specialist services by community providers. Language support was also provided by the reception house. As a result, the likelihood of an individual requiring a physician specialist went down 45% as a result of seeing a refugee health clinic physician (OR = .55; p = .004) and refugees’ wait time to see a healthcare provider decreased from 30 to 21 days (Ratio of mean = .70; p < .001) [50]. A regional health collaborative formed by New York Presbyterian aimed to create a “medical village” by transforming clinics into patient-centered medical homes in a large Hispanic community. Patient-centered medical homes included multidisciplinary care teams, patient education, electronic health records system with up-to-date patient information and, disease registry and were linked to other providers and community institutions. This led to a 9.2% decrease of mean visits per patient to the emergency department following implementation of the model (p = 0.001). During the same period, hospitalizations for the cohort dropped by 5.8% (p = 0.25) [43].

### Strategies for implementing culturally competent healthcare

**Promoting changes within the organization.** In order to improve healthcare for migrants, the “Migrant Friendly Hospital Initiative” in Geneva (Switzerland) decided to give to all new staff members a brief presentation of the initiative and about interpreter services during their mandatory orientation day. Aside from distributing brochures on the “Health for all Network”, migrant friendly services and information on the work with an interpreter, the hospital also organized numerous public events to raise awareness for its initiative. Hospital staff was significantly more likely to use the service of interpreters and other migrant friendly structures at the hospital. Overall, providers’ awareness increased and difficulties working with migrant patients decreased significantly [46]. The “Sick-Kids Cultural Competence Initiative” at the Hospital for Sick Children (Canada) established a Champion program in which cultural competence champions obtained advanced cultural competence education and became designated change agents and role models. Over 2,100 hospital staff members attended the workshops. Participants fulfilled 78% of the documented commitments to change and planed on realizing another 16% of commitments. Commitments to change were related to changes in practice, beliefs or attitudes and to continuing education related to culture and culturally competent care. Following a Cultural Competence Initiative promoting interpreter services, a significant increase in the use of face-to-face interpretation and a doubling of the number of minutes of telephone interpretation use was observed [76].

### Model of culturally competent healthcare provision

**Fig 2:**
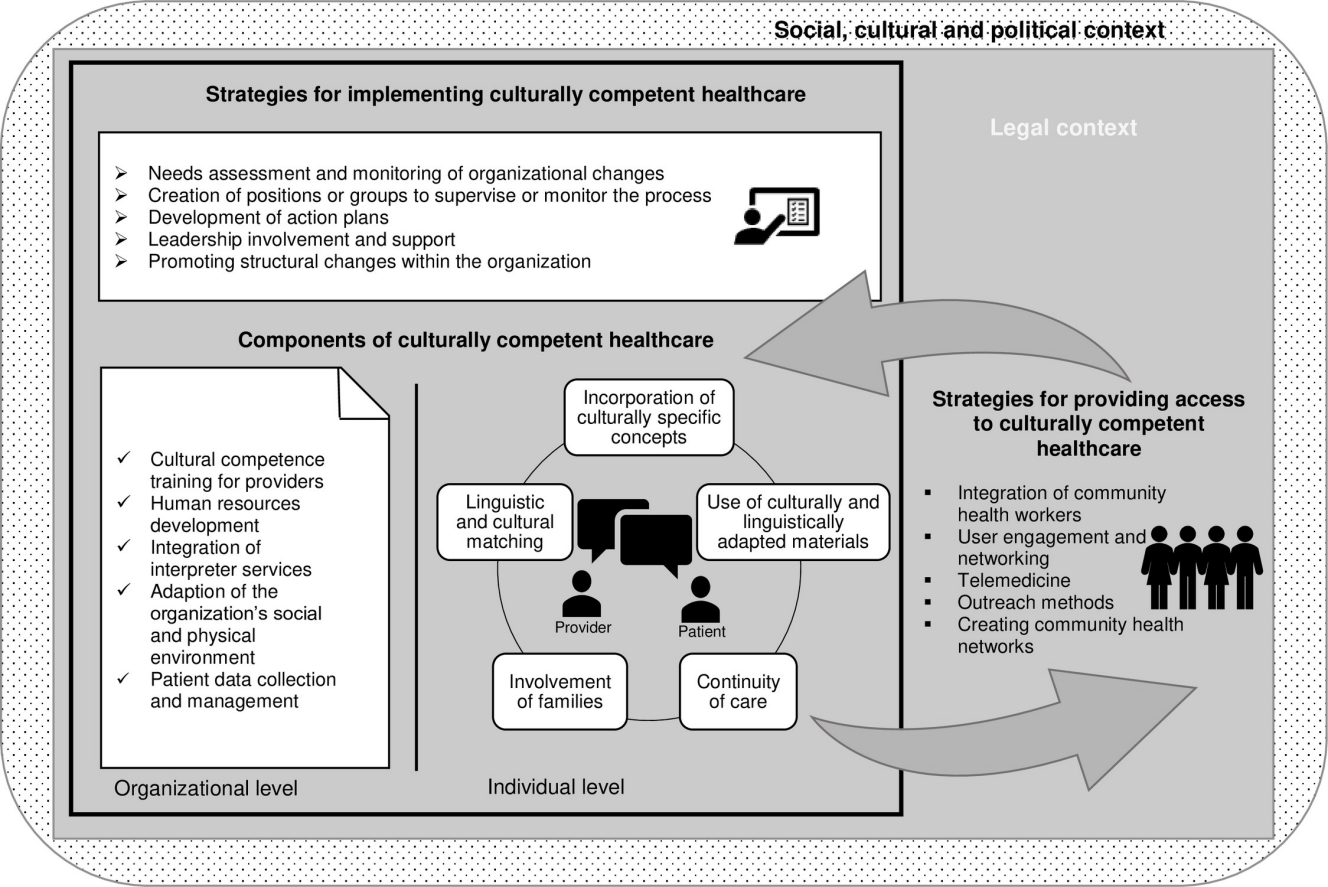
Model of culturally competent healthcare provision.

Extracted components and strategies were organized into a model, the “Model of culturally competent healthcare provision” (Fig 2). Importantly, the model is embedded in the legal context of a given country’s health system that regulates the organization of the system and the access to healthcare for individuals depending on their legal status in the country in question. Then again, the legal context is shaped by the political and social context of a country. In conclusion, the possibilities and usefulness of implementing identified strategies and components depend on the health system in which they are implemented as well as on the legal, social and political context of the country or region.

## Discussion

This review systematically searched for evaluated culturally competent healthcare interventions from which components and strategies for providing culturally competent healthcare to CLDP were collected and their effects on outcome measures were examined. Twenty categories of components were identified and clustered into four groups. A model integrating those interventions is proposed. Data on the effects of identified components and strategies were not available for all categories because in most studies a combination of multiple strategies and components was implemented simultaneously, and therefore statements about the effects of single categories were not possible. However, for fifteen categories qualitative and/or quantitative data were available, but synthesis of data was difficult because of the diversity of studies and outcome measures. In general, the effects of identified components and strategies were positive but often small or not significant compared to treatment as usual. Qualitative data suggest that these components and strategies were appreciated and found helpful by patients and providers. Furthermore, they confirmed many of the components and strategies proposed by existing conceptual models or frameworks of cultural competence [8–10, 21]. Existing models and reviews have already mentioned implementing the following strategies: providing care in different languages, recruiting bicultural/bilingual healthcare professionals, training healthcare staff in cultural competence, integrating community health workers, including individual patients’ families into care, adapting the environment by offering ethnic meals and, written material in different languages, collaborating with minority communities and monitoring of the organizational development. Nonetheless, this review identified strategies used to improve healthcare for CLDP which are not included in existing models. These are primarily related to improving access to culturally competent healthcare for CLDP: telemedicine, outreach methods and the creation of community health networks. In fact, it appears that these strategies address the socioeconomic differences often associated with culturally and linguistically diverse backgrounds, rather than the actual cultural backgrounds themselves (e.g., Outreach methods for Hispanic farmworkers). Some strategies are integrated in conceptual frameworks but were not often found in empirical studies. These include adapting policies and integrating traditional healers into care.

Reported evidence is limited in this review because the majority of studies implemented multiple components or used different strategies simultaneously, and outcomes could therefore not be attributed to one specific component or strategy. Other authors noted that the methodological quality of included studies is often insufficient to support the effectiveness of culturally competent interventions [9, 11, 12, 21, 22]. Truong’s systematic review of reviews on cultural competence outlined that most reviews only found weak evidence for improvements in patient outcomes and moderate improvement in provider outcomes and utilization rates [12]. Diaz et al. state in their scoping review that the main cultural competence interventions in 57 of 83 studies were declared beneficial for the primary outcome as well as for secondary outcomes in 13 studies. In 12 interventions no effects were observed compared with standard care [22]. The effectiveness of organizational system-level interventions was not confirmed because interventions were “context-specific, there were too few comparative studies and studies did not use the same outcome measures” [11]. In this review comparative studies were available but generally compared an innovative culturally competent health intervention to treatment as usual. This approach is problematic, because it gives no information on whether the health intervention, the culturally competent components or a combination of both can be determined to be effective. Anderson at al. (2003) stated in their review that no sufficient evidence was found to determine the effectiveness of workforce diversity, use of interpreter services, patient-provider matching, use of culturally and linguistically appropriate health education materials and culturally specific settings. In the present review, moderate effects on patient outcomes were found for patient-provider matching. A systematic review on race and racial concordance on patient-physician communication studies demonstrated that racial discordance is related to poorer communication [94]. Brach and Fraser (2000) highlighted that even though a relationship between communication, adherence and outcomes exists, it has not been demonstrated specifically for linguistic matching. They found some evidence that the provision of professional interpreter had positive effects on utilization and satisfaction and reduced disparities in healthcare [9], which could not be verified in this review. Integrating community health workers had again positive but modest effects on patient outcomes and utilization rates, which has also been confirmed by Brach and Fraser (2000).

The strength of this review lies in its overall approach. The use of a scoping review methodology with a systematic literature search allowed for a broad overview on studies implementing culturally competent health interventions in healthcare facilities. A bottom-up approach was used, and components and strategies have been extracted from practice instead of deriving interventions from theoretical concepts of cultural competence. In this way we created a model based on feasible and actually implemented interventions. Compared to existing models, this model summarizes a variety of strategies on different levels. The “Analytic framework used to evaluate the effectiveness of healthcare system interventions to increase cultural competence” by Anderson et al. (2003) included five strategies, while the conceptual model by Brach and Fraser (2000) identified nine major cultural competency techniques. Fung et al. (2012) proposed strategies in 24 subdomains organized in eight domains for implementing organizational cultural competence, but these only concentrated on the organizational level. This model provides 20 strategies on four different levels. In addition to strategies on the individual and organizational level, the model points out how change within healthcare organization can be implemented and how patients can better access culturally competent healthcare, which was not as thoroughly considered by previous models. In combination with the detailed description of the strategies and components in Table 2, this review provides researchers, facility leaders and policy or decision makers with a unique catalogue of feasible strategies aiming to battle healthcare disparities and enhance healthcare for all patients. Importantly, it highlights that health systems and facilities are integrated into specific social, cultural, legal and political contexts that affect one another and influence the possibilities of implementing chosen strategies.

Some limitations must be considered. We included in our search different groups of culturally and linguistically diverse patients, such as migrants including refugees and asylum seekers but also racial or ethnic groups and minorities. Obviously, these groups are very heterogeneous and their needs and perceived barriers to healthcare may differ substantially. Nevertheless, we chose to include all different groups to create a broad overview of generally possible strategies. When implementing strategies, their appropriateness for the specific target group must be considered. In addition, interventions needed to be located explicitly in a healthcare organization, otherwise they were excluded from the review. Notably, interventions for CLDP are often implemented in community institutions such as community centers, churches or schools, but the focus if this review was to identify strategies implemented in healthcare organizations. Only studies published in English or German were considered to be included in the review, which may have caused a selection bias, and some relevant studies in other languages may have been excluded.

The majority of studies (76%) were from the US, and almost all studies were from industrialized countries. The US hosts the largest number of international migrants in the world with approximately 53% of migrants from Latin America, 25% from Asia, 14% and from Europe [95]. This is also reflected in the targets groups of identified studies, of which 48% were designated for Hispanics or Latinos. Interestingly, only 9% of studies were from the European countries of the UK, the Netherlands and Switzerland, even though Germany, Spain, the UK and France accommodate the highest numbers of approximately 31.9 million non-European Union (EU) nationals in Europe [96]. The high number of studies from the US is understandable but it limits the generalizability of the results and possibly the transferability to other health systems and groups. Importantly, the developed model does not claim to be comprehensive or completed but rather serves as an empirical baseline that needs to be verified and further developed. In this context it would be an asset to identify connections between target groups, types of implemented strategies, the respective health systems’ organization and perhaps even the legal, social and political context in chosen countries.

Despite the limitations of this review it provides a unique overview and categorization of culturally competent healthcare provision. Unfortunately, the effectiveness of identified components and strategies could not be confirmed and was even often impossible to evaluate because either no control group was available or the chosen control group did not give any information on the effectiveness of the culturally competent components but rather on the health intervention in combination with culturally competent elements. This presents a challenge for future research on the effectiveness of cultural competence in healthcare. An option would be to simplify or reduce the number of implemented components and to choose more appropriate control groups. Another option would be to improve the research methods in order to be able to evaluate single components of complex interventions. Using qualitative study designs might help to better understand what strategies are helpful to overcome healthcare disparities why and for whom. It is essential to keep the heterogeneity among CLDP in mind and to carefully consider interactions between societal, cultural, health related and personal factors to explain and reduce healthcare disparities.

## Supporting information

S1 File Search terms. (DOCX)

S1 Table Screening criteria. (DOCX)

S2 Table Characteristics of healthcare interventions. (DOCX)

S3 Table Study characteristics and results. (DOCX)

S4 Table Identified culturally competent components/strategies and categorization. (DOCX)
