# Supplementary material for: Culturally competent healthcare – A scoping review of strategies implemented in healthcare organizations and a model of culturally competent healthcare provision
Source: PLoS One. 2019 Jul 30;14(7):e0219971. doi: 10.1371/journal.pone.0219971 (PMC6667133; doi:10.1371/journal.pone.0219971)
Supplement: S1 File — (DOCX) [file pone.0219971.s001.docx]

**S1 File. Search terms**

**Search terms PubMed**

**Outcome - 1**

"cultural competence"[ALL] OR "cultutal competences"[ALL] OR ("cultural"[ALL] AND "competence"[ALL]) OR ("cultural"[ALL] AND "competences"[ALL])

OR

"cultural competency"[Mesh] OR "cultural competency"[ALL] OR "cultural competencies"[Mesh] OR "cultural competencies"[ALL] OR ("cultural"[ALL] AND "competency"[ALL]) OR ("cultural"[ALL] AND "competencies"[ALL])

OR

"cross-cultural competence"[ALL] OR "cross-cultural competences"[ALL] OR ("cross-cultural"[ALL] AND "competence"[ALL]) OR ("cross-cultural"[ALL] AND "competences"[ALL])

OR

"cross-cultural competency"[ALL] OR "cross-cultural competencies"[Mesh] OR "cross-cultural competencies"[ALL] OR ("cross-cultural"[ALL] AND "competency"[ALL]) OR ("cross-cultural"[ALL] AND "competencies"[ALL])

OR

"transcultural competence"[ALL] OR "transcultural competences"[ALL] OR ("transcultural"[ALL] AND "competence"[ALL]) OR ("transcultural"[ALL] AND "competences"[ALL])

OR

"transcultural competency"[ALL] OR "transcultural competencies"[Mesh] OR "transcultural competencies"[ALL] OR ("transcultural"[ALL] AND "competency"[ALL]) OR ("transcultural"[ALL] AND "competencies"[ALL])

OR

"multicultural competence"[ALL] OR "multicultural competences"[ALL] OR ("multicultural"[ALL] AND "competence"[ALL]) OR ("multicultural"[ALL] AND "competences"[ALL])

OR

"multicultural competency"[ALL] OR "multicultural competencies"[Mesh] OR "multicultural competencies"[ALL] OR ("multicultural"[ALL] AND "competency"[ALL]) OR ("multicultural"[ALL] AND "competencies"[ALL])

OR

"intercultural competence"[ALL] OR "intercultural competences"[ALL] OR ("intercultural"[ALL] AND "competence"[ALL]) OR ("intercultural"[ALL] AND "competences"[ALL])

OR

"intercultural competency"[ALL] OR "intercultural competencies"[Mesh] OR "intercultural competencies"[ALL] OR ("intercultural"[ALL] AND "competency"[ALL]) OR ("intercultural"[ALL] AND "competencies"[ALL])

OR

"cultural sensitivity"[ALL] OR ("cultural"[ALL] AND "sensitivity"[ALL])

OR

"cultural safety"[ALL] OR ("cultural"[ALL] AND "safety"[ALL])

OR

"cultural security"[ALL] OR ("cultural"[ALL] AND "security"[ALL])

OR

"cultural literacy"[ALL] OR ("cultural"[ALL] AND "literacy"[ALL])

OR

"cultural awareness"[ALL] OR ("cultural"[ALL] AND "awareness"[ALL])

OR

"cultural respect"[ALL] OR ("cultural"[ALL] AND "respect"[ALL])

OR

"cultural framework"[ALL] OR ("cultural"[ALL] AND "framework"[ALL])

OR

"cultural congruence"[ALL] OR ("cultural"[ALL] AND "congruence"[ALL])

OR

"cultural capability"[ALL] OR ("cultural"[ALL] AND "capability"[ALL])

OR

"cross-cultural opening"[ALL] OR ("cross-cultural"[ALL] AND "opening"[ALL])

OR

"intercultural opening"[ALL] OR ("intercultural"[ALL] AND "opening"[ALL])

OR

"culturally competent"[ALL] OR ("culturally"[ALL] AND "competent"[ALL])

OR

"culturally sensitive"[ALL] OR ("culturally"[ALL] AND "sensitive"[ALL])

OR

"culturally safe"[ALL] OR ("culturally"[ALL] AND "safe"[ALL])

OR

"culturally aware"[ALL] OR ("culturally"[ALL] AND "aware"[ALL])

OR

"culturally congruent"[ALL] OR ("culturally"[ALL] AND "congruent"[ALL])

OR

"culturally appropriate"[ALL] OR ("culturally"[ALL] AND "appropriate"[ALL])

OR

"migrant-friendly"[ALL]

OR

"culturally competent care"[Mesh] OR "culturally competent care"[ALL] OR ("culturally"[ALL] AND "competent"[ALL] AND "care"[ALL])

OR

"culturally competent practice"[ALL] OR ("culturally"[ALL] AND "competent"[ALL] AND "practice"[ALL])

**AND Setting - 2**

"organization"[ALL] OR "organizations"[Mesh] OR "organisation"[ALL] OR "organisations"[ALL] OR "organizational"[ALL] OR "organisational"[ALL]

OR

"organizational level"[ALL] OR "organisational level"[ALL] OR ("organizational"[ALL] AND "level"[ALL]) OR ("organisational"[ALL] AND "level"[ALL])

OR

"meso level"[ALL] OR ("meso"[ALL] AND "level"[ALL])

OR

"health care system"[ALL] OR ("health"[ALL] AND "care"[ALL] AND "system"[ALL])

OR

"delivery of health care"[ALL] OR ("delivery"[ALL] AND "health"[ALL] AND "care"[ALL])

OR

"health care"[ALL] OR ("health"[ALL] AND "care"[ALL])

OR

"health care practice"[ALL] OR ("health"[ALL] AND "care"[ALL] AND "practice"[ALL])

OR

"hospital"[ALL] OR "hospitals"[Mesh]

OR

"institution"[ALL] OR "institutions"[ALL]

OR

"clinic"[ALL] OR "clinics"[ALL]

OR

"health service"[ALL] OR "health services"[Mesh] OR ("health"[ALL] AND "service"[ALL]) OR ("health"[ALL] AND "services"[ALL])

OR

"mental health service"[ALL] OR "mental health services"[Mesh] OR ("mental"[ALL] AND "health"[ALL] AND "service"[ALL]) OR ("mental"[ALL] AND "health"[ALL] AND "services"[ALL])

OR

"health facility"[ALL] OR "health facilities"[Mesh] OR ("health"[ALL] AND "facility"[ALL]) OR ("health"[ALL] AND "facilities"[ALL])

OR

"mental health facility"[ALL] OR "mental health facilities"[Mesh] OR ("mental"[ALL] AND "health"[ALL] AND "facility"[ALL]) OR ("mental"[ALL] AND "health"[ALL] AND "facilities"[ALL])

**AND Intervention - 3**

"standard"[ALL] OR "standards"[ALL]

OR

"strategy"[ALL] OR "strategies"[ALL]

OR

"measure"[ALL] OR "measures"[ALL]

OR

"changes"[ALL]

OR

"plan"[ALL] OR "plans"[ALL] OR "planning"[ALL]

OR

"development"[ALL] OR "developments"[ALL]

OR

"intervention"[ALL] OR "interventions"[ALL]

OR

"policy"[Mesh] OR "policy"[ALL] OR "policies"[ALL]

OR

"program"[ALL] OR "programs"[ALL] OR "programme"[ALL] OR "programmes"[ALL]

OR

"guideline"[Mesh] OR "guidelines"[ALL]

OR

"implementation"[ALL] OR "implementations"[ALL] OR "implementing"[ALL]

OR

"approach"[ALL] OR "approaches"[ALL]

OR

"action"[ALL] OR "actions"[ALL]

OR

"procedure"[ALL] OR "procedures"[ALL]

OR

"method"[ALL] OR "methods"[ALL]

**AND Population - 4**

(cultural* AND divers*) OR (linguistic* AND divers*) OR (racial* AND divers*)

OR

"linguistically diverse"[ALL] OR ("linguistically[ALL] AND "diverse"[ALL])

OR

"ethnically diverse"[ALL] OR ("ethnically[ALL] AND "diverse"[ALL])

OR

"racially diverse"[ALL] OR ("racially[ALL] AND "diverse"[ALL])

OR

"foreign born"[ALL]

OR

"migrant born"[ALL]

OR

"migration background"[ALL] OR "migrant background"[ALL] OR ("migration"[ALL] AND "background"[ALL]) OR ("migrant"[ALL] AND "background"[ALL])

OR

"Transients and Migrants"[Mesh] OR "Transients and Migrants"[ALL] OR "Transient and Migrant"[ALL] OR ("Transients"[ALL] AND "Migrants"[ALL]) OR ("Transient"[ALL] AND "Migrant"[ALL])

OR

"migrant"[ALL] OR "migrants"[ALL]

OR

"emigrants and immigrants"[Mesh] OR "emigrants and immigrants"[ALL] OR "emigrant and immigrant"[ALL] OR ("emigrants"[ALL] AND "immigrants"[ALL]) OR ("emigrant"[ALL] AND "immigrant"[ALL])

OR

"immigrant"[ALL] OR "immigrants"[ALL]

OR

"refugees"[Mesh] OR "refugees"[ALL] OR "refugee"[ALL]

OR

"asylum seeker"[ALL] OR "asylum seekers"[ALL] OR ("asylum"[ALL] AND "seeker"[ALL]) OR ("asylum"[ALL] AND "seekers"[ALL])

OR

"ethnicity"[ALL] OR "Ethnic Groups"[Mesh] OR "Ethnic Groups"[ALL] OR ("Ethnic" [ALL] AND "Groups"[ALL]) OR "Ethnic Group"[ALL] OR ("Ethnic" [ALL] AND "Group"[ALL])

OR

"minority"[ALL] OR "minorities"[ALL] OR "minority groups"[Mesh] OR "minority groups"[ALL] OR "minority group"[ALL] OR ("minority"[ALL] AND "groups"[ALL]) OR ("minority"[ALL] AND "group"[ALL])

OR

"race"[ALL] OR "races"[ALL] OR "racial groups"[ALL] OR "racial group"[ALL] OR ("racial"[ALL] AND "groups"[ALL]) OR ("racial"[ALL] AND "group"[ALL])

OR

"underserved population"[ALL] OR "underserved populations"[ALL] OR ("underserved"[ALL] AND "population"[ALL]) OR ("underserved"[ALL] AND "populations"[ALL])

OR

"illegal immigrant"[ALL] OR "illegal immigrants"[ALL] OR ("illegal"[ALL] AND "immigrant"[ALL]) OR ("illegal"[ALL] AND "immigrants"[ALL])

OR

"undocumented immigrants"[Mesh] OR "undocumented immigrants"[ALL] OR "undocumented immigrant"[ALL] OR ("undocumented"[ALL] AND "immigrants"[ALL]) OR ("undocumented"[ALL] AND "immigrant"[ALL])

OR

"allochtonous populations"[ALL] OR ("allochtonous"[ALL] AND "population"[ALL]) OR ("allochtonous"[ALL] AND "populations"[ALL])

**Search terms Web of Science**

**Outcome - 1**

"cultural competence" OR "cultutal competences" OR ("cultural" AND "competence") OR ("cultural" AND "competences")

OR

"cultural competency" OR "cultural competency" OR "cultural competencies" OR "cultural competencies" OR ("cultural" AND "competency") OR ("cultural" AND "competencies")

OR

"cross-cultural competence" OR "cross-cultural competences" OR ("cross-cultural" AND "competence") OR ("cross-cultural" AND "competences")

OR

"cross-cultural competency" OR "cross-cultural competencies" OR "cross-cultural competencies" OR ("cross-cultural" AND "competency") OR ("cross-cultural" AND "competencies")

OR

"transcultural competence" OR "transcultural competences" OR ("transcultural" AND "competence") OR ("transcultural" AND "competences")

OR

"transcultural competency" OR "transcultural competencies" OR "transcultural competencies" OR ("transcultural" AND "competency") OR ("transcultural" AND "competencies")

OR

"multicultural competence" OR "multicultural competences" OR ("multicultural" AND "competence") OR ("multicultural" AND "competences")

OR

"multicultural competency" OR "multicultural competencies" OR "multicultural competencies" OR ("multicultural" AND "competency") OR ("multicultural" AND "competencies")

OR

"intercultural competence" OR "intercultural competences" OR ("intercultural" AND "competence") OR ("intercultural" AND "competences")

OR

"intercultural competency" OR "intercultural competencies" OR "intercultural competencies" OR ("intercultural" AND "competency") OR ("intercultural" AND "competencies")

OR

"cultural sensitivity" OR ("cultural" AND "sensitivity")

OR

"cultural safety" OR ("cultural" AND "safety")

OR

"cultural security" OR ("cultural" AND "security")

OR

"cultural literacy" OR ("cultural" AND "literacy")

OR

"cultural awareness" OR ("cultural" AND "awareness")

OR

"cultural respect" OR ("cultural" AND "respect")

OR

"cultural framework" OR ("cultural" AND "framework")

OR

"cultural congruence" OR ("cultural" AND "congruence")

OR

"cultural capability" OR ("cultural" AND "capability")

OR

"cross-cultural opening" OR ("cross-cultural" AND "opening")

OR

"intercultural opening" OR ("intercultural" AND "opening")

OR

"culturally competent" OR ("culturally" AND "competent")

OR

"culturally sensitive" OR ("culturally" AND "sensitive")

OR

"culturally safe" OR ("culturally" AND "safe")

OR

"culturally aware" OR ("culturally" AND "aware")

OR

"culturally congruent" OR ("culturally" AND "congruent")

OR

"culturally appropriate" OR ("culturally" AND "appropriate")

OR

"migrant-friendly"

OR

"culturally competent care"[Mesh] OR "culturally competent care" OR ("culturally" AND "competent" AND "care")

OR

"culturally competent practice" OR ("culturally" AND "competent" AND "practice")

**AND Setting - 2**

"organization" OR "organizations" OR "organisation" OR "organisations" OR "organizational" OR "organisational"

OR

"organizational level" OR "organisational level" OR ("organizational" AND "level") OR ("organisational" AND "level")

OR

"meso level" OR ("meso" AND "level")

OR

"health care system" OR ("health" AND "care" AND "system")

OR

"delivery of health care" OR ("delivery" AND "health" AND "care")

OR

"health care" OR ("health" AND "care")

OR

"health care practice" OR ("health" AND "care" AND "practice")

OR

"hospital" OR "hospitals"

OR

"institution" OR "institutions"

OR

"clinic" OR "clinics"

OR

"health service" OR "health services"[Mesh] OR ("health" AND "service") OR ("health" AND "services")

OR

"mental health service" OR "mental health services"[Mesh] OR ("mental" AND "health" AND "service") OR ("mental" AND "health" AND "services")

OR

"health facility" OR "health facilities"[Mesh] OR ("health" AND "facility") OR ("health" AND "facilities")

OR

"mental health facility" OR "mental health facilities"[Mesh] OR ("mental" AND "health" AND "facility") OR ("mental" AND "health" AND "facilities")

**AND Intervention - 3**

"standard" OR "standards"

OR

"strategy" OR "strategies"

OR

"measure" OR "measures"

OR

"changes"

OR

"plan" OR "plans" OR "planning"

OR

"development" OR "developments"

OR

"intervention" OR "interventions"

OR

"policy" OR "policy" OR "policies"

OR

"program" OR "programs" OR "programme" OR "programmes"

OR

"guideline" OR "guidelines"

OR

"implementation" OR "implementations" OR "implementing"

OR

"approach" OR "approaches"

OR

"action" OR "actions"

OR

"procedure" OR "procedures"

OR

"method" OR "methods"

**AND Population - 4**

(cultural* AND divers*) OR (linguistic* AND divers*) OR (racial* AND divers*)

OR

"linguistically diverse" OR ("linguistically" AND "diverse")

OR

"ethnically diverse" OR ("ethnically" AND "diverse")

OR

"racially diverse" OR ("racially" AND "diverse")

OR

"foreign born"

OR

"migrant born"

OR

"migration background" OR "migrant background" OR ("migration" AND "background") OR ("migrant" AND "background")

OR

"migrant" OR "migrants"

OR

"immigrant" OR "immigrants"

OR

"refugees" OR "refugee"

OR

"asylum seeker" OR "asylum seekers" OR ("asylum" AND "seeker") OR ("asylum" AND "seekers")

OR

"ethnicity" OR "Ethnic Groups" OR "Ethnic Groups" OR ("Ethnic" AND "Groups") OR "Ethnic Group" OR ("Ethnic" AND "Group")

OR

"minority" OR "minorities" OR "minority groups" OR "minority groups" OR "minority group" OR ("minority" AND "groups") OR ("minority" AND "group")

OR

"race" OR "races" OR "racial groups" OR "racial group" OR ("racial" AND "groups") OR ("racial" AND "group")

OR

"underserved population" OR "underserved populations" OR ("underserved" AND "population") OR ("underserved" AND "populations")

OR

"illegal immigrant" OR "illegal immigrants" OR ("illegal" AND "immigrant") OR ("illegal" AND "immigrants")

OR

"undocumented immigrants" OR "undocumented immigrants" OR "undocumented immigrant" OR ("undocumented" AND "immigrants") OR ("undocumented" AND "immigrant")

OR

"allochtonous populations" OR ("allochtonous" AND "population") OR ("allochtonous" AND "populations")

**Search terms PsycInfo**

**Outcome - 1**

(cultural AND competence) OR (cultural AND competences)

OR

("cultural" AND "competency") OR ("cultural" AND "competencies")

OR

("cross-cultural" AND "competence") OR ("cross-cultural" AND "competences")

OR

("cross-cultural" AND "competency") OR ("cross-cultural" AND "competencies")

OR

("transcultural" AND "competence") OR ("transcultural" AND "competences")

OR

("transcultural" AND "competency") OR ("transcultural" AND "competencies")

OR

("multicultural" AND "competence") OR ("multicultural" AND "competences")

OR

("multicultural" AND "competency") OR ("multicultural" AND "competencies")

OR

("intercultural" AND "competence") OR ("intercultural" AND "competences")

OR

("intercultural" AND "competency") OR ("intercultural" AND "competencies")

OR

("cultural" AND "sensitivity")

OR

("cultural" AND "safety")

OR

("cultural" AND "security")

OR

("cultural" AND "literacy")

OR

("cultural" AND "awareness")

OR

("cultural" AND "respect")

OR

("cultural" AND "framework")

OR

("cultural" AND "congruence")

OR

("cultural" AND "capability")

OR

("cross-cultural" AND "opening")

OR

("intercultural" AND "opening")

OR

("culturally" AND "competent")

OR

("culturally" AND "sensitive")

OR

("culturally" AND "safe")

OR

("culturally" AND "aware")

OR

("culturally" AND "congruent")

OR

("culturally" AND "appropriate")

OR

("migrant-friendly")

OR

("culturally" AND "competent" AND "care")

OR

("culturally" AND "competent" AND "practice")

OR

exp Cultural Sensitivity/

**AND Setting - 2**

"organization" OR "organizations" OR "organisation" OR "organisations" OR "organizational" OR "organisational"

OR

("organizational" AND "level") OR ("organisational" AND "level")

OR

("meso" AND "level")

OR

("health" AND "care" AND "system")

OR

("delivery" AND "health" AND "care")

OR

("health" AND "care")

OR

("health" AND "care" AND "practice")

OR

"hospital" OR "hospitals"

OR

"institution" OR "institutions"

OR

"clinic" OR "clinics"

OR

("health" AND "service") OR ("health" AND "services")

OR

("mental" AND "health" AND "service") OR ("mental" AND "health" AND "services")

OR

("health" AND "facility") OR ("health" AND "facilities")

OR

("mental" AND "health" AND "facility") OR ("mental" AND "health" AND "facilities")

OR

exp PSYCHIATRIC HOSPITALS/ or exp HOSPITALS/

OR

exp Health Care Services/ or exp Mental Health Services/ or exp Community Mental Health Services/ or exp Community Services/

**AND Intervention - 3**

"standard" OR "standards"

OR

"strategy" OR "strategies"

OR

"measure" OR "measures"

OR

"changes"

OR

"plan" OR "plans" OR "planning"

OR

"development" OR "developments"

OR

"intervention" OR "interventions"

OR

"policy" OR "policy" OR "policies"

OR

"program" OR "programs" OR "programme" OR "programmes"

OR

"guideline" OR "guidelines"

OR

"implementation" OR "implementations" OR "implementing"

OR

"approach" OR "approaches"

OR

"action" OR "actions"

OR

"procedure" OR "procedures"

OR

"method" OR "methods"

OR

exp HEALTH CARE POLICY/

**AND Population - 4**

(cultural* AND divers*) OR (linguistic* AND divers*) OR (racial* AND divers*)

OR

("linguistically" AND "diverse")

OR

("ethnically" AND "diverse")

OR

("racially" AND "diverse")

OR

"foreign born"

OR

"migrant born"

OR

"migration background" OR "migrant background"

OR

"migrant" OR "migrants"

OR

"immigrant" OR "immigrants"

OR

"refugees" OR "refugee"

OR

("asylum" AND "seeker") OR ("asylum" AND "seekers")

OR

"ethnicity" OR ("Ethnic" AND "Groups") OR ("Ethnic" AND "Group")

OR

"minority" OR "minorities" OR ("minority" AND "groups") OR ("minority" AND "group")

OR

"race" OR "races" OR ("racial" AND "groups") OR ("racial" AND "group")

OR

("underserved" AND "population") OR ("underserved" AND "populations")

OR

("illegal" AND "immigrant") OR ("illegal" AND "immigrants")

OR

("undocumented" AND "immigrants") OR ("undocumented" AND "immigrant")

OR

("allochtonous" AND "population") OR ("allochtonous" AND "populations")
