# Supplementary material for: Culturally competent healthcare – A scoping review of strategies implemented in healthcare organizations and a model of culturally competent healthcare provision
Source: PLoS One. 2019 Jul 30;14(7):e0219971. doi: 10.1371/journal.pone.0219971 (PMC6667133; doi:10.1371/journal.pone.0219971)
Supplement: S1 Table — (DOCX) [file pone.0219971.s002.docx]

**S1 Table. Screening criteria**

|  | Inclusion criteria | Exclusion criteria |
| --- | --- | --- |
| **Population** | | |
| (1) | Migrants, refugees, linguistically and culturally diverse patients, ethnic minorities, Latinos/as, black African American, etc… | Culturally diverse patients AND LGBT patients |
| **Design** | | |
| (2) | - Primary data - N > 2. | - Reviews, Meta-analyses - Study protocols - Single-case-studies - Letter to the editor |
| **Content** | | |
| (3) | - Description of at least one intervention which aims at   - improving healthcare utilization of (2)   - OR healthcare provision for (2)   - OR healthcare treatment of (2)   - OR cultural competence in healthcare facilities - Interventions must be described - If necessary: Sets of interventions which were evaluated as a whole (they may also include cultural competence trainings for providers) | - Evaluation of the current degree/state of cultural competence in healthcare facilities or of providers/staff members - Cultural competence trainings for providers or staff members - Studies evaluating cultural competence surveys |
| **Method** | | |
| (4) | - Quantitative or qualitative evaluation of the intervention |  |
| **Context** | | |
| (5) | - Explicit description of implementation in   - Hospital   - OR Health/ Primary Care/ Treatment Center/Centre   - OR Clinic   - OR Site   - OR Trust   - OR Health facility   - OR Health organization/organisation | - Interventions implemented in schools, community centers, libraries, etc… - Participants were only recruited in healthcare facilities |
