# Supplementary material for: Culturally competent healthcare – A scoping review of strategies implemented in healthcare organizations and a model of culturally competent healthcare provision
Source: PLoS One. 2019 Jul 30;14(7):e0219971. doi: 10.1371/journal.pone.0219971 (PMC6667133; doi:10.1371/journal.pone.0219971)
Supplement: S2 Table — (DOCX) [file pone.0219971.s003.docx]

**S2 Table. Characteristics of healthcare interventions**

| **Authors** | **Country** | **Target group** | **Type of intervention** | **Goal** | **Components of the intervention** |
| --- | --- | --- | --- | --- | --- |
| Aggarwal, N. et al. (2015) | USA | Mixed | Use of Cultural Formulation Interview for the DSM-5 | Improve mental health assessments | - CFI in itself designed to be culturally sensitive |
| Alegría, M. et al. (2008) | USA | Minority populations | The Right Question Project-Mental Health (RQP-MH) is a patient self-reported activation and empowerment strategy in mental health care | Improve attention and retention in mental healthcare | - Incorporation of cultural components that could influence minority patients’ experiences when taking an active role in care (not specified) |
| Anand, K. J. S. et al.  (2015) | USA | Latino children | Multilevel Health Care Delivery Intervention at pediatric intensive care unit (PICU) | Reduce mortality rates in pediatric intensive care units of Latino children | - Education of health care professionals regarding culturally competent care - Recruitment efforts to increase the number of bilingual staff - Availability of 24-hour interpreter services in the ED and PICU - Translation of consent forms and educational materials for patients and families - Culturally sensitive end-of-life care discussions, with participation of palliative care services (not specified) - Outreach efforts and contacts with the Latino community to remove barriers to health care access - Help from the city government and local health department for preventive services |
| Armengol, C. G. (1999) | USA | Hispanics | Multimodal culturally sensitive and neuropsychologically informed support group addressing barriers to emotional, social, and vocational adjustment among high-level functioning Hispanic/Latino traumatic brain injury survivors | Improve support for TBI survivors | - Bicultural and bilingual provider - Incorporation of culturally specific themes (acculturation, stressors of the migratory experience - Incorporation of attitudes and belief about disability, health care and support networks - Use of dichos - Patterns of interaction common among less acculturated Hispanics/Latinos were followed - Integration of Hispanic values |
| Aviera, A.  (1996) | USA | Hispanics/  Latinos | “Dichos" Therapy Group: a therapeutic use of Spanish language proverbs with hospitalized Spanish-speaking psychiatric patients | Improve treatment acceptability | - Bilingual provider - Use of Dichos to promote „cultural ambiance“ and help to deal with resistance and impasse |
| Barrio, C. et al.  (2010) | USA | Mexican Americans | Culturally based family intervention for Spanish-speaking Latino families with a relative diagnosed with schizophrenia | Improve treatment for people with schizophrenia | - Integration of familism, spirituality and religiousness, nonjudgmental cultural attributions, biculturalism, and cross- border living and resources |
| Bekaert, S  (2000) | UK | Ethnic minority group | Minority integration and healthcare provision a hospital setting | Improve healthcare for ethnic minorities | - Formation of a multicultural consultation group planning and implementing interventions - Design of 5 year plan to lead to standardization of health care for ethnic minority groups - Invitation of the joint commissioning development manager for ethnic minorities in Oxfordshire to guide practice - Identification of staff’s needs (informal survey) - Record keeping and monitoring of episodes of contact with people from minority groups - Reference packs, containing general guidance (e.g. regarding diet, language, name, religious groups, birth, death, concepts of illness) - Identification of the main language groups of the area (survey) - Language and advocacy Services were made available - Provision of ethnic menus - Complaints procedure made available in all languages |
| Bender, M. et al. (2013) | USA | Mexicans | „Vida Saludable“: A culturally appropriate intervention to improve health behaviors in Hispanic mother-child dyads | Improve prevention of obesity in mother-child dyads | - Trained (by investigators) bilingual promotora - Culturally adapted instrument measuring beverage consumption - Lessons were tailored, including visual - images of typical Hispanic families - Cooking class with cultural foods - study materials were forward-translated into Spanish - Materials and curriculum were designed with special attention to culture, low literacy (less than 3rd grade) - Cultural adaptation based on stakeholders’ input, focus group feedback |
| Beune, E. et al.  (2014) | Nether-lands | Ghanaians and African-Surinamese | Patient education intervention on blood  pressure (BP) and treatment adherence for patient of African origin with uncontrolled hypertension | Improve education for people with uncontrolled hypertension | - Incorporating culturally-specific aspects of patients' perceptions (based on Arthur Kleinman) - culturally appropriate written educational materials (specific languages, customs, habits, norms and dietary cultures) - referrals to neighborhood facilities that support patients in adopting healthier lifestyles and are suitable for Surinamese and Ghanaian people - Nurse practitioner (NP) trained in - specific knowledge of hypertension in the Ghanaian / Surinamese communities and general cross-cultural counselling techniques |
| Carrillo, J. E. et al.  (2011) | USA | Hispanics | A regional health collaborative | Improve healthcare for a Hispanic communities | - Four multiyear strategies: - Establishing patient-centered medical homes - Transforming clinics into medical homes - Medical home designation - Related Goals: building a workforce that could address the linguistic, cultural, and health literacy needs of patients (set up an Office of Care Management, improving access though a centralized contact center for information and appointment scheduling, improve cultural competency by employing bilingual and bicultural community health workers and “navigators” of the health system) - Information technology solutions have included the development of a personal health record for each patient, patient-specific disease dashboards, and a population-based disease registry. - Implementing a targeted care intervention: the targeted care intervention focused on the critical hospital-to-home transition period to minimize preventable readmissions - creating a “medical village”: (geographically defined community with a number of patient-centers medical homes linked to other providers and community-based resources) - collaboration with the New York City and State health departments to help local physician practices adopt electronic health record systems, transform themselves into patient-centered medical homes, and establish health information exchanges. |
| Chow, W. et al.  (2011) | Canada and Japan | Culturally diverse communities | Adapted assertive community treatment (ACT) model in Mount Sinai Hospital and KUINA Center | Improve healthcare for culturally diverse communities | - Inclusion of bilingual and bicultural staff - Assignment of clinicians to patients on the basis of cultural and language requirements - Incorporation of cultural and ethnic elements into assessment and treatment plans (e.g. traditional Chinese medicines) - Promoting culturally appropriate supportive housing - Incorporating families in clients’ rehabilitation plans - Delivering multifamily psychoeducational groups - Prioritizing and incorporating cultural formulation and clients’ explanatory models - Having a general practitioner who is also a psychotherapist on the team to enhance assessment, management, and follow- up of clients’ general medical and counseling needs - Staff training - Exchange with KUINA Center in Japan |
| Cooper, L. A. et al. (2011) | USA | Mixed | Patient-centered care and hypertension control in underserved primary care patients | Improve care for patients with hypertension | - Trained community health workers (CHWs) administered the intervention - Physician communication skills training: Skills relevant to increasing patient engagement, activation - All patients received a monthly health - education newsletter designed to meet the needs of low literate adult readers |
| Cooper, L. A. et al. (2013) | USA | African Americans | Patient-centered collaborative care interventions for depression among African Americans in primary care settings | Improve depression treatment for African Americans | - initial needs assessment (Standard assessment + questions on access barriers, attribution of illness, use of spirituality and concerns about treatment; social stressors and communication problems with health professionals) - Contact information for culturally sensitive psychotherapists as appropriate one-on-one telephone follow-ups by a Depression case manager (African American woman) - Educational material: culturally targeted materials designed to address barriers to depression treatment |
| Coronado, G. D. et al. (2011) | USA | Hispanics | Clinic-based colorectal  cancer screening promotion program | Improve prevention of colorectal cancer | - Providers were Spanish-speaking - Mailed packet contained materials in English and Spanish - Telephone reminders - Educational home visit by health promoter (community members) and a medical assistant - All project materials were developed for a low literacy audience |
| Culica, D. et al.  (2008) | USA | Hispanics | Community Health Worker as Sole Diabetes Educator (CoDE) | Improve healthcare education for patients with diabetes | - Bilingual community health workers - Individual education visits, which addressed recommended diabetes knowledge and self-management skills - assessment and case management visits |
| Dahhan, N. et al. (2012) | Nether-  lands | Ethnic minorities | The Mosaic Outpatient Clinic (MOC) | Improve healthcare of chronic sick ethnic minority children | - Patient centered consultation and exploration of culturally sensitive issues in the health care process - Mediators serve as the interface, translating language and interpreting culture, between the health care provider and the ethnic minority patient - Data collection of complete medical history, of demography, family situation, concepts, problems and experiences about healthcare and disease of parents - Development of parents’ profiles - Development of individual treatment plan (e.g. follow-up consultations, referrals) - Plan was discussed with patients and parents by the healthcare worker and supervisor together |
| Delphin-Rittmon, M. E. et al.  (2016) | USA | Ethnically diverse community | Bilevel cultural competence intervention | Improve mental healthcare for ethnically diverse patients | - Development of a bilevel training intervention for providers in cooperation with persons in recovery (PIR) with diverse ethnic backgrounds - Cultural competence training for staff members - Implementation of an ongoing cultural competence committee - Organizational level cultural competence assessment - Development of a cultural competence plan in consultation with the agency leadership, faculty and PIR that matched the agency’s capacity for implementation |
| Doorenbos, A. et al. (2011) | USA | Native Americans | Calendar mail-out with cancer related health-message | Improve cancer screening rates | - Native art each month matching cancer health-related message |
| Edwards, G. et al.  (2011) | United Arab Emirates | Culturally diverse mothers | WHO/Unicef Babyfriendly Hospital initiative in to increase breastfeeding rates | Improve breastfeeding rates after birth in a hospital | - Establishment of a steering group committed to the ethos of BFHI - Assessment of barriers to and knowledge on breastfeeding in hospital - Formulation of a 10 steps plan - Policies and procedures to support the changes in practice were developed - Development of an education program for all staff - Development of an antenatal BFHI educational program for women and their families - Invitation to talk to a breastfeeding advocate about the 10 steps, demonstration of “how-to” - Promotion of rooming in, skin-to-skin contact and early feeding - Staff breastfeeding course / workshop (mandatory, 20 hours) - A set of booklets in different languages was produced - Flyer and DVD were made for women who may have trouble reading - A lactation clinic was set up - Mother to mother groups set up |
| Ferdinand, L.A.  (2009) | USA | Culturally diverse patient | Patient-Centered Culturally Sensitive Health Care (PC-CSHC) Intervention Program | Improve healthcare for culturally diverse patients | - Changing the physical health care clinic environment and clinic policies (culturally sensitive calendar, educational brochures, magazines, comment cards, bilingual restroom signs, posters, art featuring people from different cultures, bilingual policy brochures, displaying toys for patients’ children, DVDs and videos on health topics shown in the waiting area) - Training health care providers and office staff to engage in culturally sensitive behaviors and attitudes - Patient Empowerment Training (overview of health disparities problem, skills training, small group discussion on how the skills-training components could be used in practice, questions to provider panel about their healthcare or the healthcare system) |
| Galvin, S. et al.  (2008) | USA | Cambodian women | Project HELP (Hospital Education in Lactation Practices): Intervention to increase breastfeeding initiation among Cambodian women | Improve number of breastfeeding initiation among Cambodian women | - Creation of a Cambodian menu:   - Staff visit of the Cambodian market to consider nutritional content and the financial feasibility of purchasing foods   - The Cambodian interpreter cooked traditional recipes in the hospital kitchen, to educate the hospital chef, the director of food and nutrition, and hospital dietitians - The Cambodian menu was made available to all postpartum women |
| Garvin C.C. et al.  (2004) | USA | Culturally diverse patients | A community based approach to diabetes control | Improve healthcare for culturally diverse patients with diabetes | - Support Groups: Contracting agencies established regularly scheduled support group meetings for diabetes patients and their support network. These group meetings have been tailored to meet the cultural needs of each of the 3 broad racial/ethnic groups provided in the patients’ primary language - Peer Education: One full time equivalent peer educator per racial/ethnic community facilitates support groups, assists in education and self- management classes, and arranges for the dissemination of diabetes materials in community settings. - Education Classes: topics were dealt with in a culturally relevant manner (focus on community concerns, cultural barriers, that arise when people try to make changes) - Self-Management classes: These classes assist diabetes patients in increasing their confidence in their ability to manage their disease - Enhanced Use of a Diabetes Registry: Software used to create diabetes registries within each of the participating community clinics, so that individuals who have not received appropriate services can be identified and targeted for interventions. - Case Coordination for Appropriate Patients using the Diabetes registry to improve and monitor patients’ care |
| Gary, T. L. et al.  (2009) | USA | African American | Nurse case manager  and a community health worker team to improve care for patient with diabetes Mellitus Type 2 | Improve healthcare for patients with diabetes Mellitus Type 2 | - Telephone calls to remind participants about preventive health screenings - A written summary of their health care utilization was sent to the participant’s primary care provider - Participants received DM-specific information in the mail - individualized, culturally tailored care provided by a nurse case manager (NCM) and a community health worker (CHW) (not specified) - development of culturally tailored intervention action plans (IAPs) developed to address traditional cardiovascular risk factors and nontraditional obstacles to optimal DM care and self-management |
| Gerrish, K. et al.  (2004) | UK | Overseas nurses | Adaptation program for overseas Registered nurses | To improve overseas recruitment | - Period of supervised practice together with taught input - minimum of 10 weeks of supervised practice in a designated clinical area - Each overseas nurse was supported by one or more registered nurse mentors |
| Gil, S. et al.  (2016) | USA | Patients and families with limited English proficiency | The Limited English Proficiency (LEP) Patient Family Advocate Role | Improve pediatric care in oncology settings | - The LEP Patient Family Advocate role was created with the aim of improving access, promoting effective communication, and equalizing care for children with cancer from families with LEP. - Role 1: Specialized Medical Interpreter - Role 2: Cultural Liaison: The LEP advocate provides expertise and guidance to the health care team in the target-language’s culture(s), and to the family in the dominant U.S. culture and the culture of Western medicine. - Role 3: Healthcare Systems Advocate/Adjunct Case Management: The LEP advocate is readily available to assist the health care team with internal and external coordination across the continuum of care. |
| Gilmer, T. P. et al.  (2005) | USA | Ethnic minorities | Project Dulce, a combined stepped-care diabetes nurse case management program and culturally oriented peer-led self-empowerment training program | Improve healthcare for patients with diabetes and reduce costs | - One-on-one visits: Initial visit with nurse and additional visits with nurse and dietitian - Telephone contact is used for appointment reminders and to answer specific questions - Group self-management training program: - curriculum delivered by trained peer educators (or promotoras) who are from the patient population, have diabetes themselves, and are of the same cultural/ethnic group as the participants. - Classes are taught in the patients’ native language |
| Goncalves, M. et al.  (2013) | USA | Portuguese speaking patients | The Portuguese Mental Health Program (PMHP) | Improve mental health care for Portuguese speaking patients | - Bilingual and bicultural staff to serve children and adults from Brazil, Portugal, and Cape Verde - 95% of the providers speak Portuguese |
| Halcon, L. L. et al.  (2010) | USA | Ethiopian and Somali refugee women | Health Realization,  a community-delivered, psychoeducational,  mental health intervention that focuses on resilience | Improve mental health support for women refugees | - Culturally adapted and translated educational materials including visual cues for participants who did not read their native written language - socializing and sharing an East African dinner - intervention in English with concurrent translation - 15-minute prayer break was held promptly at sunset during each session - use of ethnographic methods as culturally appropriate ways to explain the concepts through traditional stories, pictures, proverbs, and faith-based metaphors and teachings |
| Hamilton, L. J. et al.  (2013) | USA | Ethnically diverse | Pediatric Medical Home Program: primary care model focused on providing intensive care coordination for medically complex, ethnically diverse children with special health care needs | Improve care for medically complex, ethnically diverse children with special health care needs | - Designated full-time, bilingual family liaison who acts as a bilingual healthcare system navigator, does triage of parents’ questions and concerns, ensures that the care plan is implemented, obtains outside medical records and insurance authorizations, forms relationships and interacts with community agencies - General pediatrician who develops, maintains, and manages a written care plan for each patient - Patients receive an ‘‘All About Me’’ Binder, which includes a problem list, care plan, medication list, and physician contact information, which is updated regularly - Family involvement in the development of the care plan and the program |
| Hatcher, S. et al.  (2016) | New Zealand | Maori | Culturally informed treatment in Maori who present to hospital after self-harm | Improve measures of care after an episode of self-harm for Maori patients | - Therapists are Maori. - the process of therapy explicitly incorporates Maori cultural beliefs and values which we chose to describe using a powhiri (welcoming ceremony) model - Cultural assessment: establishing where both the therapist and the patient belong and what connections they have - Patient support for up to 2 weeks: 1-2 face-to-face or telephone sessions - Postcard contact for one year: Eight postcards were sent containing a short message offering support - Improved access to primary care: encouraging participants to attend their GP for a physical health check paying particular attention to cardiovascular risk factors especially alcohol and smoking - Problem solving therapy (PST) |
| Hudelson, P. et al.  (2014) | Switzer-land | Migrants | Migrant Friendly Hospital’’ Initiative | To improve healthcare for migrants | - Creation an interdepartmental and interprofessional working group (‘‘Health for All Network’’) - Creation of a reference-nurse post at the hospital for migrant care issues - Inclusion of patient language data in the electronic patient file, in order to facilitate timely identification of patients requiring interpreter services - Promotion of a national telephone interpreting service in 4 emergency services at the HUG, where access to face-to-face interpreters is rare - Brief presentation to all new staff during an obligatory staff orientation day about interpreter services and other ‘‘migrant friendly’’ services at the HUG - Development and dissemination of brochures containing information about the ‘‘Health For All Network’’; migrant friendly services at the HUG and when and how to work with an interpreter - Organization of a number of public events to bring attention to the Health For All Network and its activities |
| Ivey, S.L. et al.  (2012) | USA | Chinese American | Culturally and linguistically competent  health coach intervention for Chinese-American  patients with Diabetes | Improve healthcare for Chinese-American patients with diabetes | - All health coaches, the dietitian, and most physicians were ethnically and linguistically matched to their Chinese patients. - Pre-visit meeting: health coach meets with patient to complete routine assessments, data gathering, medication reconciliation, determination of patient agenda - Data entry: health coach enters data into chronic disease management database - Physician visit: Physician uses summary of patient’s current and historical data from the database - Post-visit meeting: health coach meets with patient to ensure that patient understands the care plan, assist patient in scheduling follow-up appointments, navigating the referral system - 3-month follow-up visit is scheduled with the primary care physician - Health coach makes follow-up phone call to patient - Diabetes education visit with registered dietitian - Recommendations took into account cultural implications - Physicians who did not speak their patient’s language would communicate with their patient through a clinic interpreter. |
| Kalister, H. et al.  (1999) | USA | Immigrant families | Pharmacy-Based Treatment of Minor Illnesses | To improve access to nonprescription medicines for home treatment of minor childhood illnesses | - Pharmacists are trained to evaluate and to treat children and adolescents aged with minor acute illnesses - Development of protocols and encounter forms for pharmacist evaluation of 5 pediatric condition - Development of educational materials in English for each condition. The English pamphlets were then translated and published in 8 bilingual formats. |
| Kanter, J. et al.  (2010) | USA | Latinas | Culturally Adapted Behavioral Activation (BAL) for Latinas with depression | Improve engagement and retention in mental health treatment | - Bilingual provider - BAL manual was culturally adapted - the BAL manual listed free, low-cost, and culturally sensitive activation homework assignments - attention was paid to cultural values such as *familísmo, personalismo*, *marianismo*, and *machísmo* and how they influence activation - key BAL terms and therapy materials such as activity monitoring forms were translated into Spanish |
| Karmali, K. et al.  (2011) | Canada | Culturally diverse children and their families | Cultural Competence Initiative at the Hospital for Sick Children (SickKids) | Improve healthcare for culturally diverse children | - Establishment of the New Immigrant Support Network (NISN) to improve access to quality healthcare and partnered with multiple stakeholders, departments and worked closely with the senior management team - Cultural competence education to healthcare providers and managers - Translation of patient education materials in up to nine languages - Needs-assessment (organizational and provider level) - Cc learning modules were individually tailored for different staff groups - Placement and implementation of 12 kiosks that provide in multiple languages to help patients and families find their way around the hospital - Patient satisfaction survey was translated into seven languages to determine how effectively the hospital meets the needs of its population - Promotion of the use of face-to-face and telephone interpretation services - Establishment of a Champions Program: champions received advanced education in cultural competence and acted as change agents and role models - Allowing protected time for staff from different departments to attend the cultural competence education and training |
| Kim, J. et al.  (2015) | Canada | First Nations (aboriginal Canadians) commun-ities | Teleopthalmology service delivery model | Improve access to teleopthalmology care for First Nations communities | - First Nations technicians and a trained teleopthalmology eye care nurse travelled to the remote First Nations communities in a truck that was dedicated to the teleopthalmology project - Adherence to First Nations’ cultural values as most important aspects of the project - Involvement of First Nations communities from the beginning - Exchange with the British Columbia Association of Optometrist - Naming of potential patients by participating First Nations communities - Phone calls from the ITHA team to schedule individual screening appointments during clinics to be held in their communities - All clients were contacted after their appointments and were mailed copy of their report - Development of a clinical procedure and protocol manual - Specialized one-on-one training for team members in teleophthalmology - Distribution of educational material in the waiting area, small MP3 players with which to listen to diabetes education content while awaiting their appointments - Capacity building opportunity to three First Nations youth and continued education |
| Kline, K. N. et al.  (2016) | USA | Hispanics | SHL-program (Sugar, Heart, and Life), a culturally sensitive entertainment-education telenovela for patients with diabetes mellitus | Improve education on diabetes mellitus | - Culturally sensitive narratives through focus groups (72 people) - English and Spanish versions - culturally sensitive characters and situations (Example of Hispanic families) - Special design considerations were made, including minimal use of on-screen text, narration by professional voiceover talent, and user-friendly navigation |
| Kurth, A. E. et al.  (2016) | USA | Hispanics / Latinos | Linguistic and cultural adaptation of a computer-based counseling program (CARE+ Spanish) | Improve HIV treatment adherence and risk reduction for people living with HIV/AIDS | - Intervention was delivered in Spanish - Cultural equivalence was assured through forward-back translation method and feedback from target group |
| La Roche, M. J. et al.  (2011) | USA | Latinos | A Culturally Competent Relaxation Intervention for Latino/as (CCRI) with anxiety | Improve treatment adherence and effectivity in treatment for anxiety | - Bilingual providers - Inclusion of allocentric (the tendency to define oneself in relationship to others) relaxation interventions, considered to be more appropriate for Latinos |
| Levin-Zamir, D. et al.  (2011) | Israel | Ethiopian refugees | Refuah Shlema: a cross-cultural program for promoting communication and health among Ethiopian immigrants | Improving health for Ethiopian immigrants | - Integrating Ethiopian immigrant liaisons in primary care as intercultural mediators - In-service training of clinical staff to increase cultural awareness and sensitivity - Health education community activities |
| Mauldon, M. et al.  (2006) | USA | Latinos / Hispanics | Tomando Control, a culturally appropriate diabetes education program for Spanish-speaking individuals with diabetes mellitus | Improve education on diabetes mellitus | - Bilingual providers - Handouts and 2 nutritional guides specific to Latino diets (written at the fifth-to seventh-grade level) - Provision of culturally adapted and nutritionally sound meals - Use of written materials was minimized, and demonstration/return demonstration of skills was emphasized |
| McMurray, J. et al.  (2014) | Canada | Refugees | Government assisted refugees (GARs) health clinic | Improve healthcare for refugees | - Partnership between a health clinic for GARs, local reception centre and community providers - Gateway services are provided by Reception House case workers and trained professionals - Comprehensive care is delivered by family physicians at the refugee health clinic - Language supports (interpreters) funded by Reception House - Ancillary services are delivered in the community by a variety of providers - Establishment of protocols and guidelines for diagnosis and management of diseases - Initial intake assessments performed onsite at Reception House within days of GARs’ arrival - international medical graduates in training whose input has enhanced the cultural sensitivity and competence of clinic processes |
| Mehler, P. S. et al.  (2004) | USA | Russian patients | Language and cultural concordance in Russian patients with diabetes | Improve healthcare for patients with diabetes | - Arrival of a bilingual Russian internist trained in USA and Russia |
| Melkus, G. D. et al.  (2004) | USA | Blacks | Culturally competent intervention of education and care for Black women with Type 2 Diabetes | Improve healthcare for Black patients with diabetes | - Cognitive-behavioral intervention program - Two of the four nurses and the lay health - assistant were black American women - Presentation and discussion were the primary methods used for each session - Written materials and videotapes using illustrations and video characters of black Americans - Culturally specific recipes were incorporated and used as handouts - Feedback on material through focus groups and from the community advisory board of local black leaders and community members |
| Menon, U. et al. (2008) | USA | Mixed | Interactive, computer based culturally sensitive education on colorectal cancer screening | Improve colorectal cancer screening rates | - Culturally adaptation of the intervention based on focus group discussion with representatives of target population (not specified) |
| Moreno, F.A. et al. (2012) | USA | Hispanics | Standard webcam telepsychiatry treatment of depression | Improve access to culturally competent care | - Bilingual psychiatrists |
| Munoz, R. F. et al.  (2007) | USA | Latinas | Mamás y Bebés/Mothers and Babies Course: Health promotion group for prevention postpartum depression for Low-income Latinas | Improve prevention of postpartum depression | - Bilingual providers - Reinforcing values such as collectivism and familismo - Validating Latinas’ values and beliefs regarding pregnancy, childrearing practices, and motherhood - Addressing Latinas’ attitudes toward mental illness and seeking mental health services - participant course manual was written and adapted considering the sample’s education level and intra-group cultural, racial, and linguistic differences - Adhering to common cultural verbal and nonverbal communication norms - Validating the role of religion and spirituality in the health and healing of Latinas - Allowing to relate frustrations and painful experiences of discrimination and racism - Expanding Latinas’ knowledge without devaluing their cultural beliefs |
| Nowalk, M. P. et al.  (2008) | USA | Culturally diverse adults | Individualized, culturally appropriate interventions to raise adult vaccination rates | To improve vaccination rates of culturally diverse patients | - Educational sessions for all clinical staff - Immunization posters throughout the health center (also posters in Vietnamese and Spanish) and vaccination poster competition - Immunization videos in the waiting room - Reminders to all eligible adults (review of patient’s chart) - Created a provider prompt for immunizations in its electronic medical record - Center held four influenza vaccination clinics in the surrounding neighborhood - Immunization quiz for clinical and clerical staff - Contest for the most prolific vaccinator - Vaccinators and vaccinees received a small treat at the time of vaccination |
| Ohr, S.O. et al.  (2016) | Australia | Overseas nurses | The transition of overseas qualified nurses and midwives into the Australian healthcare workforce | To improve overseas recruitment | - The Overseas Staff Support Program aims to improve the experience of the overseas qualified staff - Support prior to arrival, on arrival and on commencement of their work |
| Oppong, B. et al.  (2016) | USA | Minority women | Culturally sensitive patient care paradigm | Improve the process of cancer screening for patients at risk of breast cancer | - Women who require additional workup after screening mammogram are assigned a patient navigator, who faciliates the diagnostic evaluation with further imaging or biopsies - There are two navigators and they reflect the population serviced by CBCC, Black and Hispanic with one being a fluent Spanish speaker |
| Ortega, A. N. et al.  (2002) | USA | Hispanics | Access to Community Care and Effective Services and Supports Program for homeless persons with severe mental illness | Improve access to mental healthcare for homeless people | - Rely on principles of assertive community treatment (ACT) - Client-clinician ethnic and language matching |
| O'Shaughnessy, R. et al.  (2012) | UK | West African women | Sweet mother, mental health promotion group for West African women and their babies | Improve prevention of mental health problems of mothers and infants | - Culturally appropriate food - Providers are culturally trained |
| Poureslami, I. et al.  (2016) | Canada | Punjabi and Chinese people | Video Education/self-management regarding inhaler use for Punjabi and Chinese patients | Improve education on health behavior (inhaler use) of people with asthma | - Development of intervention materials using a community based participatory research approach, actively involving Punjabi and Chinese subjects - Translation of videos and all materials in 3 languages (Mandarin, Cantonese or Punjabi) - Cultural beliefs and practices from the 3 target communities were applied - Educators/Providers from target communities |
| Reavy, K. et al. (2012) | USA | Non-English speaking refugees | C.A.R.E. (Culturally Appropriate Resources and Education): a clinic model for refugee health care | Improve healthcare for refugees | - Needs-assessment in focus groups with women from the refugee communities - Role of health advisor was created - Integration of certified medical interpreter - Patient group appointments for prenatal care - Patients appointment for pediatric well-baby care - Languages spoken include Arabic, Burmese, Dari, Farsi, French, Karen, Kirundi, Lingala, Nepali, Pashtu, Russian, Somali, Swahili, and Uzbek (On average, 5 different non-English languages are spoken at each clinic) - Decoration with art from refugees’ native countries - Scheduling of taxi transportation - Training of interested bilingual adults to take the medical interpretation certification test |
| Redwood, D.G. et al.  (2016) | USA | Alaska Natives | Alaska Native Colorectal Cancer (CRC) Family Outreach Program | Improve colorectal cancer screening rates | - CRC cases are ascertained annually from the Alaska Native Tumor Registry - A CRC screening patient navigator uses the information to provide direct outreach to Alaska Native family members encouraging them to get screened for CRC using telephone and mailed reminders, scheduling them into the screening clinic, and guiding them through the cancer screening process - First-degree relative lists are also sent annually to regional Tribal Clinical Directors for use by their facilities in identifying patients at increased risk of CRC. - Asking to CRC patients for a contact list of their first-degree relatives. |
| Riggs, E. et al.  (2017) | Australia | Refugee women from Burma | Culturally safe group pregnancy care | Improve pregnancy care among refugee women from Burma | - Women had individual antenatal appointments (according to the hospital schedule) with a „caseload” midwife and either a telephone or on-site professional interpreter - Group information sessions: co-facilitated by the midwife, bicultural worker, and maternal and child health nurse. |
| Rodrigue, J R. et al.  (2008) | USA | Blacks | Home-based education approach to increase Iive donor kidney transplantation | To improve numbers of livedonor kidney transplantation | - The transplant surgeon and/or nephrologist talked to the patients about LDKT during a routine clinic visit - Patients attended a nurse-led education session with other transplant patients. Family members were also invited. - Patients also received written materials about LDKT and living donations - Patients received a home visit (interactive; e.g. “roundable” format) by transplant health educators, who met with the patient (and his/her invited guest; family members, friends, co-workers) to discuss LDKT and living donor donation. - Patients watched a brief 15-minute videotape on living kidney donation during home visit and also received written material - For black patients, a culturally sensitive educational approach was used: use of minority health educators and written brochures that highlight minority transplant recipients and their living donors and we integrated race-specific data into the discussion (e.g. unique transplant concerns of Blacks, specific LDKT rates) |
| Tolman, A. et al. (1998) | USA | Native Americans | Implementation of a culture-specific intervention for a Native American community | Improve access to psychiatric services for Native Americans | - Needs-assessment with hospital administrator, a Native American social worker, three Native American patients and tribal elders - Installment of a Sweat Lodge on hospital property constructed by officially sanctioned representatives of the Reservation with appropriate materials according to Native traditions - Ceremonies at the Sweat Lodge were led by tribal elders and included Native American patients, Native and non-Native staff from the hospital and interested consumers |
| Trinh, N.T. et al.  (2014) | USA | Latinos | Culturally Focused Psychiatric Consultation (CFP) Intervention for Latino Americans with Depression | Improve depression treatment for Latino patients | - The CFP consultation included two visits: - Initial assessment by a clinician (psychiatrist or psychologist): - After engaging patients, clinicians reviewed with them their diagnoses and treatment recommendations - Follow-up after 2 weeks: visit with their CFP clinician to address their use of and questions about the toolkit. - Clinicians shared recommendations with primary care providers via e-mail. - Consultation provided in English or Spanish - Clinicians used the Engagement Interview Protocol (EIP) which integrates patients’ illness beliefs into psychiatric assessment and evaluation to improve the acceptance of psychiatric treatment among culturally diverse populations - All clinicians were trained specifically in the use of the EIP model - Clinicians develop co-constructed illness narratives with patients and reframe the Western concept of depression into more culturally resonant forms - Materials were also available on audio compact disc (CD) in English and Spanish. |
| Tu, S. P. et al.  (2006) | USA | Chinese Americans | Health promotion for promoting fecal occult blood test (FOBT) screenings for Chinese patients | Improve colorectal cancer screening rates | - Bilingual materials (a video, a motivational pamphlet, an informational pamphlet, and FOBT instructions) - Clinic-based education promoting fecal occult blood testing (FOBT) screening carried out by a trilingual and bicultural health educator |
| Vargas, R.B. et al.  (2008) | USA | Minority women | The original patient navigation programs to reduce disparities in the diagnosis and treatment of breast cancer | Improve diagnosis and treatment of breast cancer for minority women | - Patient navigation’ is an intervention that was designed and implemented to reduce disparities in breast cancer care for poor women in the largely African-American and Latino community of Harlem, New York City, in 1990. |
| Watkins, E. L. et al.  (1990) | USA | Migrant farmworkers | Model program to deliver primary healthcare services to migrant farmworker women and children | Improve healthcare of migrant farmworkers | - The project emphasized coordinated services for migrant farmworker mothers and children - Collection of medical data of migrant farm worker women and children from medical records - Employment of multidisciplinary staff (two nurses, a nutritionist and a social worker all Spanish speaking) - Outreach and early case finding (Home visits to women enrolled in the project and provision of guidelines for identifying pregnant women and referring them to the center) - Coordination between the center’s programs and other health and social service agencies - Availability of immunization supplies, protocols, educational materials, and services of consultants - The social worker provided counseling and facilitated access to community resources - Tracking system to encourage continuity of care :Migrant farmworker women and children were given copies of their health records, together with stamped postcards to notify center staff of their new location when they moved. - Prenatal patients were given a bilingual (Spanish-English) prenatal weightgain - Record and another bilingual record, to provide continuous data on the growth of each child. - Health education program conducted to train migrant farmworker women as lay health advisors |
| Weech-Maldonado, R. et al.  (2016) | USA | Culturally diverse patients | National center for healthcare leadership diversity demonstration project | To improve healthcare for culturally diverse patients | - Battery of preassessments - Diversity coach discussed the preassessment results with the leadership team - Development of organizational and individual level action plans in collaboration with leadership team and CEO - Executive coaching and training - Implementation of diversity leadership - Implementation of strategic human resource management - Promotion of patient cultural competency (provision of interpreter services and translated materials for limited English proficient patients, delivery of care, physical environment, and links to supportive services and providers) - Repetition of quantitative assessment battery to determine pre-post intervention change - Post-project feedback and planning for sustainable change and further improvements |
| Wennerstrom, A. et al.  (2015) | USA | Vietnamese Americans | Patient Resource and Education Program (PREP) to Support Disease Self-Management Among Vietnamese Americans | Improve chronic disease management | - Integration of bilingual community health workers (CHW) in patient-centered medical homes (PCMHs) - Each CHW was paired with a medical office assistant (MOA) who served as the primary PCMH contact - CHWs conducted home visits for each patient which included tailored health coaching - Activities of CHW included building patient self-management capacity through individual and family education, collaborative goal setting, providing links to supportive community resources, basic interpretation, patient and community advocacy and interacting with health care providers. - Adaptation wording of Vietnamese documents that were congruent with the local dialect - To accommodate limited literacy we used handouts with colorful diagrams and simple language and visual aids - CHWs called patients weekly to troubleshoot challenges and offer support |
| Yasui, M. et al.  (2014) | USA | Minority families | Culturally Enhanced Video Feedback Engagement Intervention (CEVE) | Improve client engagement in child and family intervention for child disruptive behaviors disorders | - Shared understanding through cultural framing of the family’s problem in a culturally congruent manner based on shared observations - Cultural Ecogram (CE uses pictorial cues of a range of cultural and ecological influences) and Integrated Video Feedback |
| Ye, J. et al. (2012) | USA | Korean Americans | Telepsychiatry service | Improve access to culturally competent care | - Korean-speaking psychiatrist |
| Yu, J. et al.  (2009) | USA | Asian Americans | Culturally Adaptation of the Healthcare Intervention Services (HIS) Model to deliver substance abuse intervention services to Asian Americans | Improve access to substance abuse treatments for Asian Americans | - interventionists who speak the language the client is most comfortable in - Screening: the Simple Screening Instrument (SSI) for alcohol-and drug abuse was translated into seven major Asian languages: Chinese, Korean, Japanese, Vietnamese, Hindi, Farsi, and Bengali. - Brief intervention: Clients assessed as “at risk” would receive a brief intervention. The interventionist specifically addresses any misunderstandings or barriers to treatment common among Asian Americans. A take home package in their own language. - Full intervention with a referral: Services offer clients with a substance abuse problem the opportunity to change the direction of their lives. - follow-up would provide the opportunity to assess the efficacy of the intervention and, if needed, an opportunity for further intervention. |
