# Supplementary material for: Culturally competent healthcare – A scoping review of strategies implemented in healthcare organizations and a model of culturally competent healthcare provision
Source: PLoS One. 2019 Jul 30;14(7):e0219971. doi: 10.1371/journal.pone.0219971 (PMC6667133; doi:10.1371/journal.pone.0219971)
Supplement: S3 Table — (DOCX) [file pone.0219971.s004.docx]

**S3 Table. Study characteristics and results**

| Authors | Outcome measures | Study type | Study participants | Results |
| --- | --- | --- | --- | --- |
| **Aggarwal, N. et al.**  **(2015)** | - CFI feasibility  - CFI acceptability  - CFI clinical utility | Qualitative study:  Interviews with patients and clinicians | ***Participants (N=46)***  Patients (n=32 patients)   - Non-Hispanic White: 4 - African-American: 5 - Hispanic/Latino: 22   Research clinicians (n=7)   - Non-Hispanic White: 2 - Hispanic/Latino: 3 - Asian: 1   Mixed: 1 | - Patients and clinicians thought that the foremost communication functions of the CFI were to   - Determine and monitor the nature of the problem   - Develop, maintain, and conclude the therapeutic relationship   - Patient education and implementation of treatment plans - We found four main tasks within these functions:   - Enhancing rapport through satisfaction with the interview   - Eliciting data   - Eliciting the patient’s perspective   - Perceiving data at multiple levels   - Communication care   - Recognizing communication barriers - Most communication functions of the CFI fell under clinical utility with only some under acceptability and none under feasability |
| **Alegría, M. et al. (2008)** | - changes in self-reported patient activation - changes in self reported patient empowerment - treatment attendance (defined by the proportion of visits attended divided by the number of visits scheduled)   retention in treatment (defined as 4 or more visits during the 6-month follow-up period) | Controlled before-after study | ***Pretest (N= 231)***  Intervention (n=141)   - White: 5 (3.55%) - Latino: 117 (82.98%) - African American: 10 (7.09%) - other: 9 (6.38%)   Control (n=90)   - White: 18 (20.0%) - Latino: 68 (75.56%) - African American: 1 (1.11%) - other: 3 (3.33%)   ***Posttest (N=221)*** | - No evidence of an effect on self- reported patient empowerment. - Statistically significant effect of the intervention on self-reported patient activation (*p*=.049) - Intervention participants were 29% more likely to attend their scheduled visits than comparison participants (OR=1.29, 95% CI=1.16 –1.43)   Intervention participants were over twice as likely to be retained in treatment (adjusted OR=2.78, 95% CI=1.33–5.79) |
| **Anand, K. J. S. et al. (2015)** | - Mortality rate at discharge | Controlled before-after study | ***Preintervention (N= 3891)***   - African American: 2105 (54. 1%) - White American: 1601 (41.1 %) - Latino: 185 (4.8 %)   ***Postintervention (N= 4179)***   - African American: 2287 (54.7%) - White American: 1718 (41.1%)   Latino: 174 (4.2%) | - Odds of mortality decreased significantly for Latino children (OR, 0.24; 95% CI, 0.06-0.88; *p* =.03) but not for white and African American children (OR, 1.02; 95%CI, 0.73-1.43; *p* =.90) |
| **Armengol, C. G. (1999)** | - Perceived abilities to cope efficaciously - Impact of those perceived abilities on mood and expactations of success | Interrupted-time-series study (before-after + follow up) | ***Participants (N=6)***   - 100% Latinos/Hispanics | - A significant difference in scores between pre- and postintervention assessments was obtained on all measures ( Feelings of hopelessness, self-regulatory difficulty, sense of purpose; *p*<.005) |
| **Aviera, A.**  **(1996)** | - Not specified | Case report study | - Group size ranged from 5 to 10 patients   Immigrants from Mexico and Central America, Cuba and Puerto Rico | - Dichos are usefull for building rapport, decreasing defensiveness, enhancing motivation and participation in therapy, improving self-esteem, focusing attention, facilitating emotional exploration, articulating feelings, developing insight, and exploring cultural values and identity |
| **Barrio, C. et al.**  **(2010)** | ***Family outcomes***   - knowledge of the illness - family burden - family well-being, family warmth - expressed emotion - coping - social support - cultural concepts domains - treatment satisfaction   ***Client outcomes***   - symptom severity - quality of life - family warmth - medication compliance - service use | Mixed Method Design  Concurrent Triangulation   - Quantitative: RCT   Qualitative: Focus groups with patients | ***Dyads (N=59)***   - Intervention (n=26 dyads) - Control condition (n= 33 dyads)   Mexican Americans:  82% of family members born in Mexico  52% of clients born in USA | ***Perceived Change in Knowledge***   - increased illness knowledge - increased cultural knowledge through culturally supportive environment and cultural validation - appreciated psychoeducational material that elucidated a biopsychosocial perspective while validating their Latino family cultural context - renewed cultural pride in their Mexican heritage   ***Perceived Change in Attitude***   - change of attitude from one of stigma attached to the illness to one of acceptance and understanding   ***Perceived Change in Practices and Behaviors***   - a greater sense of empowerment in becoming more assertive about seeking treatment |
| **Bekaert, S. (2000)** | Improvements in care | Good practice case study | Not specified | - Improvements have been made to policies, plans and practices of health and social services in Oxfordshire  - Information folder were not used to capacity  - Data collection was still not carried out regularly because staff felt it would be an imposition  - Opportunity to choose a ethnic meal was not taken up because clients didn't trust the mechanism of provision  - Relatives were still translating for patients  - Poor service response to language needs due to costly systems |
| **Bender, M. et al.**  **(2013)** | - children’s consumption of sugar-sweetened beverages (SSB) - mothers’ pedometer steps   BMI | Interrupted-time-series study (before-after + follow up) | ***Mother-Child Dyads (N=33)***  Mothers: 100% born in Mexico  Children: 100% Hispanic | - By postintervention, SSB consumption declined significantly for soda by 82% (*p*<.0167), and other sugary drinks by 73% (*p*<.0167), whereas decreased consumption for juice by 30% was not significant - Maternal step counts significantly increased for weekdays by 69% and weekend days by 49%   Maternal BMI decreased significantly(*p*<.05) while children’s BMI% remained stable |
| **Beune, E. et al. (2014)** | - *Primary outcome*: between-group difference in the proportion of patients with a SBP reduction of at least 10 mmHg at 6 months - *Secondary outcome*: mean between-group differences in changes in SBP and DBP and adherence to lifestyle and medication recommendations from baseline to 6 months | Controlled cluster-randomized trial | ***Participants (N=149)***  Intervention (n= 71)   - Ghanaian: 20 (28%) - Surinamese: 51 (72%)   Control (n= 68)   - Ghanaian: 26 (38%) - Surinamese: 42 (62%) | - SBP level decreased by at least 10 mmHg among 48% of the patients in the intervention group, compared to 43% of the patients in the control group. After adjustment the estimated between-group difference was not significant (*p*=0.19) - The mean SBP and DBP level in the intervention and control group decreased from baseline to follow-up assessment. After adjustment the estimated between-group difference in improvement was statistically significant for DBP (*p*=0.03) but not for SBP |
| **Carrillo, J. E. et al.**  **(2011)** | - Visit to emergency department - inpatient hospital utilization metrics | Interrupted-time-series study (before-after) | ***Patients (N=5963)*** | - Mean visits per patient to the emergency department decreased 9.2% after implementation of the patient-centered medical home model: 0.59 (SD=1.176) vs. 6-months later: 0.53 (SD=1.083); *p*=0.001 - During the same period, hospitalizations for the cohort dropped 5.8 % decrease: 1503 vs. 6 months later:1416; *p*=0.25 |
| **Chow, W. et al.**  **(2011)** | - Reduction in hospital days - Total admissions - Brief Psychiatric Rating Scale (BPRS) scores - Clients satisfaction | Historically controlled study | ***Mount Sinai Hospital ACT (N=66)***   - Chinese: 30 (46%) - Tamil: 12 (18%) - Vietnamese: 9 (14%) - Caribbean: 7 (10%) - Other: 8 (12%)   ***KUINA Center ACT (N=40)***  Japanese: 40 (100%) | ***Mount Sinai ACT***  - Mean reduction of hospital days: 78% (from 97 to 21 days)  - Reduction of total admission: 57% (from 104 to 45)  - BPRS scores: from 57 (SD=20) to 45 (SD=6), *p*<.001  - Satisfaction ratings in the “satisfied” and “very satisfied” categories: 45 clients (91%) and 14 family (100%)  ***KUINA ACT***  - Mean reduction of hospital days: 72% (SD = ±48%, from 60±115 to 17±60 )  - Reduction of total admission: 57% (from 14 to 8)  - BPRS scores: from 58 (SD=22) to 47 (SD=3), *p*<.005   - - Satisfaction ratings in the “satisfied” and “very satisfied” categories: 40 clients (95%) and 20 family (90%) |
| **Cooper, L. A. et al. (2011)** | - Physician communication behaviors - Patient ratings of physicians’ participatory decision-making (PDM) - Patient involvement in care (PIC) - Reported adherence to medications - Systolic and diastolic blood pressure (BP) and BP control | Randomized controlled trial | ***Clinicians (N=41)***  *Intensive (n=22)*  African American: 5 (23%)  Asian: 6 (27%)  White: 10 (45%)  Hispanic/Latino: 1 (5%)  *Minimal (n=19)*  African American: 7 (37%)  Asian: 4 (21%)  White: 8 (42%)  ***Patients (N=279)***  *Physician + Patient Intensive (n=83)*  African American: 52 (62.6%)  Asian: 2 (2.4%)  White: 29 (34.9%)  *Physician Minimal/ Patient Intensive (n=57)*  African American: 38 (66.7%)  Asian: 1 (1.8%)  White: 18 (31.6%)  *Physician Intensive/ Patient Minimal (n=84)*  African American: 51 (60.7%)  American Indian: 1 (1.2%)  White: 32 (38.1%)  *Physician + Patient Minimal (n=55)*  African American: 32 (58.2%)  American Indian: 1 (1.8%)  White: 22 (40.0%) | - Verbal dominance diminished (improved) significantly in all patient visits relative to the simulation with little difference by the physician intervention group. - Patient report of physicians’ PDM showed significantly greater improvements among patient+ physician intensive group than the patient + physician minimal group - Two aspects of the PIC, physician facilitation and information exchange, showed improvements for intensive relative to minimal groups. - Changes in patient-reported adherence to medications at 12 months did not differ for any of the intervention groups compared to the patient+physician minimal intervention group. - In the overall sample, changes in systolic and diastolic BP at 12 months did not differ for any of the intervention groups when compared to the patient+physician minimal intervention group. However, among patients who had uncontrolled BP at baseline, large reductions in systolic BP were observed among patients in the patient+physician intensive |
| **Cooper, L. A. et al. (2013)** | - Depression symptom reduction - Patient reports of depression treatment - Patient ratings of their clinicians participatory decision-making skills - Patient ratings of the depression case manager and adherence to case management | Cluster randomized controlled study | ***Participants (N=159)***  *Primary care clinicians (n=27)*  Intervention (n=11)   - African American: 5 (45%) - White: 6 (55%)   Control (n=16)   - African American: 3 (19%) - Asian: 6 (38%) - White: 6 (38%) - Other: 1(6%)   *Patients (n=132)*  Intervention (n=67)  Control (n=65)  All African American | - Both groups experienced statistically highly significant reductions in mean depression severity score and in mental health functioning - At 12 months, 33% of the patient-centered group and 42% of the standard group achieved remission from depression; this difference was not statistically significant - Patient self-report of taking any anti-depressant medication remained low throughout the study regardless of intervention assignment. Between-group comparisons from baseline to 12 months were not statistically significant for any type of depression treatment. - At enrollment, shortly after the clinician intervention, 73.4% of patients in the patient-centered versus 62.9% of patients in the standard group rated their clinician as participatory - There were no statistically significant differences in the odds of rating their clinician as participatory among patients in the patient- centered versus standard group from baseline to 12 months and from baseline to 18 months. - At 12 months, patients in the patient-centered group had statistically significantly higher odds of rating their DCM as extremely helpful at identifying concerns and improving adherence to treatment |
| **Coronado, G. D. et al**  **(2011).** | - Our primary outcomes: postintervention differences between intervention and usual care groups in FOBT screening participation. - Secondary outcomes: awareness and intention to receive an FOBT test | Randomized controlled trial | **Patients (N=635)**   - usual care group (n=165) - mailed FOBT only group (n=168) - mailed FOBT and outreach group (n=168)   All Hispanics | - Screening rates were 26% mailed FOBT only and 31% in the mailed FOBT and outreach groups both were statistically larger than the usual care group (*p*<.001) - Awareness of FOBT varied across intervention arm (usual care: 60%; mailed FOBT only: 84 %; mailed FOBT + outreach: 79%; *p*<.01) - Substantial differences were noted in the proportions of respondents who were considering having an FOBT in the next few months (usual care: 35%; mailed FOBT only: 57%; mailed FOBT+outreach: 68%; *p=*.07) - Having heard of a colonoscopy and attitudes about colorectal cancer and screening were similar across intervention arms. |
| **Culica, D. et al. (2008)** | - Health indicators: HbA1c level, blood pressure measurements, height measurements, weight measurements, body mass index (BMI) | Interrupted-time-series study (+ comparison with partial participation group) | ***Participants (N=55)***   - Full participators group (n=36) - Partial participators group (n=19) - African American: 9 (16.36%) - Hispanic: 42 (76.36%)   Other: 4 (7.27%) | - There were significant reductions in mean HbA1c in the full participators group (*p*<.05) - In the partial participators group, the mean HbA1c was not changed significantly from baseline to six months, but it was significantly reduced at 12 months when compared with baseline HbA1c (*p*<.05) - The logistic regression showed the effectiveness of having a community health worker as the sole diabetes educator delivering the CoDE educational program during the study period |
| **Dahhan, N. et al.**  **(2012)** | - Feasability of the MOC (e.g. the number of patients attendance, willingness from patients to participate) - Influences on the health care process (e.g. knowledge of disease, adherence to treatment, Dutch language skills) - Use of hospital facilities (the number of admissions, visits to the POPD and ED | Historically controlled study | ***Patients and their parents (N=189)***   - Moroccan: 100 (53%), - Turkish: 51(27%), - Surinam: 13 (7%)   “other ethnic minority”: 25 (13%) | - Over 95% of the parents were willing to participate and more than 90% of the parents proved to be satisfied with the MOC. - The ability to speak and understand Dutch was sufficient in 58% and health literacy in 88%. - Knowledge of disease was sufficient in 59% and the sense of disease severity in 69% of the parents. - In patients with asthma the number of admissions and visits to the POPD was significantly reduced. Unclear result for patients with diabetes and metabolic disease. |
| **Delphin-Rittmon, M. E. et al.**  **(2016)** | - Individual cc - Organizational cc | Interrupted-time-series study (before-after) | ***Provider assessment (N=34)***   - White: 65% - Black: 29% - Hispanic or Latino: 8% - Asian: 3% - Missing: 3%   ***Organizational cc assessment (N=125)***   - White: 78% - Black: 18% - Hispanic or Latino: 10% - Other: 3% - Asian: 2%   Indian/Native American: 2% | - Significant increases in providers’ multicultural knowledge, awareness, and skills. - Qualitative responses demonstrated the contribution of the experiences of persons in recovery to the training - Significant improvements in the agency’s cultural competence policies (e.g., implementation of strategies to hire and retain a diverse workforce) |
| **Doorenbos, A. et al.**  **(2011)** | - Receipt of individual cancer screening procedures (lung, breast, colorectal and prostate cancer) | Randomized controlled trial | ***Participants (N=5 633)***   - Intervention (n=2695) - Control (n=2668)   All were Native Americans | - The calendar with health messages did not result in increased receipt of any cancer-related outcomes compared to the calendar without health messages |
| **Edwards, G. et al.**  **(2011)** | - Staff knowledge about breastfeeding - Women's knowledge about breastfeeding - Satisfaction with the advice women have received | Mixed Method Design:  Concurrent Triangulation   - Quantitative: Interrupted-time-series study (before-after)   Qualitative: Interviews | ***Quantitative (N=279 staff members)***   - Ethnicity not specified   ***Qualitative (N=8 women who had given birth 24hr before)***   - Indian: 2 - Phillippines: 2   Pakistan, Ukraine, Jordan, Egypt: 1 each | - Mean knowledge score of staff members increased form 76.44 to 83 - Breastfeeding rates on discharge and skin-to-skin contact increased - There were 4 clear themes that emerged from the interviews with the women: Positive education, positive experience, learning something new, incidental unrelated issues - Women were very satisfied with the help and support they had received. But lack of information for women who could not write or read. |
| **Ferdinand, L.A.**  **(2009)** | - Patient participants’ perceived level of cultural sensitivity in healthcare - Systolic and diastolic blood pressure | Controlled before-after study | ***Health care providers (N=32)***   - African American: 9.4% - Asian American/Pacific Islander: 3.1% - European American: 75% Hispanic: 9.4% - “other“: 3.1%   ***Office staff (N=16)***   - African American: 25% - European American: 75%   ***Patients (N=224)***   - African American: 49%   European American: 51% | - The cultural sensitivity ratings of providers’ behaviors and attitudes by African American patients increased in both clinics. At the intervention clinic more than their counterparts at the control clinic at post-intervention but the difference was not significant. - The mean diastolic and systolic blood pressure readings of patient participants did not significantly differ from intervention to control group |
| **Galvin, S. et al. (2008)** | - "Breastfeeding initiation“: women offering any amount of breastmilk to their infants in the hospital | Historically  controlled study | ***Before intervention (N= 197)***   - Cambodian: 12 - Non-Cambodian: 175   ***After intervention (N= 163)***   - Cambodian: 12   Non-Cambodian: 151 | - Prior to the intervention, Cambodian mothers were significantly less likely to initiate breastfeeding than non-Cambodian mothers (16.7% Cambodian vs. 60.6% Non-Cambodian *p*=.003). - Post-intervention, no significant difference between breastfeeding initiation rates among Cambodian women and non-Cambodians (66.7% Cambodian vs.68.9% Non-Cambodian *p*=.874) |
| **Garvin C.C. et al.**  **(2004)** | - Quantitative: diabetes knowledge, social support level, attitudes, self-efficacy, and health status - Qualitative: experiences in REACH activities, and in living with diabetes, following their REACH involvement | Mixed Method Design  Congruent triangulation:   - Quantitative: Interrupted-time-series stay (before-after)   Qualitative: Focus groups | ***Patients (N=396)***  Quantitative data (n=348)  Qualitative data (n=48)  All African American, Latino or Asian | - Significant increase in physical activity from 76% to 84%, in knowledge on how often hemoglobin A1 c should be checked from 54% to 69%, in knowledge on the best way to take care of their feet form 77% to 85%, to be able to care for diabetes including reported ability to keep blood sugar in good control form 48% to 58%, to keep weight under control from 44% to 55% - positive and significant changes in 10 of 12 specific items of dietary behaviors - People felt that they had not been fully informed by their clinicians, and believed that more information would assist them in adapting to their chronic disease - Through the classes and support groups, participants learned how to prepare for their doctor appointments and class activities, conducting intervention groups in their native languages, contributed to their knowledge about diabetes. - People felt that they were better able to treat their disease and more comfortable talking about their diabetes with their families and friends and they felt more confident and in control of their lives - In addition to what they eat, many participants have changed how much they eat. Other changes that focus-group respondents reported concerned physical activity and interacting with doctors. |
| **Gary, T. L. et al.**  **(2009)** | - Primary outcome: emergency department (ER) visits - Secondary outcomes: hospitalizations, hemoglobin A1c levels | Randomized controlled trial | ***Patients (N=488)***  Minimal (n=253)  Intensive NCM/CHW (n=235)  All African American | - At 24 months, the intensive intervention group had fewer ER visits (23%) compared with the minimal intervention group. A similar trend was shown for hospitalizations (9%). - At 36-Months: those who had a higher frequency of CHW visits, but not necessarily NCM visits, were significantly less likely to have ER visits and hospitalizations compared with the minimal intervention group (*p*<.05) - Mean (SD) within-group changes for the intensive group were favorable for HDL-C and diastolic blood pressure (*p*<.05) |
| **Gerrish, K. et al.**  **(2004)** | - Perceived degree of ‘success’ of the program and the basis on which these judgements were made | Qualitative study | ***Oversea nurses (N=17)***  From China, the Philippines, India, and Sub-Saharan Africa | - Enabling the overseas nurses gain professional registration was stressed by all stakeholders. - The success of the program depended on the extent to which overseas nurses fulfilled the expectations of a registered nurse in the area in which they worked. Whereas the nurses were developing new skills, they were also constrained by restrictions on their scope of practice. - The program was resource intensive in terms of the amount of support required to enable the nurses to register. Stakeholders questioned the cost-effectiveness of this approach to tackling recruitment - Most overseas nurses wanted to continue their professional development by undertaking education program relevant to their area of practice. - Senior nurses and many ward managers saw one of the main benefits of overseas nurse recruitment as promoting the ethnic diversity of the nursing workforce. It was anticipated that overseas nurses would contribute through sharing their international experience. |
| **Gil, S. et al.**  **(2016)** | - The level of satisfaction and experiences of parents and health care providers | Mixed Methods Design  Congruent Triangulation:  Quantitative: cross-sectional study  Qualitative: open-ended question | ***Participants (N=27)***  Parents of different patients (n=12)  health care staff (n=15) | - Six themes were identified from the qualitative data:   - Continuity of Care: health staff recognized that the LEP patients and families were at risk for fragmented care, and the presence and actions of the LEP Patient Family Advocate helped address that risk.   - Decreasing Stress and Burden: Continuity in care improved communication and also decreased parents’ stress.   - Patient Safety: Both staff and parents felt that the LEP Patient Family Advocate role enhanced the safety of complex chemotherapy treatment and created safer systems for families caring for their child at home   - Trust and Connectedness: Parents expressed trust in the LEP Patient Advocate’s competence as an interpreter, which helped them feel understood. Staff identified the benefits of a consistent, specialized interpreter. Staff also recognized that the social conversations that help develop trust are easily lost when there is a language barrier, but the LEP Patient Advocate helped create that level of interaction.   - Communication: Parents recognized that the LEP Patient Advocate helped them be understood and enhanced the ability to communicate about their child.   - Respect. Parents and staff recognized that the LEP Patient advocate role facilitated effective communication, which also demonstrated respect for patients and families. - High levels of satisfaction were reported (between 4.2 and 5 of 5) |
| **Gilmer, T. P. et al.**  **(2005)** | Clinical outcomes (hemoglobin HbA1c, blood pressure, cholesterol level)   - costs | Controlled before-after study | ***Participants (N=348)***  Intervention (n=188)   - Latino: 70 (37%) - non-Latino white: 51 (27%) - Asian: 36 (19%) - African American: 7 (4%) - other/unknown: 24 (13%)   Control (n=160)   - Latino: 48 (30%) - non-Latino white: 35 (22%) - Asian: 27 (17%) - African American: 11 (7%)   other/unknown: 39 (24%) | - Participation in Dulce was related to significant improvements in HbA1c (*p*=0.001), both systolic and diastolic blood pressure (*p*<0.001), total cholesterol, and LDL-C (*p*<0.05). - Total costs were higher for Dulce participants during the first year of disease management. Total costs, however, mask an interesting offset. While expenditures for pharmacy/supplies and disease management increased under Dulce, expenditures on hospital and emergency department care declined, although the change was not statistically significant. |
| **Goncalves, M. et al. (2013)** | - Receiving adequate treatment - (ER) use of patients with a psychiatric diagnosis   Receipt of any inpatient care for a psychiatric diagnosis | Cross-sectional study with comparative group | ***PMHP (N=828)***  ***Non-PMHP (N=500)***  ***Total (N=1328)***   - White: 55.4% - Black: 2.1% - Hispanic: 2.4%   Other: 40.0% | - PMHP patients were more likely to receive adequate care ( ≥8 psychotherapy visits: PMHP: 58.5%vs. non-PMHP: 30.4 % – Difference: 28.1%; ≥4 visits with Pharmacotherapy: PMHP: 67.5% vs. non-PMHP: 38.8% – Difference: 28.7%). - No significant differences identified for ER use and inpatient care. |
| **Halcon, L. L. et al.**  **(2010)** | - Feasibility - Accessibility - Acceptability   of the HR intervention | Mixed Method Design  Concurrent Triangulation   - Quantitative: non-controlled before-and-after study - Qualitative: analysis of session evaluation transcripts | ***Participants (N=19)***  Somali. 9  Oromo: 10 | - 100% of participants were retained and 93% maintained attendance over the eight sessions - Participants rated transportation and child-care support as essential, and food as enjoyable but nonessential. - Ratings on cultural relevance and translation were positive - Statistically significant difference in the groups’ mean posttest scores |
| **Hamilton, L. J. et al.**  **(2013)** | - Family experiences of health care | Cross-sectional with comparative group | - Children (patients): 41 - Parents: 22   Primary English speakers: 14 Primary Spanish speakers: 27 | - The mean score of positive parental perceptions of health care of Spanish speaking families was 8.5 points higher then of English speaking families (*p*=.003) - No statistically significant differences were noted in individual questions between Spanish and English speakers |
| **Hatcher, S. et al.**  **(2016)** | - Primary outcome: the self-rated change in scores on the Beck Hopelessness Scale - Secondary outcomes:   - measures of anxiety, depression, suicidiality and quality of life   - sense of belonging   - cultural impact profile of Maori cultural knowledge   - repeated self-harm - health service use | Zelen randomised controlled trial | ***Patients (N=167)***  Intervention group (n=95)  Control group (n = 72)  All were Maori | - Intervention group: greater change in hopelessness scores at 3 (-4.5 vs. -1.3) and 12 months (-6.6 vs.-2.8) - The proportion of people re-presenting to emergency departments for reasons other than self-harm after 12 months was significantly lower in those who consented to the intervention (44.2 vs. 61.1 %; *p*=0.03) - No significant differences between the groups on any of the continuous outcome measures at 3 or 12 month follow up |
| **Hudelson, P. et al. (2014)** | - Staff knowledge - Attitudes and reported practices regarding the care of migrant patients | Interrupted-time-series study (before-after) | ***Before intervention (N=1336)***  Swiss: 676 (51.1%)  Other: 646 (48.9%)  ***After Intervention (N=745)***  Swiss: 431 (58%)  Other: 312 (42%) | - Respondents significantly more likely to have received training on working with an interpreter - Respondents were significantly more likely to have had contact with several Migrant Friendly structures at the HU - Respondents were significantly more likely to be encouraged by their supervisors to use professional interpreters and to work effectively with an interpreter - Respondents were significantly less likely to rate as source of difficulty: lack of experience with migrant patients, patient’s lack of French, lack of access to professional interpreters, lack of translated patient materials, patient’s lack of knowledge of how hospital functions |
| **Ivey, S.L. et al.**  **(2012)** | - HbA1c value | Controlled before-after study | ***Patients (N=92)***  Primary language spoken at home:  Intervention group (n=46)  Cantonese: 30 (65.2%)  Mandarin: 9 (19.6%)  English: 1 (2.2%)  Other: 4 (8.7%)  more than one language: 2 (4.4%)  Control group (n=46)  Cantonese: 37 (80.4%)  Mandarin: 3 (6.5%)  Other: 5 (10.9%)  more than one language: 1 (2.2%) | - No statically significant differences in the mean change in HbA1c level. - The mean baseline HbA1c was similar among participants in the intervention group and in the control group (intervention: 7.60 vs. 7.62) - At follow-up, between 5 and 8.5 months after the initial physician visit, mean HbA1c had decreased among intervention participants, while mean HbA1c among control participants increased slightly (intervention: 7.24 vs. 7.63) |
| **Kalister, H. et al.**  **(1999)** | - Safety and patient satisfaction of pharmacist service - The appropriateness of the pharmacy treatment | Cross-sectional study | ***Participants (N=232)***  Patients (n=191)   - Somali: 46 (24.0%) - Vietnamese: 30 (15.7%) - White: 29 (15.2%) - African American: 27 (14.1%)   Parents (n=41) | - 22 children returned to the clinic for an unscheduled acute care visit within 1 week of a pharmacy evaluation (14 for reasons unrelated to the initial pharmacist encounter, 3 because the condition for which they had been seen had not improved, 4 patients returned with usual or expected complications of the initial illness as diagnosed at the pharmacy visit) - Patient's satisfaction: Parents were similarly confident in the care that their children received from both pharmacists and physicians. Pharmacist and physician interactions differed most in the wait time and consistency of supplying written information - Detailed retrospective review of 48 medical records revealed minor documentation errors in 12 (25%), major documentation errors in 2 (4%), and failure to refer to a physician when stipulated by protocol in 7 (15%) |
| **Kanter, J. et al. (2010)** | - Treatment adherence - Treatment drop-out and completion - Initial estimates of effect sizes and clinical significance for the Beck Depression Inventory-II and the Hamilton Rating Scale for Depression | Interrupted-time-series study (before and after) | ***Participants (N=10)***  Latinas from:   - Mexico: 60% - Puerto Rico: 30% - United States:10% | - Therapists reported engaging in a mean of 3.24 BA techniques per session (of 4 possible techniques). - Specific activation assignments were reported being scheduled in 93% of sessions - Most homework was partially completed in 84% of sessions - Mean completed sessions 7.7 (Md = 10) over mean weeks 12.4 (Md = 10) - There was a significant decrease in depression severity on the BDI-II (*p*=.002, d=1.67 ) the HRSD (*p*<.001, d=1.57) |
| **Karmali, K. et al.**  **(2011)** | - Effectiveness of the cultural competence initiative - Discipline- and department- specific participation in the workshops - Practice change (Change in interpreter service use) - Patient satisfaction (survey) | Mixed Method Design:  Concurrent Triangulation   - Quantitative: Interrupted-time-series study (before-after)   Qualitative: Interviews, commitment documents | - About 800 workshop participants completed the commitment-to-change activity - 2 542 commitments were documented   54 participants were followed up and interviewed | - Over 2,100 hospital staff attended the workshops - 78% of commitments documented had been achieved and participants indicated intent to act on another 16% of commitments. - Results show a significant increase in the use of face-to-face interpretation and a doubling of the number of minutes of telephone interpretation use - Results indicate a more than 5% increase in satisfaction |
| **Kim, J. et al.**  **(2015)** | - Utilization measurement - System and use measurement - Community health provider feedback - Operational cost measurement | Mixed Methods Design  Congruent triangulation:  Quantitative: cross-sectional study  Qualitative: open-ended survey | ***Screening (N=524)***  ***Information quality ratings (N=7) Community health provider feedback (N=11)***  All First Nations (aboriginal Canadians) | - Teleopthalmology clinics were held in 43 of 51 communities - 26.7% of all clients screened were referred for additional treatment - 5 of 7 the respondents were highly satisfied with the system. - 4 of 7 respondents indicated that they used the system constantly throughout the day. - Most providers were very satisfied or satisfied with all aspects - The direct per-client cost of teleopthalmology was less than the direct per-client cost of traditional ophthalmology screening |
| **Kline, K. N. et al. (2016)** | - viewer involvement, - perceived self-efficacy | Randomized controlled trial | ***Participants (N=248)***   - Intervention (n=123) - Control (n=125)   100% Hispanic | - There was a mix of low and high levels of viewer identification among participant, in total 50% demonstrated similarity identification - More than a quarter of participants reported feelings of self-efficacy related to viewing the telenovela: 17% indicated general optimism or motivation for engaging in diabetes self-care, and 10.5% indicated a specific plan for behavior change. |
| **Kurth , A. E. et al.**  **(2016)** | - Intervention effectiveness: HIV-1 viral load , adherence to medications and sexual transmission risk behaviors - Acceptability of the tool among participants, - Acceptability and system efficiency issues among clinic providers | Mixed Method Design  Concurrent Triangulation   - Quantitative: RCT   Qualitative: Focus groups with providers and exit interviews with particiapants | ***Participants (N=433)***   - Intervention (n=226) - Control (n=207)   Latino:   - Intervention: 218 (96.9%)   Control:191 (92.7%) | - Although intervention participants had reduced viral loads (*f*2=0.0003), increased ART adherence (*f*2=0.002), and decreased sexual transmission risk behaviors over time, patterns of change in the intervention group were not more favorable than in the control group. - The majority of participants were comfortable discussing medications and sex with their providers. Some participants did not because they felt they had other resources for information. - The majority of participants reported a positive experience with using the tablet computer. - Providers agreed that multiple approaches in HIV treatment are important. However, they felt that there are no substitutes for the provider-patient relationship, personalism in Latino culture, and loyalty to a provider. |
| **La Roche, M. J. et al (2011).** | - Cognitive and physiological symptoms of anxiety - Depression and distress levels - Self-orientation - Number of times participants practiced the relaxation tape during the week (Weekly log) | Interrupted-time-series study (3 measures) | ***Participants (N=44)***  All Latinos/as:   - Puerto Rico: 20 (45.5%) were of - Dominican Republic: 19 (43.2%) - other Latin American countries: 5 (11.4%) | - Level of anxiety decreased from the baseline assessment to the post-CCRI assessments (*p*<.01) - Significant decrease of depressive symptoms, however, these decreases were not clinically significant (*p*=.02) - Treatment adherence on the CCRI was correlated with reductions in anxiety (pr=-.34, *p*<.05), but not with depressive symptoms (pr=-.06; *p*>.05) - Participants practiced AIEs p<.01; M=3.1, SD=1.8) significantly more frequently than IIEs (M=2.1, SD=2.2) |
| **Levin-Zamir, D. et al.**  **(2011)** | - satisfaction and access to care - capacity building among staff - short-term and long-term cost of intervention - health quality indicators - evidence of improved health status | Mixed Method Design:   - Qualitative:   In-depths interviews  Open-ended questionnaires   - Quantitative   Cross-sectional study | ***Ethiopian Immigrants (N=666)*** to assess satisfaction and access to care  Others not specified | - Satisfaction and access to care: Findings from both perspectives showed that the liaisons were integrated into the clinic team work, assisted in in-depth communication to meet the individual’s needs, monitoring medication taking, maintaining contact with the families and performing home visits. The liaisons greatly contributed to building trust and confidence in the efficacy of treatment and in applying health recommendations. - Capacity building among staff: Group discussions with the staff elicited the information needed to understand the contribution of the capacity building component, and what was still lacking in order to support their work with the immigrants on a daily basis. - Resulting use of evidence : The evidence showed that a large number of unnecessary medical tests were performed in clinics where the programme was not implemented. However, in spite of the evidence, many decision-makers still place the burden on immigrants to adapt themselves, after so many years living in the country, preventing the programme to be expanded to all clinics serving the Ethiopian community. |
| **Mauldon, M. et al. (2006)** | - Physiologic (HbA1c, body mass index, lipids) - Psychosocial (diabetes-related distress and health beliefs) - diabetes knowledge and language-based acculturation variables | Interrupted-time-series study (3 measures) | ***Participants (N=16)***  100% Hispanic   - Puerto Rico: 11 (68%) - Caribbean: 2 (13%) - Central/South America: 3 (19%) | - There was a statistically significant reduction in mean HbA1c (2.08% mean reduction in HbA1c, *p*=.001) - Improvement and then slight relapse in total cholesterol (TC) and low-density lipoprotein cholesterol (LDL-C) levels (not statistically significant) - There was a statistically significant increase in average high-density lipoprotein cholesterol (HDL-C) (42 to 45 mg/dL [1.08 to 1.16 mmol/L], *p*<.001) - There was improvement that was statistically significant in knowledge (*p*=.003) - Overall, there was not a statistically significant difference in scores of psychological distress |
| **McMurray, J. et al.**  **(2014)** | - Wait times for physician and specialist care - Access to allied health services and physician specialists - Familiarity with the healthcare system - Perceived family well-being | Interrupted-time-series study (before-after) | ***Government assisted refugees (GAR)***  ***Before intervention (N=466)***  ***After intervention (N=406)***   - 38 different countries of birth   40 countries listed as the country of last residence | - GARs were no more likely to see any healthcare provider after the clinic opened (OR=.99; *p*=0.151) - The likelihood of an individual requiring a physician specialist went down 45 % as a result of seeing an refugee health clinic physician (OR=.55; *p*=.004) - Refugees’ wait time to see a healthcare provider decreased from 30 to 21 days (Ratio of mean=.70; *p*<.001) - Reported improvements in ability to understand health care system’’ was significant at all stages (*p*<.01) - The overall model predicting that GARs ‘‘reported problems accessing healthcare’’ was significant (*p*<.01) - The overall model predicting ‘‘reported improvements in family health’’ was not significant |
| **Mehler, P. S. et al.**  **(2004)** | - Glycemic, lipid, blood pressure control | Retrospective cohort study | ***Russian patients (N=55)*** | - There was a significant improvement in the levels of glycemic, lipid, and blood pressure - Hemoglobin A1c levels: from 8.4% to 8.0% (*p*<.007) - LDL cholesterol: 20% from 126 mg/ dL to 102 mg/dL (*p*<.0002) - Diastolic blood pressure: 82.7 mm Hg to 76.3 mm Hg (*p*<.0002) |
| **Melkus, G. D. et al.**  **(2004)** | - Glycemic control - Weight - Body mass index - Diabetes-related emotional distress | Interrupted-time-series study (before and after) | ***Participants (N=25)***  100% Black women | - The mean fasting blood glucose significantly improved (from 184 (SD=17) to 161 (SD=13.5); *p*<.05) - significant improvement in glycosylated hemoglobin (from 8.0% to 6.9% *p<*.002) - There was a significant decrease in mean BMI from the baseline value (32.1 kg/m2 (*SD =* 7.5) to 31.7 kg/m (*SD=*7.8) *p<*.005). - Mean weight was also significantly decreased (from 193.2 lbs (*SD=*10.3) to 191.0 lbs (*SD=*10.5); *p<*.03) - Significant decrease in level of diabetes-related emotional distress (PAID) was observed (from 49.3 (SD=26) to 40.3 (SD=22); *p*<.01) - DM-knowledge emotional stress increased by one point (not statistically significant) - Self-efficacy did not change significantly |
| **Menon, U. et al. (2008)** | - Feasibility - Perceived risk - Self-efficacy for FOBT - Endoscopy - Knowledge | Mixed Method Design  Sequential Transformative:   - Qualitativ: Focus groups   Quantitative: RCT | ***Participants (N=206)***   - Quantitative (n=7):   2 focus groups   - Quantitativ (n=199)   Intervention (n=101)   - African American: 78 (52.3%) - Others: 23 (46%)   Control (n=98)   - African American: 71 (47.7%)   Others: 27 (54.0%) | - After focus groups changes were made to the program leading to a visually appealing, easy to understand and navigate, self-paced program. - 80% said the education helped them decide to get screening - 49% agreed it helped them overcome barriers - 91% agreed it was useful - 68% thought it raised new concerns about cancer, but only 30% said it made them worry about CRC - 95% agreed their doctor's office should continue giving such education - 99% said they would inform family about the program |
| **Moreno, F.A. et al (2012).** | - depression symptoms - quality of life - functional ability measures | Randomized controlled trial | ***Participants (N=167)***  Intervention (n=80)  Control (n=87)  All Hispanics | - For patients in both treatment conditions, significant reductions in symptom severity were seen over time (all measures changed significantly) - 65 patients (41%) achieved remission in depression but the differences between groups were not significant. - Significant increases in quality of life over time for both treatment groups. - Disability ratings decreased significantly over time for both treatment groups. - A significant main effect of time for disability ratings/depression symptoms/quality of life and a significant interaction of time by intervention favoring the Webcam intervention was observed. |
| **Munoz, R. F. et al.**  **(2007)** | - Postpartum depressive symptoms - Postpartum MDE (major depression episode) incidence | Randomized controlled trial | ***Participants (N=41)***  Intervention (n=21)   - Mexico: 15 (71.4%) - U.S.A.: 4 (19%) - Latin America: 2 (9.6%)   Control (n=20)   - Mexico: 8 (40%) - U.S.A.: 4 (20%) - Other - Latin America: 6 (30%) - Other: 2 (10%) | - No significant group changes in postpartum depressive symptoms. - MDE incidence rates of 14% for the intervention condition versus 25% for the comparison condition represent a small effect size (h = 0.28) |
| **Nowalk, M. P. et al. (2008)** | - Annual influenza vaccination status   - - Cumulative pneumococcal polysaccharide vaccine (PPV) status | Controlled  before-and-after  study | ***Patients (N=568)***   - Health Center A n=151 - Health Center B n=104 - Health Center C n=146 - Health Center D n=106 - Health Center E n=61   Non-white: 349 (61.4%) | - Significant differences between intervention and nonintervention sites overall in Year 1 (from n=13 (21.3%) to n=226 (44.6%), *p*<.001) Year 3 (from n=89 (%28.4) to n=126 (49.4%), *p*=.001) , and Year 4 (from n=12 (19.7%) to n=248 (48.9%) *p*<.001), with no significant difference between intervention and nonintervention sites in Year 2 - The same pattern of change was true for non-whites; although for whites, a significant difference between intervention and nonintervention sites occurred only in Year 1 - Rates of PPV vaccination did not increase significantly. |
| **O'Shaughnessy, R. et al.**  **(2012)** | - Impact of the intervention on the quality of the mother-infant relationship | Mixed Methode Design  Congruent Triangulation:   - Qualitativ:   Focus groups   - Quantitativ:   CARE Index video microanalysis and session-by-session evaluation | - Group sessions ranged between 4 to 12 mothers and babies - Session-by-session questionnaire: n=57 - West Africa (mothers): Gambia, Sierra Leone, Ivory Coast and Nigeria | - 100% felt the group was helpful at every sessions - About 76 % of women reported feeling better after the group - Positive changes in the quality of mother-child relationships were observed - Themes emerging from reflective group conversations: Being together and talking together versus being alone, the experience of feeling safe, learning about motherhood and parenting, mother and baby relationship |
| **Ohr S.O. et al.**  **(2016)** | - usefuless of the support strategies - effectiveness of the support strategies | cross‐sectional study | ***Oversea nurses and midwives (N=65)***   - the countries where their first nursing qualifications were achieved were India (62%), UK (8%), and 10 other countries | - More than 90% of the respondents found the support strategies useful for their transition. The most useful strategies were personal support and a welcoming atmosphere on arrival, and an orientation specifically designed for OQNMs to acclimatize into the Australian culture and nursing workforce. - Other respondents expressed the need to be informed of all available support strategies and a need to have further support. In fact, 66% of them were aware of the Overseas Staff Support Program - All of OQNMs who were supported by the program remain employed in the District, and there have been a decreased number of referrals to the OSSP program manager to address issues of concern once work had commenced - 92% of respondents stated they would recommend, or have recommended the District as a potential employer. |
| **Oppong, B. et al.**  **(2016)** | - Intervals (in days) between the screening and diagnostic visits | Retrospective cohort study | ***Patients (N=4605)***  52% Black  41% Hispanic  4% White  2% Asian   - 1% other | Follow-up after screening mammography   - 451 (9.8%) women required additional workup and were assigned at Breast Imaging-Reporting and Data System (BI-RADS). - The median interval between screening and diagnostic imaging was 39 (range 6-400 days). 29% followed up in ≤ 30 days and 64% returned in ≤ 60 days. - Time to follow-up (in days) after screening mammography was significantly different for „race“ (Black: 37 (6-276); Hispanic: 42 (11-400 ); White: 22 (9-94); Asian: 30 (22-89); *p*=0.03 - No differences in the receipt of additional imaging based on race   Follow-up for biopsy   - 162 out of 451 (35.9%) were recommended to have biopsy. - 132 (81.5%) underwent biopsy within a median of 21 days (interquartile range: 0-221 days). - Time to biopsy (in days) was significantly different for „race“ (Black: 18 (3-116); Hispanic: 34 (1-221); White: 18 (8-88); Asian: 50 (10-60); *p*<.01) |
| **Ortega, A. N. et al.**  **(2002)** | - General psychiatric problems - Depression - Symptoms of psychosis - Alcohol problems - Drug problems - Overall quality of life - Social support | Controlled  before-and-after  study | ***Clients (N= 5795)***   - White: 2333 (42%) - Hispanic: 242 (4%) | - Hispanics had more serious problems than whites on several measures of psychiatric and substance abuse domains - Hispanics showed less improvement than whites on several measures of psychiatric status and service use - Significant association with ethnic matching: when treated by a Hispanic clinician, Hispanic clients showed less improvement in symptoms of psychosis |
| **Poureslami, I. et al.**  **(2016)** | - Participants' ability to use inhalers - Understanding of physicians' instruction on asthma therapy | Randomized controlled trial | ***Participant (N=85)***  Group 1 (n=22)   - Chinese: 12 - Punjabi: 10   Group 2 (n=21)   - Chinese: 11 - Punjabi: 10   Group 3 (n=20)   - Chinese: 9 - Punjabi: 11   Control (n=22)   - Chinese: 10 - Punjabi: 12 | - Among all participants there was a statistically significant difference in mean score of correct use of inhaler over time (*p*<.001) - Only in group 1 the correct use of inhalers improved significantly over time (51%; *p*<.001) - Chinese subjects showed significantly greater improvement compared with Punjabi subjects (*p*<.001) - Understanding Physician's instruction on medication improved significantly in all participants in the 3 groups over time *p*<.008 - Group 1 showed the greatest improvement compared with the 3 other groups (*p*< .039) |
| **Reavy, K. et al (2012).** | - lived experiences - efficiency - effectiveness of the role of health advisor - patient healthcare outcomes | Mixed Method Design:  Concurrent Triangulation   - Quantitative: Retrospective cohort study   Qualitative: observations, focus groups, interview | ***Health advisors (N=14)***  ***Unduplicated prenatal and pediatric patients (N= 227)*** | - decrease of 'no-show' rate, or missed clinic appointments: from 25% to 2.5% - The main themes emerging from the focus group:   *Communicatio*n   - Health advisor role was important for cultural safety as evidenced regarding a stronger refugee voice - The caring and friendship extended to the refugees by the health advisors is the basis for bridging the cultures and establishing trust in the healthcare system - Challenge for the health advisor is that the role is not given the same respect and power as that of CMI   *Navigating the system*   - **T**ransportation to and from the clinic is the one of the first areas that a health advisor assists the refugee - Lack of experience and understanding in navigating the Western healthcare system included using the elevator to access specialty care on the second floor or fears that their blood was being drained when prenatal blood tests were drawn   *Community*   - Clinic creates a community of maternal and pediatric patients within the context of receiving health care and education. - Pregnant women who initially would not make eye contact with anyone, within 2 weeks have been observed to be smiling and chatting with the other patients. |
| **Redwood, D.G.**  **(2016)** | - Program formation/evolution - Outreach responses - Successful components and strengths of the program - Program barriers and challenges | Qualitative study: Interviews | ***Key informants (N=8)***  All Alaska Native and non-Native stakeholders | Key Themes of the evaluation:   - Program formation: Incremental approach to program development - Program evaluation: Additional funding used to hire dedicated outreach staff (patient navigators); improved outreach tracking system and patient materials; improved endoscopic access for patients - Outreach responses: Increased awareness of CRC and the need for screening among population served; fear of finding cancer; wanting to prevent cancer by getting screened; needing help navigating the system to get screened, familial support for screening. - Relatives’ screening rates increased from about 25 relatives screened per year in the early 2000s to 90 screened in 2008. - Strengths: Geographically and ethnically defined patient population; screening costs covered by Alaska Tribal Health System; support by hospital leadership (Chief of Surgery); only program collecting this information and using it for outreach among Alaska Native people, dedicated staff time, Alaska Native patient navigators who receive intensive training in motivational interviewing and patient outreach techniques - Barriers and challenges: Culturally heterogeneous population from across state; still patients due for screening; need for more education on benefits of screening (patients) and screening referral guidelines (providers); reliance on grant funding for program operations; need for improved data sharing and reporting in Alaska Tribal Health System |
| **Riggs, E. et al.**  **(2017)** | - Women’s experiences of the program | Case study | ***Participants (N=19)***  All Karen women from Burma | Experiences with the program:  - Women reported feeling prepared, confident, and reassured, with the greatest benefits coming from shared learning and storytelling with peers, and developing trusting relationships with a team of professionals, all supported by communication in their language.  - Participants reported that care providers were welcoming, valued them, listened, and responded to their needs. The program offered a safe place where women had a sense of belonging and could connect with others and talk in their own language about shared experiences.  Challenges in the hospital:   - Communication and privacy: The only negative aspect of women’s care was related to their experiences in the hospital at the time of childbirth. This was mostly the experience for women booked to another hospital where the caseload midwife did not work. Women mentioned lack of privacy in hospitals as a significant issue of dissatisfaction. |
| **Rodrigue, J R. et al.**  **(2008)** | - Primary outcome: proportion of enrolled patients in both groups with living donor inquiries, at least 1 potential living donor evaluated, and LDKTs 1 year after study participation. - Secondary outcomes: number of individuals who participated in the education sessions and patient’s LDKT knowledge, willingness to talk to others about possible living donor donation, and concerns about pursuing LDKT | Randomized controlled trial | ***Patients (N=132)***  Clinic-based (n= 69)  White: 40  Black: 29  Clinic based+Home based(n= 63)  White: 32  Black: 31 | - Patients assigned to CB+HB group were more likely to had living donor inquiries (OR:1.7; CI=1.2-3), a living donor evaluated (OR:2.7; CI=1.4-5.4) and LDKT (OR: 3.0; CI=1.5-5.9) - White patients were more likely to have had living donor inquiries (OR: 2.4; CI=1.2-4.7), a living donor evaluated (OR: 1.9; CI= 1.1-3.5) and LDKT (OR: 2.0; CI= 1.3-4) - Both black and white patients assigned to the CB+HB group were more likely to have someone evaluated as potential living donor and LDKT than their counterparts in the CB group. - Patients in the CB+HB group showed more improvement in knowledge (CB+HB: +2.8 vs. CB: +1.5), willingness to talk (CB+HB: +2.4 vs. CB: +0.2) and a greater alleviation of concerns (CB+HB: -2.5 vs. CB: -0.1) |
| **Tolman, A. et al.**  **(1998)** | - Utilization rates/ admissions to hospital | Incidence study | Not specified  Caucasian, Black, Hispanic, Asian, Native American | - Sustained and upward shift in the percentage of admissions of Native Americans since the Sweat Lodge and other interactive efforts began - Mean Percentage of Total Admissions before intervention: Caucasian: 86.73; Black: 2.01; Asian: 0.74; Hispanic: 5.76; Native American: 4.77 - Mean Percentage of Total Admissions after intervention: - Caucasian: 84.93 ; Black: 1.63; Asian: 1.16; Hispanic: 4.78; Native American: 7.50 |
| **Trinh, N.T. et al.**  **(2014)** | - treatment satisfaction - acceptability | Mixed Method Design  Congruent Triangulation:  Quantitative: cross-sectional study  Qualitative: In-depht Interviews | **Participants (N=90)**   - Participants for satisfaction scale (n=63) - Participants for in-depth interviews (n=27)   All Latinos | - Acceptability   - the response to the program was very positive   - the vast majority reported that their expectations of the program were met   - The majority of participants reported that they did not discuss the consultation with their PCPs.   - Most believed it was important that their providers speak their language. Some reported that although interpreters are available, waiting for interpreters or having interpreters involved in sensitive medical and mental health conversations were significant challenges - Treatment satisfaction:   - 85 % of participants responded positively to all questions of the satisfaction scale   - 96 % of respondents liked the treatment they received in the program   - Cultural emphasis of clinicians, 98 % agreed that the clinician was sensitive to their culture or background, and 85 % agreed that the clinician understood their culture or background - End-results from the program: 85 % reported that being in the program made them feel less sad, 85 % agreed that the program helped them understand what depression was or how it can be treated, 96 % were satisfied with the results they received, and 98 % would recommend this program to a friend or family member |
| **Tu, S. P. et al.**  **(2006)** | - FOBT screening rates | Randomized controlled trial | ***Participants (N=210)***  Intervention (n= 105)  Control (n=105)  100% Chinese | - Among the intervention patients, 69.5% received FOBT screening, compared with 27.6% of control patients (effect size: 42 % points) - The adjusted odds of FOBT slightly increased greater in the intervention arm compared with the control arm 5.98 (95% CI = 3.29, 10.85) |
| **Vargas, R.B. et al.**  **(2008)** | - Not specified | Qualitative multistakeholder case study:  in-depth, semistructured interviews with patient navigators and their colleagues to describe the original patient navigation programs | Not specified | - Patient navigation operated within a distinct system of care, bound by a specific clinical course, typically starting at the point of a suspicious finding for breast cancer to completing the diagnostic workup, then, if necessary, completion of staging to completion of therapy - We identified 3 core processes in patient navigation systems:   - providing mediators and removing barriers to get patients through the steps of care   - documenting patient barriers and flow   - using the feedback on barriers for implementing system level change - Currently, patient navigation is not a recognized reimbursed service Thus, the programs that we visited use an ad hoc combination of grant funding, overhead funds, and oncology practice reimbursement revenues to support patient navigation |
| **Watkins, E. L. et al.**  **(1990)** | - Children’s birth weight - Children’s immunization - Proportion of women breastfeeding - Utilzation - Impact | Incidence study | ***Pregnant farm worker women (N=359)***   - White: 12 % - Black: 25% - Hispanic: 58 % - Haitian: 5%   ***Children ages birth to 5 years (N=560)***   - White: 10 % - Black: 21% - Hispanic :66 %   Haitian: 2% | - Decrease of children with low birth weight from 13 in to 6 in 2 years - Increase of children with complete immunization from 41% to 63% to 62% - Increase of children receiving a developmental screen from 34 % to 84% to 77% - Increase in prenatal visits from mean of 7.4 to a mean of 9.7 - Increase in proportion of women breastfeeding their newborns from 31% to 52%. - Significant decrease in visits to the center among infants and 1-year-old children: mean number from 4.5 visits 3.1 visits - The population-based approach increased the case finding and follow up activities of the center |
| **Weech-Maldonado, R. et al.**  **(2016)** | - Diversity leadership - Strategic human resource management - Patient cultural competency - Individual level competencies - Diversity climate - Workforce diversity | Controlled  before-and-after  study | ***Staff members (N=287)***   - Nonclinical staff: 6.7% - Clinical support staff or licensed clinicians: 12.9% - Nursing: 64.3% - Medicine: 1.6% - Administration: 14.5% - White: 67.7% - Black: 25.1% - Hispanic: 1.8%   Other: 5.4% | - The intervention hospitals outperformed their respective control hospitals within each health system for change in diversity leadership, strategic human resource management, diversity climate, and all three individual level competencies: diversity attitudes, implicit bias, racial/ethnic identity - Results were mixed for patient cultural competency and workforce diversity |
| **Wennerstrom, A. et al.**  **(2015)** | - Patient satisfaction - Care Team and Project Team Process Evaluation of PREP | Mixed Method Design  Congruent Triangulation:   - Quantitative: Cross-sectional study   Qualitative study: structured team meetings and patient satisfaction surveys | ***Patients (N=31)***  Patients with hypertension (n=17)  patients with diabetes (n=14)  All Vietnamese | - Qualitative themes: the program influenced dietary modifications, home self-monitoring of blood pressure and blood glucose, and medication adherence. Some participants noted that PREP prompted behavior changes among family members who participated in home visits. - 90% of respondents agreed that PREP met expectations and taught them better chronic disease management and that CHW in-person meetings and phone calls were helpful. - CHW–patient Interactions: Positive feedback on cultural appropriateness of patient education but challenges in structuring communications around nutrition, physical activity, and medications, discussing mental health because of associated cultural stigma of depression and feeling unprepared to answer specific questions - CHW care team integration: CHWs were successful in educating about PCMH services, specialty care and health care facilities. - Challenges consisted in establishing routine case conferences: coordinating part-time CHWs and Work flow inefficiency related to processing referrals to CHWs working at an external agency and duplication of MOA and CHW efforts to document care. - Clinical staff did not understand the full range of roles that CHW can fill |
| **Yasui, M. et al.**  **(2014)** | - Session Attendance - Pre-Post Parent Self-Agency Therapeutic Alliance - Cultural Competence | Randomized controlled trial | ***Study families (N=19)***  Intervention (n= 9)   - African American: 4 - Latino: 3 - Asian American: 1 - Multiple ethnicities: 1   TAU (n=10)   - African American: 4 - Latino: 4 - Asian American: 0   Multiple ethnicities: 2 | - Session attendance: Treatment completed (out of 7 sessions): CEVE: 55%; TAU: 30% - Parent agency: Significant interaction of intervention condition and family, suggesting that CEVE families reported a significantly higher increase in parent agency compared to TAU interaction of intervention: condition x family, F(1,15) = 10.03; *p*<.01 - Alliance and Cultural Competence: Families receiving the CEVE reported higher alliance and perceived therapist cultural competence - Therapeutic Alliance: An effect for the Task subscale indicates that clients assigned to the CEVE reported higher ratings on this dimension than clients assigned to TAU (*p*<.001) - Across all three subscales, there was a main effect of condition, suggesting that the CEVE reported higher therapeutic alliance compared to TAU (Task: d=3.58; Bond: d=2.90 and Goal: d=2.16) - Cultural Competence: Significant main effects of condition were found for all subscales, suggesting that the CEVE had a significantly increased client-perceived therapist cultural competence |
| **Ye, J. et al. (2012)** | - Feasibility of telepsychiatry - Acceptability of telepsychiatry | Mixed Method Design  Congruent Triangulation:  Quantitative: cross-sectional study  Qualitative: Open-ended questions | ***Participants (N=16)***  all Korean | - Things liked about the video interview: Convenience and easy access; Language/cultural factors; personal factors of the telepsychiatrist (eg. the attitude of the provider) - Things disliked about the video interview: Technical factors of the device; Difficulties in establishing a rapport with the psychiatrist. - The ratings of acceptability of the telepsychiatry assessed by the process measure were high, ranging from 3.19 to 4.69 |
| **Yu, J. et al.**  **(2009)** | - Substance use - Social connectedness - Employment and education - Health, behavioral, and social consequences - Utility of the HIS model | Interrupted-time-series study (before-after) | *Participants screened (N=5621)*   - Chinese Americans (67.8%), - South Asian Americans: Indians, Bengalis, and Pakistanis (together 12.4%), - Koreans (11.9%) - Filipino (3.5%). - others Asians (less than 2%)   Screened positive (n=687)  Brief intervention (n=267)  Full intervention (n= 358) | - The results demonstrate a positive rate of change in five of six areas tested: - Substance use: the rate of change is 59.4% (*p* <.05) indicating more Asian clients did not use alcohol or drug within 30 days 6 months after treatment intake. Criminal justice area: rate of change is 11.9% (*p*< .05), indicating less involvement with the criminal justice system for 6 months after the completion of treatment. - Social connectedness: positive rate of change at 17.5% as compared to the national average of 5%. - Employment and education: positive rate of change is demonstrated at 10.1%, lower than the national average of 21% Stability of housing: no measurable change as compared to the national average rate of change that is reported at 10%. - Health, behavioral, and social consequences: the study demonstrated a 4% positive rate of change, showing an overall improvement in healthy living after treatment |
