# Supplementary material for: Culturally competent healthcare – A scoping review of strategies implemented in healthcare organizations and a model of culturally competent healthcare provision
Source: PLoS One. 2019 Jul 30;14(7):e0219971. doi: 10.1371/journal.pone.0219971 (PMC6667133; doi:10.1371/journal.pone.0219971)
Supplement: S4 Table — (DOCX) [file pone.0219971.s005.docx]

**S4 Table. Identified culturally competent components/strategies and categorization**

| **Authors** | **Type of intervention** | **Identified culturally competent components and strategies** | **Categorization** |
| --- | --- | --- | --- |
| Aggarwal, N. et al. (2015) | Use of Cultural Formulation Interview for the DSM-5 | - CFI in itself designed to be culturally sensitive | - Incorporation of culturally specific concepts in individual contacts |
| Alegría, M. et al. (2008) | The Right Question Project-Mental Health (RQP-MH) is a patient self-reported activation and empowerment strategy in mental health care | - Incorporation of cultural components that could influence minority patients’ experiences when taking an active role in care (not specified) | - Incorporation of culturally specific concepts in individual contacts |
| Anand, K. J. S. et al.  (2015) | Multilevel Health Care Delivery Intervention at pediatric intensive care unit (PICU) | - Education of health care professionals regarding culturally competent care - Recruitment efforts to increase the number of bilingual staff - Availability of 24-hour interpreter services in the ED and PICU - Translation of consent forms and educational materials for patients and families - Culturally sensitive end-of-life care discussions, with participation of palliative care services (not specified) - Outreach efforts and contacts with the Latino community to remove barriers to health care access - Help from the city government and local health department for preventive services | - Use of culturally and linguistically adapted written or visual material   - Educational and therapy written materials or   - Consent forms - Cultural competence training for healthcare providers - Human resources development   - Recruitment of bilingual and bicultural staff/oversea staff - Integration of interpreter services - User engagement and networking   - with target communities   - to reduce access barriers - Creating community health networks |
| Armengol, C. G. (1999) | Multimodal culturally sensitive and neuropsychologically informed support group addressing barriers to emotional, social, and vocational adjustment among high-level functioning Hispanic/Latino traumatic brain injury survivors | - Bicultural and bilingual provider - Incorporation of culturally specific themes (acculturation, stressors of the migratory experience - Incorporation of attitudes and belief about disability, health care and support networks - Use of dichos - Patterns of interaction common among less acculturated Hispanics/Latinos were followed - Integration of Hispanic values | - Linguistic and cultural matching - Incorporation of culturally specific concepts into individual contacts   - Patients’ problems, explanatory models   - General cultural values or norms   - Experiences caused by migration such as acculturation stress or racism   - Use of culturally specific language patterns |
| Aviera, A.  (1996) | “Dichos" Therapy Group: a therapeutic use of Spanish language proverbs with hospitalized Spanish-speaking psychiatric patients | - Bilingual provider - Use of Dichos to promote „cultural ambiance“ and help to deal with resistance and impasse | - Linguistic and cultural matching - Use of culturally specific language patterns |
| Barrio, C. et al.  (2010) | Culturally based family intervention for Spanish-speaking Latino families with a relative diagnosed with schizophrenia | - Integration of familism, spirituality and religiousness, nonjudgmental cultural attributions, biculturalism, and cross- border living and resources | - Incorporation of culturally specific concepts into individual contacts   - General cultural values or norms   - Experiences caused by migration such as acculturation stress or racism - Integration of families |
| Bekaert, S  (2000) | Minority integration and healthcare provision a hospital setting | - Formation of a multicultural consultation group planning and implementing interventions - Design of 5 year plan to lead to standardization of health care for ethnic minority groups - Invitation of the joint commissioning development manager for ethnic minorities in Oxfordshire to guide practice - Identification of staff’s needs (informal survey) - Record keeping and monitoring of episodes of contact with people from minority groups - Reference packs, containing general guidance (e.g. regarding diet, language, name, religious groups, birth, death, concepts of illness) - Identification of the main language groups of the area (survey) - Language and advocacy Services were made available - Provision of ethnic menus - Complaints procedure made available in all languages | - Creation of positions or groups to monitor and supervise the process - Integration of interpreter services - Adaption of the facility’s social and physical environment   - Make the complaints procedure available in all   - Providing cultural foods to increase well-being in clinic settings - Patient data collection and management:   - to monitor frequency of contact with patients from migrant groups - Needs assessment and monitoring of organizational changes   - assessment of provider needs or barriers   - Assessment of the main language groups - Development of action plans - User engagement and networking   - With institutions   - To obtain guidance |
| Bender, M. et al. (2013) | „Vida Saludable“: A culturally appropriate intervention to improve health behaviors in Hispanic mother-child dyads | - Trained (by investigators) bilingual promotora - Culturally adapted instrument measuring beverage consumption - Lessons were tailored, including visual   images of typical Hispanic families   - Cooking class with cultural foods - study materials were forward-translated into Spanish - Materials and curriculum were designed with special attention to culture, low literacy (less than 3rd grade) - Cultural adaptation based on stakeholders’ input, focus group feedback | - Cultural and linguistic matching - Integration of community health workers   - Educate patients during home or clinic visits - Use of culturally and linguistically adapted written or visual material   - Educational and therapy written materials or handouts   - translating the materials into different languages   - adapting materials to low literacy and education   - integrating illustrations of characters from target communities - Adaption of the facility’s social and physical environment   - Providing cultural foods used to educate participants about healthy eating - User engagement and networking   - target communities   - to reduce access barriers   - to assure cultural appropriateness |
| Beune, E. et al.  (2014) | Patient education intervention on blood pressure (BP) and treatment adherence for patient of African origin with uncontrolled hypertension | - Incorporating culturally-specific aspects of patients' perceptions (based on Arthur Kleinman) - culturally appropriate written educational materials (specific languages, customs, habits, norms and dietary cultures) - referrals to neighborhood facilities that support patients in adopting healthier lifestyles and are suitable for Surinamese and Ghanaian people - Nurse practitioner (NP) trained in - specific knowledge of hypertension in the Ghanaian / Surinamese communities and general cross-cultural counselling techniques | - Incorporation of culturally specific concepts into individual contacts   - Patients’ problems, explanatory models - Use of culturally and linguistically adapted written or visual material   - Educational and therapy written materials or handouts   - translating the materials into different languages   - including culturally sensitive treatment recommendations - Continuity of care   - Referral to specialized facilities which offer further culturally appropriate support - Cultural competence training for healthcare providers |
| Carrillo, J. E. et al.  (2011) | A regional health collaborative | - Four multiyear strategies: - Establishing patient-centered medical homes - Transforming clinics into medical homes - Medical home designation - Related Goals: building a workforce that could address the linguistic, cultural, and health literacy needs of patients (set up an Office of Care Management, improving access though a centralized contact center for information and appointment scheduling, improve cultural competency by employing bilingual and bicultural community health workers and “navigators” of the health system) - Information technology solutions have included the development of a personal health record for each patient, patient-specific disease dashboards, and a population-based disease registry. - Implementing a targeted care intervention: the targeted care intervention focused on the critical hospital-to-home transition period to minimize preventable readmissions - creating a “medical village”: (geographically defined community with a number of patient-centers medical homes linked to other providers and community-based resources) - collaboration with the New York City and State health departments to help local physician practices adopt electronic health record systems, transform themselves into patient-centered medical homes, and establish health information exchanges. | - Linguistic and/or cultural matching - Continuity of care   - Home visits   - Referral to specialized facilities which offer further culturally appropriate support - Integration of community health workers   - Help patients navigate the system - Human resources development   - Recruitment of bilingual and bicultural staff/oversea staff - Creating community health networks - Patient data collection and management   - better tailor care for individual patients |
| Chow, W. et al.  (2011) | Adapted assertive community treatment (ACT) model in Mount Sinai Hospital and KUINA Center | - Inclusion of bilingual and bicultural staff - Assignment of clinicians to patients on the basis of cultural and language requirements - Incorporation of cultural and ethnic elements into assessment and treatment plans (e.g. traditional Chinese medicines) - Promoting culturally appropriate supportive housing - Incorporating families in clients’ rehabilitation plans - Delivering multifamily psychoeducational groups - Prioritizing and incorporating cultural formulation and clients’ explanatory models - Staff training - Exchange with KUINA Center in Japan | - Linguistic and/or cultural matching - Incorporation of culturally specific concepts into individual contacts:   - Patients’ problems, explanatory models - Involvement of families - Human resources development   - Recruitment of bilingual and bicultural staff/oversea staff - User engagement and networking   - with facilities engaging in the same process   - to obtain guidance |
| Cooper, L. A. et al. (2011) | Patient-centered care and hypertension control in underserved primary care patients | - Trained community health workers (CHWs) administered the intervention - Physician communication skills training: Skills relevant to increasing patient engagement, activation - All patients received a monthly health   education newsletter designed to meet the needs of low literate adult readers | - Integration of community health workers   - to educate patients during home or clinic visits - Use of culturally and linguistically adapted written or visual material   - adapting materials to low literacy and education levels |
| Cooper, L. A. et al. (2013) | Patient-centered collaborative care interventions for depression among African Americans in primary care settings | - initial needs assessment (Standard assessment + questions on access barriers, attribution of illness, use of spirituality and concerns about treatment; social stressors and communication problems with health professionals) - Contact information for culturally sensitive psychotherapists as appropriate one-on-one telephone follow-ups by a Depression case manager (African American woman) - Educational material: culturally targeted materials designed to address barriers to depression treatment | - Cultural matching - Incorporation of culturally specific concepts   - Perceptions of access barriers into healthcare - Continuity of care   - Referral to specialized facilities which offer further culturally appropriate   - Telephone calls as reminders or follow-ups after or before an intervention - Use of culturally and linguistically adapted written or visual material   - Educational and therapy written materials or handouts   - by addressing barriers to care |
| Coronado, G. D. et al. (2011) | Clinic-based colorectal cancer screening promotion program | - Providers were Spanish-speaking - Mailed packet contained materials in English and Spanish - Telephone reminders - Educational home visit by health promoter (community members) and a medical assistant - All project materials were developed for a low literacy audience | - Linguistic and cultural matching - Use of culturally and linguistically adapted written or visual material   - educational and therapy written materials or handouts   - translating the materials into different languages   - adapting materials to low literacy and education levels - Integration of community health workers to educate patients during home or clinic visits - Outreach methods   - home visits   - telephone calls |
| Culica, D. et al.  (2008) | Community Health Worker as Sole Diabetes Educator (CoDE) | - Bilingual community health workers - Individual education visits, which addressed recommended diabetes knowledge and self-management skills - assessment and case management visits | - Integration of community health workers   - To educate patients during home or clinic visits |
| Dahhan, N. et al. (2012) | The Mosaic Outpatient Clinic (MOC) | - Patient centered consultation and exploration of culturally sensitive issues in the health care process - Mediators serve as the interface, translating language and interpreting culture, between the health care provider and the ethnic minority patient - Data collection of complete medical history, of demography, family situation, concepts, problems and experiences about healthcare and disease of parents - Development of parents’ profiles - Development of individual treatment plan (e.g. follow-up consultations, referrals) - Plan was discussed with patients and parents by the healthcare worker and supervisor together | - Incorporation of culturally specific concepts into individual contacts   - Perceptions of access barriers into healthcare - Involvement of families - Integration of community health workers   - Intermediate between patients and providers - Patient data collection and management   - better tailor care for individual patients |
| Delphin-Rittmon, M. E. et al.  (2016) | Bilevel cultural competence intervention | - Development of a bilevel training intervention for providers in cooperation with persons in recovery (PIR) with diverse ethnic backgrounds - Cultural competence training for staff members - Implementation of an ongoing cultural competence committee - Organizational level cultural competence assessment - Development of a cultural competence plan in consultation with the agency leadership, faculty and PIR that matched the agency’s capacity for implementation | - Cultural competence training for healthcare providers: - Needs assessment and monitoring of organizational changes   - Organizational level assessment - Creation of positions or groups to monitor and supervise the process - Development of action plans - Involvement of the facilities’ leadership - User engagement and networking   - with target communities   - to obtain guidance/consultation |
| Doorenbos, A. et al. (2011) | Calendar mail-out with cancer related health-message | - Native art each month matching cancer health-related message | - Use of culturally and linguistically adapted written or visual material   - Educational and therapy written materials or handouts   - integrating culturally specific art into intervention material - Outreach methods   - Mailed packages |
| Edwards, G. et al.  (2011) | WHO/Unicef Babyfriendly Hospital initiative in to increase breastfeeding rates | - Establishment of a steering group committed to the ethos of BFHI - Assessment of barriers to and knowledge on breastfeeding in hospital - Formulation of a 10 steps plan - Policies and procedures to support the changes in practice were developed - Development of an education program for all staff - Promotion of breast feeding - A set of booklets in different languages was produced - Flyer and DVD were made for women who may have trouble reading | - Use of culturally and linguistically adapted written or visual material   - Educational and therapy written materials or handouts   - Videos   - translating the materials into different languages   - adapting it to low literacy and education levels - Needs assessment and monitoring of organizational changes   - assessment of provider needs or barriers - Creation of positions or groups to monitor and supervise the process - Development of action plans - Promoting structural changes within the organization   - Adaptations of procedures and policies |
| Ferdinand, L.A.  (2009) | Patient-Centered Culturally Sensitive Health Care (PC-CSHC) Intervention Program | - Changing the physical health care clinic environment and clinic policies (culturally sensitive calendar, educational brochures, magazines, comment cards, bilingual restroom signs, posters, art featuring people from different cultures, bilingual policy brochures, displaying toys for patients’ children, DVDs and videos on health topics shown in the waiting area) - Training health care providers and office staff to engage in culturally sensitive behaviors and attitudes | - Use of culturally and linguistically adapted written or visual material   - Educational and therapy written materials or   - Policy brochures - Cultural competence training for healthcare providers |
| Galvin, S. et al.  (2008) | Project HELP (Hospital Education in Lactation Practices): Intervention to increase breastfeeding initiation among Cambodian women | - Creation of a Cambodian menu:   - Staff visit of the Cambodian market to consider nutritional content and the financial feasibility of purchasing foods   - The Cambodian interpreter cooked traditional recipes in the hospital kitchen, to educate the hospital chef, the director of food and nutrition, and hospital dietitians - The Cambodian menu was made available to all postpartum women | - Adaption of the facility’s social and physical environment   - to increase well-being in clinic settings |
| Garvin C.C. et al.  (2004) | A community based approach to diabetes control | - Support Groups: Contracting agencies established regularly scheduled support group meetings for diabetes patients and their support network. These group meetings have been tailored to meet the cultural needs of each of the 3 broad racial/ethnic groups provided in the patients’ primary language - Peer Education: One full time equivalent peer educator per racial/ethnic community facilitates support groups, assists in education and self- management classes, and arranges for the dissemination of diabetes materials in community settings. - Education Classes: topics were dealt with in a culturally relevant manner (focus on community concerns, cultural barriers, that arise when people try to make changes) - Enhanced Use of a Diabetes Registry: Software used to create diabetes registries within each of the participating community clinics, so that individuals who have not received appropriate services can be identified and targeted for interventions. - Case Coordination for Appropriate Patients using the Diabetes registry to improve and monitor patients’ care | - Linguistic and/or cultural matching - Involvement of families - Integration of community health workers   - Educate patients during home or clinic visits - Patient data collection and management   - better tailor care for individual patients   - to identify potential patients or individuals at risk - Incorporation of culturally specific concepts into individual contacts   - General cultural values or norms |
| Gary, T. L. et al.  (2009) | Nurse case manager and a community health worker team to improve care for patient with diabetes Mellitus Type 2 | - Telephone calls to remind participants about preventive health screenings - A written summary of their health care utilization was sent to the participant’s primary care provider - Participants received DM-specific information in the mail - individualized, culturally tailored care provided by a nurse case manager (NCM) and a community health worker (CHW) (not specified) - development of culturally tailored intervention action plans (IAPs) developed to address traditional cardiovascular risk factors and nontraditional obstacles to optimal DM care and self-management | - Integration of community health workers   - to educate patients during home visits - Outreach methods   - Home visits   - Mailed packages - Continuity of care   - Telephone calls as reminders or follow-ups after or before an intervention   - Communication with primary care provider - Patient data collection and management:   - To better tailor care for individual patients |
| Gerrish, K. et al.  (2004) | Adaptation program for overseas Registered nurses | - Period of supervised practice together with taught input - minimum of 10 weeks of supervised practice in a designated clinical area - Each overseas nurse was supported by one or more registered nurse mentors | - Human resources development   - Recruitment of bilingual and bicultural staff/oversea staff |
| Gil, S. et al.  (2016) | The Limited English Proficiency (LEP) Patient Family Advocate Role | - The LEP Patient Family Advocate role was created with the aim of improving access, promoting effective communication, and equalizing care for children with cancer from families with LEP. - Role 1: Specialized Medical Interpreter - Role 2: Cultural Liaison: The LEP advocate provides expertise and guidance to the health care team in the target-language’s culture(s), and to the family in the dominant U.S. culture and the culture of Western medicine. - Role 3: Healthcare Systems Advocate/Adjunct Case Management: The LEP advocate is readily available to assist the health care team with internal and external coordination across the continuum of care. | - Integration of community health workers   - to intermediate between patients and providers |
| Gilmer, T. P. et al.  (2005) | Project Dulce, a combined stepped-care diabetes nurse case management program and culturally oriented peer-led self-empowerment training program | - One-on-one visits: Initial visit with nurse and additional visits with nurse and dietitian - Telephone contact is used for appointment reminders and to answer specific questions - Group self-management training program: - curriculum delivered by trained peer educators (or promotoras) who are from the patient population, have diabetes themselves, and are of the same cultural/ethnic group as the participants. - Classes are taught in the patients’ native language | - Linguistic and/or cultural matching - Integration of community health workers   - to educate patients during home or clinic visits - Continuity of care - Telephone calls   - as reminders or follow-ups after or before an intervention |
| Goncalves, M. et al.  (2013) | The Portuguese Mental Health Program (PMHP) | - Bilingual and bicultural staff to serve children and adults from Brazil, Portugal, and Cape Verde - 95% of the providers speak Portuguese | - Linguistic and/or cultural matching |
| Halcon, L. L. et al.  (2010) | Health Realization,  a community-delivered, psychoeducational,  mental health intervention that focuses on resilience | - Culturally adapted and translated educational materials including visual cues for participants who did not read their native written language - socializing and sharing an East African dinner - intervention in English with concurrent translation - 15-minute prayer break was held promptly at sunset during each session - use of ethnographic methods as culturally appropriate ways to explain the concepts through traditional stories, pictures, proverbs, and faith-based metaphors and teachings | - Incorporation of culturally specific concepts into individual contacts   - Use of Specific culturally competent communication methods - Use of culturally and linguistically adapted written or visual material   - translating the materials into different languages   - adapting it to low literacy and education levels - Integration of interpreter services - Adaption of the facility’s social and physical environment   - Providing cultural foods as an opportunity for participants to socialize   - Integrated a 15-minute prayer break into support |
| Hamilton, L. J. et al.  (2013) | Pediatric Medical Home Program: primary care model focused on providing intensive care coordination for medically complex, ethnically diverse children with special health care needs | - Designated full-time, bilingual family liaison who acts as a bilingual healthcare system navigator, does triage of parents’ questions and concerns, ensures that the care plan is implemented, obtains outside medical records and insurance authorizations, forms relationships and interacts with community agencies - General pediatrician who develops, maintains, and manages a written care plan for each patient - Patients receive an ‘‘All About Me’’ Binder, which includes a problem list, care plan, medication list, and physician contact information, which is updated regularly - Family involvement in the development of the care plan and the program | - Linguistic and/or cultural matching - Involvement of families - Integration of community health workers   - Help patients navigate the system - Patient data collection and management   - to better tailor care for individual patients |
| Hatcher, S. et al.  (2016) | Culturally informed treatment in Maori who present to hospital after self-harm | - Therapists are Maori. - the process of therapy explicitly incorporates Maori cultural beliefs and values which we chose to describe using a powhiri (welcoming ceremony) model - Cultural assessment: establishing where both the therapist and the patient belong and what connections they have - Patient support for up to 2 weeks: 1-2 face-to-face or telephone sessions - Postcard contact for one year: Eight postcards were sent containing a short message offering support - Improved access to primary care: encouraging participants to attend their GP for a physical health check paying particular attention to cardiovascular risk factors especially alcohol and smoking | - Linguistic and/or cultural matching - Incorporation of culturally specific concepts into individual contacts   - General cultural values or norms - Continuity of care   - Telephone calls to offer further support   - Sending out postcards   - Communication or referral to primary care provider |
| Hudelson, P. et al.  (2014) | Migrant Friendly Hospital’’ Initiative | - Creation an interdepartmental and interprofessional working group (‘‘Health for All Network’’) - Creation of a reference-nurse post at the hospital for migrant care issues - Inclusion of patient language data in the electronic patient file, in order to facilitate timely identification of patients requiring interpreter services - Promotion of a national telephone interpreting service in 4 emergency services at the HUG, where access to face-to-face interpreters is rare - Brief presentation to all new staff during an obligatory staff orientation day about interpreter services and other ‘‘migrant friendly’’ services at the HUG - Development and dissemination of brochures containing information about the ‘‘Health For All Network’’; migrant friendly services at the HUG and when and how to work with an interpreter - Organization of a number of public events to bring attention to the Health For All Network and its activities | - Human resources development   - Creation of a new position as reference-nurse in charge of migrant care issues - Integration of interpreter services - Patient data collection and management   - better tailor care for individual patients - Creation of positions or groups to monitor and supervise the process - Promoting structural changes within the organization   - Brief presentations of changes/process to staff members   - Distributions of brochures presenting changes   - Public events promoting changes |
| Ivey, S.L. et al.  (2012) | Culturally and linguistically competent  health coach intervention for Chinese-American  patients with Diabetes | - All health coaches, the dietitian, and most physicians were ethnically and linguistically matched to their Chinese patients. - Pre-visit meeting: health coach meets with patient to complete routine assessments, data gathering, medication reconciliation, determination of patient agenda - Data entry: health coach enters data into chronic disease management database - Physician visit: Physician uses summary of patient’s current and historical data from the database - Post-visit meeting: health coach meets with patient to ensure that patient understands the care plan, assist patient in scheduling follow-up appointments, navigating the referral system - 3-month follow-up visit is scheduled with the primary care physician - Health coach makes follow-up phone call to patient - Recommendations took into account cultural implications - Physicians who did not speak their patient’s language would communicate with their patient through a clinic interpreter. | - Linguistic and/or cultural matching - Integration of community health worker   - to help patients navigate the system - Continuity of care   - Telephone calls as reminders or follow-ups after or before an intervention   - Referral to primary care provider - Patient data collection and management   - to better tailor care for individual patients - Incorporation of culturally specific concepts into individual contacts   - General cultural norms or values - Integration of interpreter services |
| Kalister, H. et al.  (1999) | Pharmacy-Based Treatment of Minor Illnesses | - Pharmacists are trained to evaluate and to treat children and adolescents aged with minor acute illnesses - Development of educational materials in English for each condition. The English pamphlets were then translated and published in 8 bilingual formats. | - Human resources development   - Expansion of the role of pharmacist to treating five minor pediatric conditions - Use of culturally and linguistically adapted written or visual material   - Educational and therapy written materials or handouts |
| Kanter, J. et al.  (2010) | Culturally Adapted Behavioral Activation (BAL) for Latinas with depression | - Bilingual provider - BAL manual was culturally adapted - the BAL manual listed free, low-cost, and culturally sensitive activation homework assignments - attention was paid to cultural values such as *familísmo, personalismo*, *marianismo*, and *machísmo* and how they influence activation - key BAL terms and therapy materials such as activity monitoring forms were translated into Spanish | - Linguistic and cultural matching - Incorporation of culturally specific concepts into individual contacts   - General cultural values or norms - Use of culturally and linguistically adapted written or visual material   - Educational and therapy written materials or handouts |
| Karmali, K. et al.  (2011) | Cultural Competence Initiative at the Hospital for Sick Children (SickKids) | - Establishment of the New Immigrant Support Network (NISN) to improve access to quality healthcare and partnered with multiple stakeholders, departments and worked closely with the senior management team - Cultural competence education to healthcare providers and managers - Translation of patient education materials in up to nine languages - Needs-assessment (organizational and provider level) - Cc learning modules were individually tailored for different staff groups - Placement and implementation of 12 kiosks that provide in multiple languages to help patients and families find their way around the hospital - Patient satisfaction survey was translated into seven languages to determine how effectively the hospital meets the needs of its population - Promotion of the use of face-to-face and telephone interpretation services - Establishment of a Champions Program: champions received advanced education in cultural competence and acted as change agents and role models - Allowing protected time for staff from different departments to attend the cultural competence education and training | - Creation of positions or groups to monitor and supervise the process - Involvement of the facilities’ leadership - Promoting structural changes within the organization - Use of culturally and linguistically adapted written or visual material   - Educational and therapy written materials or handouts   - Patient satisfaction surveys - Cultural competence training for healthcare providers - Integration of interpreter services - Adaption of the facility’s social and physical environment   - placement of twelve kiosks in a hospital offering multilingual help to patients and visitors - Needs assessment and monitoring of organizational changes   - Organizational level assessment   - assessment of provider needs or barriers |
| Kim, J. et al.  (2015) | Teleopthalmology service delivery model | - First Nations technicians and a trained teleopthalmology eye care nurse travelled to the remote First Nations communities in a truck that was dedicated to the teleopthalmology project - Adherence to First Nations’ cultural values as most important aspects of the project - Involvement of First Nations communities from the beginning - Exchange with the British Columbia Association of Optometrist - Naming of potential patients by participating First Nations communities - Phone calls from the ITHA team to schedule individual screening appointments during clinics to be held in their communities - All clients were contacted after their appointments and were mailed copy of their report - Capacity building opportunity to three First Nations youth and continued education | - Linguistic and/or cultural matching - Outreach methods   - Remote clinic - Continuity of care   - as reminders or follow-ups after or before an intervention   - Sending out postcards or mail - Incorporation of culturally specific concepts into individual contacts:   - General cultural values or norms - Human resources development   - Capacity building - User engagement and networking   - with institutions   - to reduce access barriers   - to obtain guidance/consultation - Patient data collection and management   - to identify potential patients or individuals at risk |
| Kline, K. N. et al.  (2016) | SHL-program (Sugar, Heart, and Life), a culturally sensitive entertainment-education telenovela for patients with diabetes mellitus | - Culturally sensitive narratives through focus groups (72 people) - English and Spanish versions - culturally sensitive characters and situations (Example of Hispanic families) - Special design considerations were made, including minimal use of on-screen text, narration by professional voiceover talent, and user-friendly navigation | - Use of culturally and linguistically adapted written or visual material   - Videos   - translation into different languages   - adapting it to low literacy and education levels   - integration of characters from the target community - Telemedicine   - offering education or prevention through videos |
| Kurth, A. E. et al.  (2016) | Linguistic and cultural adaptation of a computer-based counseling program (CARE+ Spanish) | - Intervention was delivered in Spanish - Cultural equivalence was assured through forward-back translation method and feedback from target group | - Use of culturally and linguistically adapted written or visual material - Telemedicine   - offering education or prevention through computer-based written information |
| La Roche, M. J. et al.  (2011) | A Culturally Competent Relaxation Intervention for Latino/as (CCRI) with anxiety | - Bilingual providers - Inclusion of allocentric (the tendency to define oneself in relationship to others) relaxation interventions, considered to be more appropriate for Latinos | - Linguistic matching - Incorporation of culturally specific concepts into individual contacts   - General cultural values or norms |
| Levin-Zamir, D. et al.  (2011) | Refuah Shlema: a cross-cultural program for promoting communication and health among Ethiopian immigrants | - Integrating Ethiopian immigrant liaisons in primary care as intercultural mediators - In-service training of clinical staff to increase cultural awareness and sensitivity - Health education community activities | - Integration of community health workers   - Intermediate between patients and providers - Cultural competence training for healthcare providers - Outreach methods   - Remote clinics |
| Mauldon, M. et al.  (2006) | Tomando Control, a culturally appropriate diabetes education program for Spanish-speaking individuals with diabetes mellitus | - Bilingual providers - Handouts and 2 nutritional guides specific to Latino diets (written at the fifth-to seventh-grade level) - Provision of culturally adapted and nutritionally sound meals - Use of written materials was minimized, and demonstration/return demonstration of skills was emphasized | - Linguistic matching - Use of culturally and linguistically adapted written or visual material   - Educational and therapy written materials or handouts   - adapting materials to low literacy and education levels   - including culturally sensitive treatment recommendations |
| McMurray, J. et al.  (2014) | Government assisted refugees (GARs) health clinic | - Partnership between a health clinic for GARs, local reception centre and community providers - Gateway services are provided by Reception House case workers and trained professionals - Comprehensive care is delivered by family physicians at the refugee health clinic - Language supports (interpreters) funded by Reception House - Ancillary services are delivered in the community by a variety of providers - Establishment of protocols and guidelines for diagnosis and management of diseases - Initial intake assessments performed onsite at Reception House within days of GARs’ arrival - international medical graduates in training whose input has enhanced the cultural sensitivity and competence of clinic processes | - Creating community health networks - Integration of interpreter services - User engagement and networking   - international medical graduates in training   - to assure cultural appropriateness |
| Mehler, P. S. et al.  (2004) | Language and cultural concordance in Russian patients with diabetes | - Arrival of a bilingual Russian internist trained in USA and Russia | - Linguistic and/or cultural matching |
| Melkus, G. D. et al.  (2004) | Culturally competent intervention of education and care for Black women with Type 2 Diabetes | - Two of the four nurses and the lay health assistant were black American women - Written materials and videotapes using illustrations and video characters of black Americans - Culturally specific recipes were incorporated and used as handouts - Feedback on material through focus groups and from the community advisory board of local black leaders and community members | - Linguistic and/or cultural matching - Use of culturally and linguistically adapted written or visual material   - Educational and therapy written materials or handouts   - Videos   - including culturally sensitive treatment recommendations   - integrating illustrations of characters from target communities - User engagement and networking   - target communities   - to assure cultural appropriateness |
| Menon, U. et al. (2008) | Interactive, computer based culturally sensitive education on colorectal cancer screening | - Culturally adaptation of the intervention based on focus group discussion with representatives of target population (not specified) | - Telemedicine   - offering education or prevention through computer-based written - Use of culturally and linguistically adapted written or visual material   - Educational and therapy written materials or handouts - User engagement and networking   - with target communities   - to assure cultural appropriateness |
| Moreno, F.A. et al. (2012) | Standard webcam telepsychiatry treatment of depression | - Bilingual psychiatrists | - Linguistic matching - Telemedicine   - offering treatment with a psychiatrist through webcam communication |
| Munoz, R. F. et al.  (2007) | Mamás y Bebés/Mothers and Babies Course: Health promotion group for prevention postpartum depression for Low-income Latinas | - Bilingual providers - Reinforcing values such as collectivism and familismo - Validating Latinas’ values and beliefs regarding pregnancy, childrearing practices, and motherhood - Addressing Latinas’ attitudes toward mental illness and seeking mental health services - participant course manual was written and adapted considering the sample’s education level and intra-group cultural, racial, and linguistic differences - Adhering to common cultural verbal and nonverbal communication norms - Validating the role of religion and spirituality in the health and healing of Latinas - Allowing to relate frustrations and painful experiences of discrimination and racism - Expanding Latinas’ knowledge without devaluing their cultural beliefs | - Linguistic matching - Incorporation of culturally specific concepts into individual contacts   - Patients’ problems, explanatory models   - General cultural values or norms   - following common verbal and nonverbal communication norms - Use of culturally and linguistically adapted written or visual material |
| Nowalk, M. P. et al.  (2008) | Individualized, culturally appropriate interventions to raise adult vaccination rates | - Immunization posters throughout the health center (also posters in Vietnamese and Spanish) and vaccination poster competition - Created a provider prompt for immunizations in its electronic medical record - Center held four influenza vaccination clinics in the surrounding neighborhood - Immunization quiz for clinical and clerical staff - Contest for the most prolific vaccinator - Vaccinators and vaccinees received a small treat at the time of vaccination | - Adaption of the facility’s social and physical environment   - Changing the facility’s physical environment - Patient data collection and management   - better tailor care for individual patients - Outreach methods   - Mailed packages   - Remote clinics - Promoting structural changes within the organization - Telemedicine   - offering education or prevention through videos |
| Ohr, S.O. et al.  (2016) | The transition of overseas qualified nurses and midwives into the Australian healthcare workforce | - The Overseas Staff Support Program aims to improve the experience of the overseas qualified staff - Support prior to arrival, on arrival and on commencement of their work | - Human resources development   - Recruitment of bilingual and bicultural staff/oversea staff |
| Oppong, B. et al.  (2016) | Culturally sensitive patient care paradigm | - Women who require additional workup after screening mammogram are assigned a patient navigator, who faciliates the diagnostic evaluation with further imaging or biopsies - There are two navigators and they reflect the population serviced by CBCC, Black and Hispanic with one being a fluent Spanish speaker | - Integration of community health workers   - to help patients navigate the system |
| Ortega, A. N. et al.  (2002) | Access to Community Care and Effective Services and Supports Program for homeless persons with severe mental illness | - Rely on principles of assertive community treatment (ACT) - Client-clinician ethnic and language matching | - Linguistic and/or cultural matching |
| O'Shaughnessy, R. et al.  (2012) | Sweet mother, mental health promotion group for West African women and their babies | - Culturally appropriate food - Providers are culturally trained | - Adaption of the facility’s social and physical environment   - Providing cultural foods used as an opportunity for participants to socialize - Cultural competence training for healthcare providers |
| Poureslami, I. et al.  (2016) | Video Education/self-management regarding inhaler use for Punjabi and Chinese patients | - Development of intervention materials using a community based participatory research approach, actively involving Punjabi and Chinese subjects - Translation of videos and all materials in 3 languages (Mandarin, Cantonese or Punjabi) - Cultural beliefs and practices from the 3 target communities were applied - Educators/Providers from target communities | - User engagement and networking   - target communities   - to assure cultural appropriateness - Cultural and linguistic matching - Incorporation of culturally specific concepts   - General cultural values or norms - Use of culturally and linguistically adapted written or visual material   - videos   - translating the materials into different languages Telemedicine   - offering education or prevention through videos |
| Reavy, K. et al. (2012) | C.A.R.E. (Culturally Appropriate Resources and Education): a clinic model for refugee health care | - Needs-assessment in focus groups with women from the refugee communities - Role of health advisor was created - Integration of certified medical interpreter - Languages spoken include Arabic, Burmese, Dari, Farsi, French, Karen, Kirundi, Lingala, Nepali, Pashtu, Russian, Somali, Swahili, and Uzbek (On average, 5 different non-English languages are spoken at each clinic) - Decoration with art from refugees’ native countries - Training of interested bilingual adults to take the medical interpretation certification test | - Needs assessment and monitoring of organizational changes   - assessment of patient needs - Human resources development   - Capacity building - Integration of community health workers   - Help patients navigate the system - Integration of interpreter services - Adaption of the facility’s social and physical environment   - decoration with art from refugee’s native countries |
| Redwood, D.G. et al.  (2016) | Alaska Native Colorectal Cancer (CRC) Family Outreach Program | - CRC cases are ascertained annually from the Alaska Native Tumor Registry - A CRC screening patient navigator uses the information to provide direct outreach to Alaska Native family members encouraging them to get screened for CRC using telephone and mailed reminders, scheduling them into the screening clinic, and guiding them through the cancer screening process - First-degree relative lists are also sent annually to regional Tribal Clinical Directors for use by their facilities in identifying patients at increased risk of CRC. - Asking to CRC patients for a contact list of their first-degree relatives. | - Integration of community health workers   - to help patients navigate the system - Outreach methods   - Mailed packages   - Telephone - Patient data collection and management   - to identify potential patients or individuals at risk |
| Riggs, E. et al.  (2017) | Culturally safe group pregnancy care | - Women had individual antenatal appointments (according to the hospital schedule) with a „caseload” midwife and either a telephone or on-site professional interpreter - Group information sessions: co-facilitated by the midwife, bicultural worker, and maternal and child health nurse. | - Integration of community health workers to   - Help patients navigate the system   - Intermediate between patients and providers - Integration of interpreter services |
| Rodrigue, J R. et al.  (2008) | Home-based education approach to increase Iive donor kidney transplantation | - The transplant surgeon and/or nephrologist talked to the patients about LDKT during a routine clinic visit - Patients attended a nurse-led education session with other transplant patients. Family members were also invited. - Patients also received written materials about LDKT and living donations - Patients received a home visit (interactive; e.g. “roundable” format) by transplant health educators, who met with the patient (and his/her invited guest; family members, friends, co-workers) to discuss LDKT and living donor donation. - Patients watched a brief 15-minute videotape on living kidney donation during home visit and also received written material - For black patients, a culturally sensitive educational approach was used: use of minority health educators and written brochures that highlight minority transplant recipients and their living donors and we integrated race-specific data into the discussion (e.g. unique transplant concerns of Blacks, specific LDKT rates) | - Linguistic and/or cultural matching - Use of culturally and linguistically adapted written or visual material   - Educational and therapy written materials or handouts   - integrating illustrations of characters from target communities - Telemedicine   - Videos - Outreach methods   - Home visits - Incorporation of culturally specific concepts into individual contacts   - Race specific data |
| Tolman, A. et al. (1998) | Implementation of a culture-specific intervention for a Native American community | - Needs-assessment with hospital administrator, a Native American social worker, three Native American patients and tribal elders - Installment of a Sweat Lodge on hospital property constructed by officially sanctioned representatives of the Reservation with appropriate materials according to Native traditions - Ceremonies at the Sweat Lodge were led by tribal elders and included Native American patients, Native and non-Native staff from the hospital and interested consumers | - Use of culturally and linguistically adapted written or visual material   - instalment of a sweat lodge - Needs assessment and monitoring of organizational changes   - assessment with patients, providers and representatives of target communities |
| Trinh, N.T. et al.  (2014) | Culturally Focused Psychiatric Consultation (CFP) Intervention for Latino Americans with Depression | The CFP consultation included two visits:   - Initial assessment by a clinician (psychiatrist or psychologist): - After engaging patients, clinicians reviewed with them their diagnoses and treatment recommendations - Follow-up after 2 weeks: visit with their CFP clinician to address their use of and questions about the toolkit. - Clinicians shared recommendations with primary care providers via e-mail. - Consultation provided in English or Spanish - Clinicians used the Engagement Interview Protocol (EIP) which integrates patients’ illness beliefs into psychiatric assessment and evaluation to improve the acceptance of psychiatric treatment among culturally diverse populations - All clinicians were trained specifically in the use of the EIP model - Clinicians develop co-constructed illness narratives with patients and reframe the Western concept of depression into more culturally resonant forms - Materials were also available on audio compact disc (CD) in English and Spanish. | - Linguistic and/or cultural matching - Incorporation of culturally specific concepts into individual contacts   - Patients’ problems, explanatory models   - Use of specific culturally competent communication methods (Illness narratives and EIP) - Use of culturally and linguistically adapted written or visual material   - Educational and therapy written materials or handouts - Integration of interpreter services - Continuity of care   - Follow-up in clinic visits   - Communication or referral to primary care provider - Cultural competence training for healthcare providers |
| Tu, S. P. et al.  (2006) | Health promotion for promoting fecal occult blood test (FOBT) screenings for Chinese patients | - Bilingual materials (a video, a motivational pamphlet, an informational pamphlet, and FOBT instructions) - Clinic-based education promoting fecal occult blood testing (FOBT) screening carried out by a trilingual and bicultural health educator | - Use of culturally and linguistically adapted written or visual material   - Educational and therapy written materials or   - Videos - Integration of community health workers   - Educate patients during home or clinic visits |
| Vargas, R.B. et al.  (2008) | The original patient navigation programs to reduce disparities in the diagnosis and treatment of breast cancer | - Patient navigation’ is an intervention that was designed and implemented to reduce disparities in breast cancer care for poor women in the largely African-American and Latino community of Harlem, New York City, in 1990. | - Integration of community health workers   - Help patients navigate the system |
| Watkins, E. L. et al.  (1990) | Model program to deliver primary healthcare services to migrant farmworker women and children | - The project emphasized coordinated services for migrant farmworker mothers and children - Collection of medical data of migrant farm worker women and children from medical records - Employment of multidisciplinary staff (two nurses, a nutritionist and a social worker all Spanish speaking) - Outreach and early case finding (Home visits to women enrolled in the project and provision of guidelines for identifying pregnant women and referring them to the center) - Coordination between the center’s programs and other health and social service agencies - Tracking system to encourage continuity of care: Migrant farmworker women and children were given copies of their health records, together with stamped postcards to notify center staff of their new location when they moved. - Prenatal patients were given a bilingual (Spanish-English) prenatal weightgain   Record and another bilingual record, to provide continuous data on the growth of each child.   - Health education program conducted to train migrant farmworker women as lay health advisors | - Linguistic and/or cultural matching - Use of culturally and linguistically adapted written or visual material   - Educational and therapy written materials or handouts - Continuity of care   - Giving out records/documents to patients to continue care themselves or at another place - Human resources development   - Capacity building - Patient data collection and management   - to identify potential patients or individuals at risk - Outreach methods   - Home visits - Creating community health networks |
| Weech-Maldonado, R. et al.  (2016) | National center for healthcare leadership diversity demonstration project | - Battery of preassessments - Diversity coach discussed the preassessment results with the leadership team - Development of organizational and individual level action plans in collaboration with leadership team and CEO - Executive coaching and training - Implementation of diversity leadership - Implementation of strategic human resource management - Promotion of patient cultural competency (provision of interpreter services and translated materials for limited English proficient patients, delivery of care, physical environment, and links to supportive services and providers) - Repetition of quantitative assessment battery to determine pre-post intervention change - Post-project feedback and planning for sustainable change and further improvements | - Use of culturally and linguistically adapted written or visual material   - Educational and therapy written materials or handouts   - adapting it to low literacy and education levels - Cultural competence training for healthcare providers - Human resources development   - Recruitment of bilingual and bicultural staff/oversea staff - Integration of interpreter services - Continuity of care   - Referral to specialized facilities which offer further culturally appropriate support - Needs assessment and monitoring of organizational changes   - Organizational level assessment - Creation of positions or groups to monitor and supervise the process - Development of action plans - Involvement of the facilities’ leadership |
| Wennerstrom, A. et al.  (2015) | Patient Resource and Education Program (PREP) to Support Disease Self-Management Among Vietnamese Americans | - Integration of bilingual community health workers (CHW) in patient-centered medical homes (PCMHs) - CHWs conducted home visits for each patient which included tailored health coaching - Activities of CHW included building patient self-management capacity through individual and family education, collaborative goal setting, providing links to supportive community resources, basic interpretation, patient and community advocacy and interacting with health care providers. - Adaptation wording of Vietnamese documents that were congruent with the local dialect - To accommodate limited literacy we used handouts with colorful diagrams and simple language and visual aids - CHWs called patients weekly to troubleshoot challenges and offer support | - Integration of community health workers   - Educate patients during home or clinic visits   - Intermediate between patients and providers - Use of culturally and linguistically adapted written or visual material   - Educational and therapy written materials or handouts   - translating the materials into different   - adapting it to low literacy and education levels - Continuity of care   - Telephone calls to offer further support - Outreach methods   - Home visits |
| Yasui, M. et al.  (2014) | Culturally Enhanced Video Feedback Engagement Intervention (CEVE) | - Shared understanding through cultural framing of the family’s problem in a culturally congruent manner based on shared observations - Cultural Ecogram (CE uses pictorial cues of a range of cultural and ecological influences) and Integrated Video Feedback | - Incorporation of culturally specific concepts   - Patients’ problems, explanatory models   - Use of specific culturally competent communication methods (CEVE) |
| Ye, J. et al. (2012) | Telepsychiatry service | - Korean-speaking psychiatrist | - Linguistic matching - Telemedicine   - offering treatment with a psychiatrist through webcam communication |
| Yu, J. et al.  (2009) | Culturally Adaptation of the Healthcare Intervention Services (HIS) Model to deliver substance abuse intervention services to Asian Americans | - interventionists who speak the language the client is most comfortable in - Screening: the Simple Screening Instrument (SSI) for alcohol-and drug abuse was translated into seven major Asian languages: Chinese, Korean, Japanese, Vietnamese, Hindi, Farsi, and Bengali. - Brief intervention: Clients assessed as “at risk” would receive a brief intervention. The interventionist specifically addresses any misunderstandings or barriers to treatment common among Asian Americans. A take home package in their own language. - Full intervention with a referral: Services offer clients with a substance abuse problem the opportunity to change the direction of their lives. - follow-up would provide the opportunity to assess the efficacy of the intervention and, if needed, an opportunity for further intervention. | - Linguistic and/or cultural matching - Incorporation of culturally specific concepts into individual contacts   - Perceptions of access barriers into healthcare - Use of culturally and linguistically adapted written or visual material   - Screening instruments   - translating the materials into different languages - Continuity of care   - Follow-up in clinic visits |
